# Synthesis, Antitumor Activities, and Apoptosis-Inducing Activities of Schiff’s Bases Incorporating Imidazolidine-2,4-dione Scaffold: Molecular Docking Studies and Enzymatic Inhibition Activities

**DOI:** 10.3390/ph18040496

**Published:** 2025-03-28

**Authors:** Fhdah S. Alanazi, Hamad M. Alkahtani, Alaa A.-M. Abdel-Aziz, Adel S. El-Azab, Hanadi H. Asiri, Ahmed H. Bakheit, Fatmah A. Al-Omary

**Affiliations:** Department of Pharmaceutical Chemistry, College of Pharmacy, King Saud University, P.O. Box 2457, Riyadh 11451, Saudi Arabia

**Keywords:** 5,5-diphenylacetophenone, EGFR2, HER2, molecular docking

## Abstract

**Background/Objective:** Cancer is the leading cause of death worldwide despite the diversity of antitumor therapies, which highlights the necessity to explore new anticancer agents. **Methods:** We synthesized 5,5-diphenylhydantoin derivatives including Schiff’s bases **7**–**27** and evaluated their cytotoxicity via the MTT assay. Enzymatic inhibition assays, cell cycle and apoptosis analyses, and molecular docking studies were also conducted. **Results**: Derivative **24** demonstrated the highest cytotoxic activity, with IC_50_ values of 12.83 ± 0.9 μM, 9.07 ± 0.8 μM, and 4.92 ± 0.3 μM against the cell lines HCT-116, HePG-2, and MCF-7, respectively. Compounds **10**, **13**, and **21** showed potent antitumor activities versus the examined cell lines (average IC_50_ = 13.2, 14.5, and 13.1 μM), respectively; moreover, these compounds also demonstrated promising EGFR and HER2 inhibitory activities, with IC_50_ values in the range 0.28–1.61 µM. Derivative **24** displayed the highest EGFR and HER2 inhibitory activity values (IC_50_ = 0.07 and 0.04 µM), respectively, which were close to those of the reference drugs erlotinib and lapatinib. Therefore, compound **24** was selected for further examinations and exhibited an inducing effect on apoptosis via diminishing the anti-apoptotic protein levels of BCL-2 (8.598 ± 0.29 ng/mL) and MCL-1 (261.20 ± 8.97 pg/mL) and promoting cell cycle arrest at the G2/M phase (33.46%). The binding relationships between compound **24** and the active sites of EGFR and HER2, which are similar to the co-crystallized inhibitors, were investigated using a molecular docking approach. **Conclusions**: These findings provide insights into the potential anticancer activities of the synthesized derivatives for further optimization to achieve therapeutic use.

## 1. Introduction

Cancer is increasingly recognized as a serious, worldwide health concern. It is estimated that cancer ranks as the second leading cause of mortality after cardiac illnesses [1,2]. Recently, resistance to existing cancer therapies has been considered a critical issue [3,4], and the resulting demand for new effective antitumor drugs poses a significant challenge to cancer research [5,6]. Tumor angiogenesis is responsible for the development and proliferation of tumors. The vascular endothelial growth factor (VEGF) family and its receptors may play a vital role in angiogenesis-dependent proliferation for many tumor types [7]. The overexpression of the epidermal growth factor receptor (EGFR) and the human epidermal growth factor receptor 2 (HER2) protein kinases leads to the stimulation of VEGF production and promotes angiogenesis in most human cancers [8,9]. Therefore, the inhibition of EGFR and HER2 indirectly prevents angiogenesis [10,11,12,13].

**Figure 1:**
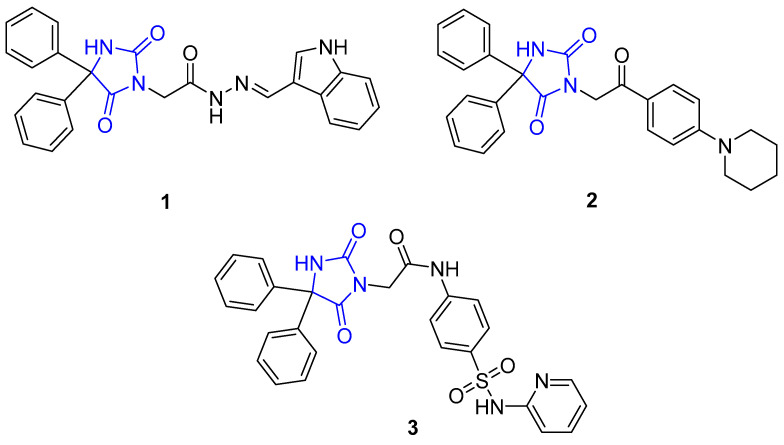
Diphenylhydantoin as anticancer derivatives.

Imidazolidine-2,5-dione (hydantoin) is a non-aromatic five-membered heterocycle [14] that is considered a pivotal scaffold in drug discovery because of its ability to accommodate a variety of substituents and undergoes well-established cyclization processes [15]. Additionally, it exhibits a wide array of applications, such as its antiproliferative and anti-metastatic activity, muscle relaxation capabilities, anti-inflammatory effects, and obesity and diabetes treatments, alongside its antimalarial, anti-HIV, virucidal, and pesticidal effects [16,17,18,19,20,21]. Previous studies have highlighted the anticancer activity of hydantoin, i.e., an anti-metastatic activity for phenylmethylene hydantoins [18,22]. One of the studies displayed the potency of hydantoin substituted with diphenyl moieties, compound **1** (Figure 1), demonstrating the kinase inhibition of EGFR, with an IC_50_ = 0.10 µM [23]. Another study demonstrated the potency of 5,5-diphenylhydantoin as an antitumor agent as derivative **2** (Figure 1) and showed a potent cytotoxic effect on various cell lines (IC_50_ = 10 µM) [24]. Furthermore, compound **3** displayed significant inhibition of the breast cancer cell line MCF-7 at around 74%, as presented in Figure 1 [25].

**Figure 2:**
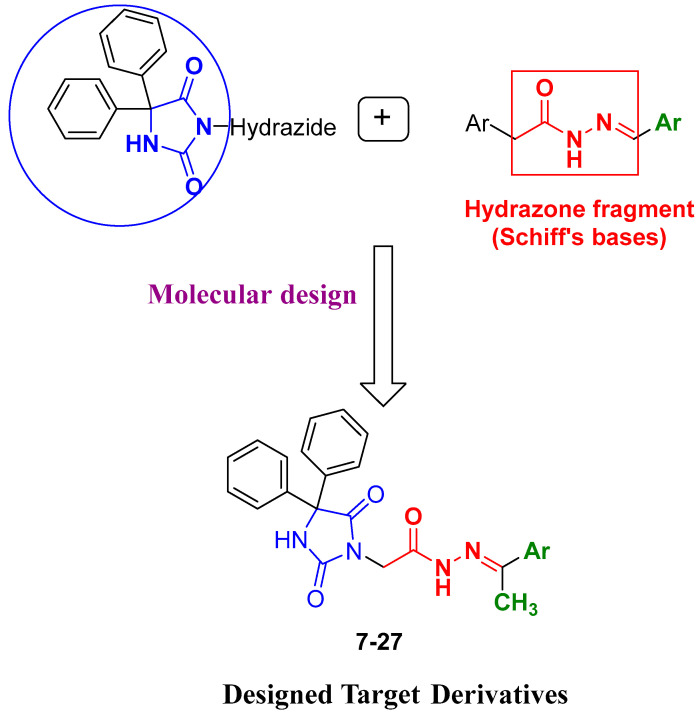
The reported and designed target Schiff’s bases associated with diphenylhydantoin with anticancer and kinase suppressive activities.

Compounds incorporating the azomethine linkage (-C=N-) present in Schiff’s bases provide immediate access to large libraries of molecular hybrids with structurally diverse biological properties as antitumor agents [26]. A hydrazide–hydrazone (−CO–NH–N=CH) compound is a type of Schiff’s base that displays considerable antitumor effects [27,28]. Based on previous reports, a series of hydrazide–hydrazone Schiff’s bases incorporating a hydantoin moiety were synthesized (compounds **7**–**27**) (Figure 2). Furthermore, the in vitro antiproliferative activities of these compounds were evaluated using four human cancer cell lines, and the structure activity relationship (SAR) of the derivatives was examined. For the most promising compounds, an enzymatic evaluation of the EGFR and HER2 inhibitory effects was performed, as well as the measurement anti-apoptotic markers and cell cycle analysis. Molecular docking simulations were generated to predict the affinity patterns of the active derivatives.

## 2. Results and Discussion

### 2.1. Chemistry

**Scheme 1:**
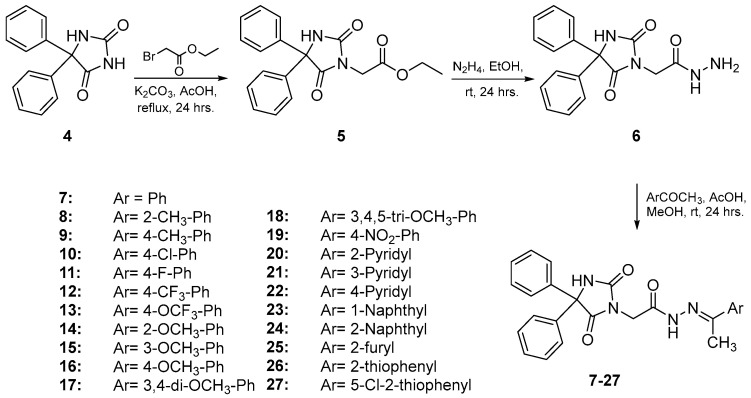
Synthesis of ketones conjugated with 5,5-diphenylimidazolidine-2,4-dione.

The process strategy for the intended compounds **7**–**27** is illustrated in Scheme 1. The structures of the newly synthesized compounds **7**–**27** were characterized using spectroscopic data (IR spectroscopy, ^1^H NMR, ^13^C NMR, and MS). Based on the reported method, a mixture of 5,5-diphenylhydantoin **4** and ethyl bromoacetate was subjected to heat in acetone containing anhydrous K_2_CO_3_ to produce an ethyl (5,5-diphenylhydantoin-3-yl)acetate **5** [29]. The resultant ester was refluxed with hydrazine in ethanol to obtain the hydrazide derivative **6**. As discussed previously, the generated hydrazide was stirred with an appropriate ketone at room temperature to afford the corresponding Schiff’s bases **7**–**27**.

The chemical structures of Schiff’s bases **7**–**27** were ascertained through the appearance of new peaks corresponding to phenyl ethylidene acetohydrazide (-CH_2_CONHN=C(CH_3_)-R in NMR. ^1^H NMR of compounds **7**–**27** showed methylene peaks (-CH_2_CONHN=C(CH_3_)-R) at δ 4.70–4.48 and 4.39–4.30 as two singlet peaks, indicating the formation of *E* and *Z* configurations, while ^13^C NMR revealed a methylene signal around 40.68–40.41 ppm. Two singlet signals resonating at δ 11.22–10.83 and 11.00–10.68 ppm in ^1^H NMR indicate the existence of the NH group of hydrazone (-CH_2_CONHN=C(CH_3_)-R). The methyl peaks (-CH_2_CONHN=C(CH_3_)-R) were identified for the three protons in the range of δ 2.45–2.22 and 2.41–2.19 ppm in ^1^H NMR and as two singlet peaks at δ 19.37–12.39 ppm in ^13^C NMR. Moreover, the three carbonyl peaks resonated at δ 174.17–174.01, 169.01–168.07, and 155.64–155.53 ppm in ^13^C NMR. A clear stretching band that corresponded to the C=O and NH groups, respectively, emerged at 1700 and 3190 cm^−1^, as verified using IR spectroscopy.

### 2.2. In Vitro Cytotoxicity Assay and SAR Study

The in vitro cytotoxicity of 2-(2,5-dioxo-4,4-diphenylimidazolidin-1-yl)-*N*′-ethylideneacetohydrazide derivatives, together with the reference compound erlotinib, was evaluated using the 3-(4,5-dimethylthiazol-2-yl)-2,5-diphenyltetrazolium bromide (MTT) assay on human colorectal carcinoma (HCT-116), hepatoblastoma (HePG-2), and breast adenocarcinoma (MCF-7) cell lines. A majority of the investigated compounds manifested significant antiproliferative potential, as presented in Table 1.

The structure activity relationship (SAR) analysis of the derivatives of 2-(2,5-dioxo-4,4-diphenylimidazolidin-1-yl)-N′-ethylideneacetohydrazide revealed pivotal insights when tested against three cell lines: MCF-7, HCT-116, and HePG-2. The unsubstituted phenyl in compound **7** acted as our baseline for most of the synthesized compounds, displaying IC_50_ values of 38.30 μM, 47.46 μM, and 55.81 μM across the MCF-7, HCT-116, and HePG-2 cell lines, respectively. Phenyl’s fundamental significance in these molecular activities is reflected by the marginally increased sensitivity in MCF-7 cells. Halogen substitutions showed enhanced potency. Specifically, 4-chlorophenyl, substituted in compound **10**, reported strikingly low IC_50_ values at 11.18 μM, 17.90 μM, and 10.69 μM for the MCF-7, HCT-116, and HePG-2 cell lines, respectively. Similarly, compound **11** with 4-fluorophenyl showed a low IC_50_ in the same sequence of cell lines at 14.25 μM, 28.33 μM, and 21.06 μM. Such potent figures indicate that the halogens’ electron-withdrawing capacity might bolster the molecule’s reactivity, leading to enhanced interactions with its biological targets. Electron-withdrawing groups also exhibited superior performance. The 4-(trifluoromethoxy)phenyl-substituted derivative **13** emerged as a potent candidate, with IC_50_ values of 9.58 μM, 20.11 μM, and 13.94 μM across MCF-7, HCT-116, and HePG-2, respectively. This provides compelling evidence that the trifluoromethoxy group can enhance the compound’s electrophilicity.

The impact of methoxy substitutions varied according to their number and position. Notably, compound **17**, featuring the 3,4-dimethoxyphenyl substitution, was remarkably active, boasting IC_50_ values of 15.71 μM, 26.75 μM, and 19.63 μM for the MCF-7, HCT-116, and HePG-2 cell lines, respectively. This reflects the vital role of the methoxy groups’ orientation in adjusting both the steric and electronic impacts.

The pyridinyl derivatives particularly stood out, with the pyridin-3-yl in compound **21** showing admirable IC_50_ values at 8.61 μM, 19.25 μM, and 11.50 μM across MCF-7, HCT-116, and HePG-2, respectively. In addition, the pyridin-4-yl compound **22** exhibited IC_50_ values at 13.11 μM, 25.96 μM, and 17.18 μM across MCF-7, HCT-116, and HePG-2, respectively. This potency may be attributed, in large part, to the unique electronic contributions that the nitrogen atom of the pyridine ring makes.

Most strikingly, the naphthalen-2-yl-substituted molecule in compound **24** demonstrated profound potency, especially in the MCF-7 cell line. It registered an IC_50_ value of a mere 4.92 μM, which is the most potent derivative in contrast to erlotinib, the reference drug with an IC_50_ value of 5.18 μM. In addition, compound **24** exhibited excellent potency against HCT-116 and HePG-2, with corresponding values of 12.83 μM and 9.07 μM, respectively. Such efficiency could be attributed to its amplified aromaticity, potentially assisting through π–π stacking interactions. Interestingly, heterocyclic compounds such as compound **26** with thiophen-2-yl substitution presented moderate to less effective values. While this compound showed a higher IC_50_ of 76.83 μM in MCF-7, its values in HCT-116 and HePG-2 were more moderate at 87.50 μM and 32.26 μM, respectively.

Erlotinib, the reference compound, consistently exhibited superior potency, recording IC_50_ values of 5.18 μM, 8.53 μM, and 7.90 μM across MCF-7, HCT-116, and HePG-2. This not only exemplifies its therapeutic potential but also furnishes a comparative standard. The proximity of some compounds, such as compound **24**, to this benchmark points to their promising therapeutic prospects.

### 2.3. In Vitro Cytotoxicity Against Normal Human Cells (WI-38)

The cytotoxicity of the most active derivatives **10**, **13**, **21**, and **24** were further evaluated to determine their effects on the WI-38 normal fibroblast cell line to display the selective cytotoxicity toward normal and malignant cells. Table 2 illustrates the cytotoxicity results of the tested compounds **10**, **13**, **21**, and **24**, which demonstrated low cytotoxicity; the comparative IC_50_ value for doxorubicin was 6.72 μM. The other reference drug erlotinib (IC_50_ = 46.52 µM) showed a lower toxic effect on WI-38 than compounds **13** and **24** (IC_50_ = 45.39, 39.52 µM), respectively. Compounds 10 (IC_50_ = 54.21 µM) and 18 ((IC_50_ = 60.79 µM) exhibited lower cytotoxic results than the reference drugs.

Selective cytotoxicity of the synthesized compounds against cancer cell lines rather than normal cell lines was evaluated by examining them against normal cells WI-38 to confirm that the compounds were selectively targeting cancer cells. The selectivity was translated using high selectivity index (SI) values of 2.2–8.0 for the entire tested cancer cell lines close to SI values of 5.4–8.9 for erlotinib, confirming their high safety profile. Among the tested compounds, the most selective compound toward the MCF-7 cell line was **24**, with an SI value of 8.0, which is close to 8.9, the value of erlotinib. Moreover, compounds **10**, **13**, and **21** showed high SI values of 4.8, 4.7, and 7.6, respectively. Regarding the HePG-2 cell line, compounds **10**, **13**, **21**, and **24** showed SI values of 5.0, 3.2, 5.2, and 4.3, respectively, compared with 5.8 for erlotinib. In respect to HCT-116, **10**, **13**, **21**, and **24** showed SI values of 3.0, 2.2, 3.1, and 3.0, respectively, in comparison with 5.4 for erlotinib. Compound **24** showed the safest and most selective results [SI = 4.35 (HePG-2), 8.0 (MCF-7), and 3.0 (HCT-116)].

### 2.4. Kinase Inhibition Activity

The most active compounds—**10**, **13**, **21**, and **24**—were tested in vitro for their ability to inhibit EGFR and HER2. Erlotinib was probed as the standard drug in the EGFR inhibition assay, while lapatinib was tested as the reference drug in the HER2 inhibition assay. The inhibitory effects of compounds **10**, **13**, **21**, and **24** and the reference drugs are summarized in Table 3. Schiff’s bases encompassing a 2-naphthalene moiety, such as compound **24**, were considered the most active in comparison with EGFR and HER2 (IC_50_ = 0.07 and 0.04 µM, respectively), which had similar values to those of the reference drugs erlotinib (EGFR-IC_50_ = 0.05 µM) and lapatinib (HER2-IC_50_ = 0.03 µM). The 4-triflouromethoxyphenyl **13** and 4-chlorophenyl **10** compounds showed similar inhibitory activities against EGFR (IC_50_ = 0.61 and 0.67 µM, correspondingly). Compound **13** displayed higher HER2 inhibitory activity (IC_50_ = 0.28 µM) than compound **10** (IC_50_ = 0.71 µM). Compound **21** was the least active against EGFR and HER2 (IC_50_ = 1.61 and 1.17 µM, respectively). Interestingly, these surprising results could reflect the cytotoxic effects on the examined cell lines, which contributed to the excellent EGFR and HER2 inhibitory effects of compound **24** and other derivatives.

### 2.5. Measuring the Expression of Anti-Apoptotic Proteins (MCL-1 and BCL-2) in MCF-7 Cells

**Figure 3:**
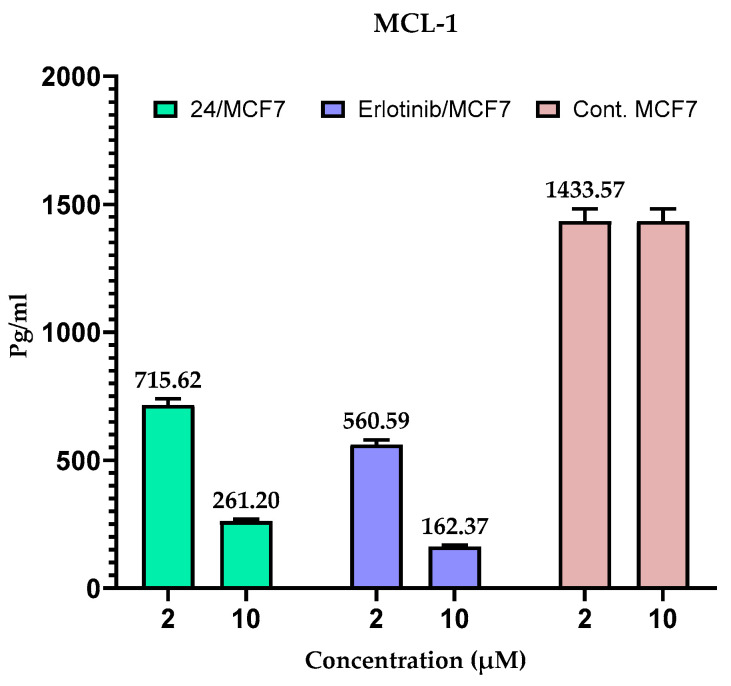
The effect of compound **24** and the control drug (erlotinib) on the expression level of the anti-apoptotic protein MCL-1 in MCF-7.

**Figure 4:**
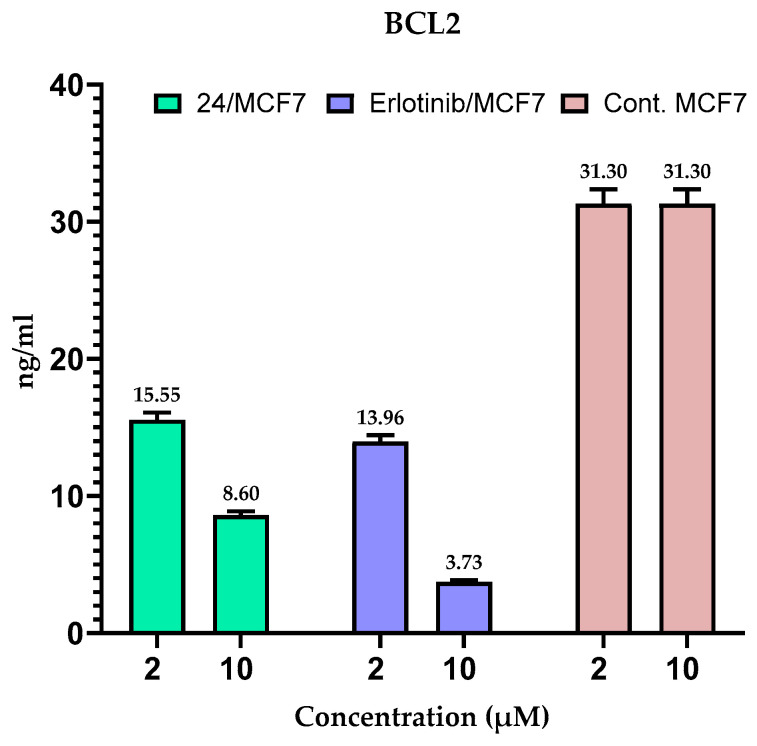
The effect of compound **24** and the control drug (erlotinib) on the expression level of the anti-apoptotic protein BCL-2 in MCF-7.

Apoptosis is regulated by intrinsic pathways such as the B-cell lymphoma-2 (BCL-2) family [30]. BCL-2 proteins are probably essential for successful tumor cell survival, and thus, the inhibition of these anti-apoptotic proteins suggests a promising approach to cancer therapy [31,32,33]. MCL-1, one of the BCL-2 family members, is likely to have a significant role in cell death [31]. The expression of MCL-1 and BCL-2 in MCF-7 cells treated with different concentrations was studied. In several samples, such as **24** and the control drug, erlotinib, the expression levels of MCL-1 were down-regulated in a dose-dependent manner for all compounds; in particular, compound **24** decreased to 261.20 ± 8.97 pg/mL, with maximum expression detected in the control cells (1433.57 ± 49.2 pg/mL) (Figure 3). Additionally, the concentration-dependent decrease in BCL-2 expression (8.598 ± 0.29 ng/mL) can be observed when increasing the compound concentration; hence, maximum baseline expression was observed in the control MCF-7 cells (31.300 ± 1.07 ng/mL) (Figure 4). Indeed, the important effect of compound **24** was clearly demonstrated in the induction of apoptosis.

### 2.6. Cell Cycle Arrest Analysis

Cell cycle analysis is used to quantify DNA content distribution via flow cytometry at various cell cycle stages (pre-G1, G1, S, and G2/M) [34]. Compound **24** was selected to analyze its effect on cell cycle progression in the MCF-7 cell line at different concentrations. As a result, the percentage of the apoptotic cells increased at the G2/M phase from 7.82% (MCF-7 cells) in the control sample to 25.43 (at 2 µM) and 33.46 (at 10 µM), as well as the effect of the reference drug, erlotinib. Therefore, cell cycle arrest appeared at the G2/M phase in cells treated with compound **24** and erlotinib (Table 4). This result illustrates the significance of compound **24** in the induction of apoptosis and cell cycle arrest. These results further support the antiproliferative activity of compound **24**, which is consistent with EGFR and HER2 inhibition.

### 2.7. Drug Likeness

The drug likeness of compounds was calculated as indicated by Lipinski’s rules, which point out potential therapeutic benefits [35]. In the presented data, key parameters such as the Molecular Weight (MW), hydrogen bond attributes, and Topological Polar Surface Area (TPSA), as well as the solubility (LogS), distribution (LogD), and partition (LogP) logarithmic values, were analyzed for 2-(2,5-dioxo-4,4-diphenylimidazolidin-1-yl)-N′-ethylidene acetohydrazide derivatives using ADMETlab 2.0. Most compounds have MW values between 400 and 500 Da, fitting within Lipinski’s criteria. The hydrogen bond counts and TPSA values suggest favorable drug-like properties and potential cell permeability, respectively. Compounds **10** (with a 4-chlorophenyl group) and **24** (with a naphthalen-2-yl substitution) demonstrate promising pharmacokinetic characteristics.

### 2.8. ADMET Properties

The pharmacokinetic properties and toxicological profiles of potential drug candidates play a pivotal role in determining their suitability for further development [36]. Here, we interpret the ADMET (Absorption, Distribution, Metabolism, Excretion, and Toxicity) data of the investigated compounds, generated using the online tool ADMETlab 2.0.

The derivatives **7**–**27** predominantly display negative Caco-2 permeability values, expressing potential challenges in oral bioavailability. Although compound **26** stands out as an anomaly due to its noticeably high HIV value, signifying greater intestinal absorption, this result is corroborated by their low Madin Darby Canine Kidney (MDCK) and Human Intestinal Absorption (HIA) permeability levels. Moreover, most of these compounds show a high likelihood of interacting with P-glycoprotein (Pgp), both as inhibitors and substrates, which can significantly impact their absorption and efflux from cells. These interactions might alter the actual bioavailability of the compounds, despite the limited absorption suggested by other metrics.

Compounds **7**–**27** show a range of values for the volume of distribution at steady state (VDss), blood–brain barrier (BBB) penetration, and plasma protein binding (PPB). The VDss values, which predict the distribution of a drug between the plasma and the rest of the body, vary across the compounds. Compound **25** showcases the highest VDss at 1.049, suggesting a broader distribution in the body. The BBB values are indicative of a compound’s ability to penetrate the central nervous system and are predominantly high for most compounds, such as compound **4**, with a value of 0.99. In other words, these compounds have the potential to cross the BBB. However, compounds **18**, **19**, **23**, and **24** have relatively lower BBB values, implying limited brain penetration. The plasma protein binding (PPB) values are predominantly close to 1, suggesting high binding affinity, which might impact free drug concentrations in plasma. For instance, compound **24** exhibits a PPB of 0.99, indicating that a significant portion of the compound might be protein bound.

Derivatives **7**–**27** are likely substrates for the key CYP isozymes 1A2, 3A4, 2C9, and 2C19, as indicated by values above 0.5. Additionally, many derivatives show potential as inhibitors for these enzymes. This dual behavior suggests possible metabolic interactions when co-administered with other drugs. Furthermore, the clearance (CL) and half-life (T⅟_2_) metrics provide insights into drug elimination. For instance, compound **24** has a CL of 0.84 and T⅟_2_ of 0.425, which suggests a low rate of elimination and a moderate half-life.

### 2.9. Molecular Docking Studies

The molecular docking modeling technique was employed to investigate the molecular configuration and SARs of the most potent derivatives, along with their interactions within the binding pocket of the target receptor or enzyme [37,38,39,40].

#### 2.9.1. Validation of Docking Approach

**Figure 5:**
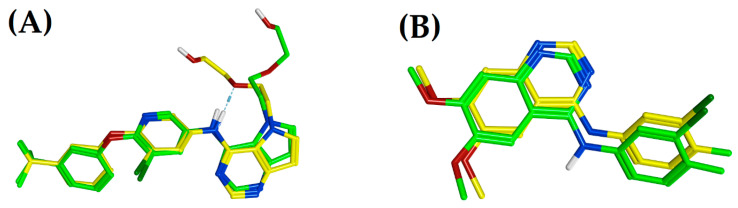
Overlay of the redocked (green) and co-crystallized ligand 03Q (yellow) within the HER2 binding site (**A**) and the redocked ligand (green) and co-crystallized ligand Gefitinib (yellow) within the EGFR binding site (**B**). The close alignment of the redocked and co-crystallized ligands validates the docking protocol and confirms the reliability of the binding pose predictions.

To assess the reliability of the docking protocol, the co-crystallized ligand 03Q was redocked into the HER2 active site. The redocking analysis demonstrated a strong hydrogen bond between the oxygen atom (O1) of 03Q and the OD2 atom of Asp863, emphasizing the critical role of the DFG motif in ligand binding. Additionally, 03Q established hydrogen bonds with key residues, including Gln799, Leu796, and Met801, suggesting a broad interaction network within the kinase domain. The ligand’s polycyclic ring system also engaged in pi-H interactions with multiple residues, reinforcing its accommodation within the hydrophobic pockets of the binding site. Notably, interactions with Leu785, Leu800, Leu852, and Phe864 further stabilized the ligand within the kinase domain. The redocking process yielded a Root Mean Square Deviation (RMSD) of 1.3633 Å, confirming the accuracy of the docking methodology. The overlay of the redocked and co-crystallized conformations of 03Q (Figure 5A) further supports the robustness of the docking approach.

To validate docking accuracy further, the co-crystallized ligand was redocked into the epidermal growth factor receptor (EGFR) active site, yielding an RMSD value of 0.593 Å, which is within an acceptable threshold, indicating a reliable docking procedure. In contrast, the co-crystallized ligand IRE exhibited a distinct interaction profile within the EGFR binding site (Figure 5B overlay of the redocked co-crystallized ligand Gefitinib). IRE formed a hydrogen bond with Gln791, contributing an interaction energy of -0.5 kcal/mol, whereas a stronger interaction was observed with Met793, with an energy of -4.7 kcal/mol. Additionally, IRE engaged in aromatic pi-H interactions with Leu718, Lys745, and Leu792, which likely play a pivotal role in stabilizing the ligand within the active site and enhancing its binding affinity.

These findings collectively support the validity of the docking protocol, ensuring its reliability in predicting ligand–receptor interactions.

#### 2.9.2. Molecular Docking

Molecular docking was carried out via the MOE program version 2015.1 (Chemical Computing Group Inc., Montreal, QC, Canada). The co-crystallized bound inhibitor, which was retrieved from the RCSB Protein Data Bank (RCSB PDB) [41], and the prospective multitarget agent 24 were exposed to docking methodology within the EGFR and HER2 tyrosine kinase binding pockets to generate an appropriate interaction orientation and precise docking [42,43].

##### Docking of Compound **24** into HER2

**Figure 6:**
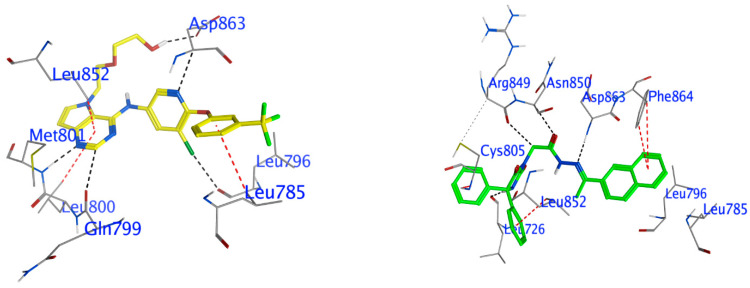
Three-dimensional representation of the HER2 (PDB code: 3PP0) binding site showing compound **24** (depicted in green) in juxtaposition to the co-crystalline ligand (03Q) colored in yellow. The spatial orientations provide insights into their respective interactions and alignments within the active site.

Considering the interaction profile for compound **24** with HER2, we note a series of significant interactions (Figure 6, right panel). The hydantoin moiety of compound **24** forms a hydrogen bond with Leu726, located within the glycine-rich nucleotide phosphate-binding loop of the N-lobe, which might suggest that compound **24** competes directly with ATP binding. This occurs because this interaction mimics the typical backbone interactions often observed with the adenine segment of ATP. Another significant interaction involves hydrogen bond interaction with Arg849 and the methylene chain. This residue is part of the catalytic loop in the C-lobe, hinting that the compound may affect the kinase’s catalytic mechanism. The hydrazide–hydrazone linkage in compound **24** also has two hydrogen bond interactions. One of these hydrogen bonds is with Asp863, which is a residue in the DFG motif. The importance of this motif in kinase activation suggests that this interaction might stabilize HER2 in its inactive form. The other hydrogen bond interaction is with Asn850, which is positioned in the catalytic loop. This bond further underscores the potential of compound **24** to interfere with the kinase’s catalytic process. Lastly, the compound exhibits pi-H interactions with residues Cys805, Leu852, and Phe864. These interactions point to a deep engagement of compound **24** within the ATP pocket, potentially improving its potency and selectivity due to the optimization of its fit within the pocket (Table 5).

The 3D structure of co-crystalline ligand 03Q with HER2 was obtained from the PDB (PDB code: 3PP0) (Figure 6, left panel). The ligand interactions mirrored those of compound **24**. This overlap in interactions implies that the binding modes and potencies of the two compounds might be closely aligned. If 03Q is a known inhibitor, these similar interaction profiles can offer deeper insights into the potential mechanism of action for compound **24**.

##### Docking Other Derivatives into HER2

Compounds **7**–**27** are designed to interact with the HER2 kinase domain by occupying the canonical ATP-binding cleft, which is formed between the predominantly β-stranded N-lobe and the mostly α-helical C-lobe. The two lobes are connected by a hinge region, and in HER2, this often involves Met793 and Leu792 as key contact points for hydrogen bonding. Such hinge interactions are crucial for conferring both affinity and specificity in kinase inhibitors.

Among the key residues in the binding site, Met793 (located in the hinge) is especially important, as most of the compounds form one or more hydrogen bonds with this residue. Several compounds (e.g., **7**, **11**, **14**, **15**, and **23**) (Figure S1) also form hydrogen bonds with Thr854, situated near the activation loop, and Cys775, located at the αC-helix terminus, which helps stabilize the ligand in the ATP pocket. In addition, many entries show π–H interactions with Leu718, Leu844, and Val726, which are hydrophobic residues around the catalytic loop that provide an anchoring surface. Lys745, positioned in the β3-strand/αC region, is another site of interest: it can form π–cation interactions or hydrogen bonds, reflecting its usual role in stabilizing ATP’s phosphate groups in active kinases.

Overall, two main categories of binding interactions emerge from these docking results: hydrogen bonds (including those at Met793, Leu792, Thr854, and Cys775) and π–H/π–cation contacts. The aromatic rings of the inhibitors frequently engage hydrophobic residues (Leu718, Leu844, and Val726) via π–H interactions, while Lys745 can participate in π–cation binding. These combined interactions—particularly hinge hydrogen bonds plus additional hydrophobic or electrostatic contacts—enhance the overall binding affinity.

In terms of binding energies, the docking scores (S) range roughly from −7.07 to −8.21 kcal/mol, indicating moderate-to-strong predicted affinities across the series. Compounds that form multiple strong hydrogen bonds (especially those targeting both Met793 and Thr854) and additional π–cation or π–H contacts (such as compounds **17**, **18**, **19**, and **23**) tend to reach more favorable docking scores, around −8.0 kcal/mol or better (Table S4).

These findings align with well-established critical sites for HER2 kinase inhibition. The hinge region around Met793 remains the primary anchor for small-molecule inhibitors, while interactions near the αC-helix (Cys775, Lys745) and the activation loop (Thr854) further contribute to modulating the kinase conformation. The hydrophobic platform formed by Leu718 and Leu844 near the catalytic loop helps stabilize the aromatic moieties of the inhibitors. Altogether, the table shows that all tested compounds exploit typical kinase “hot spots” in HER2—especially the hinge region—and that additional contact with Thr854, Cys775, and Lys745 can further strengthen binding.

##### Docking of Compound **24** into EGFR

**Figure 7:**
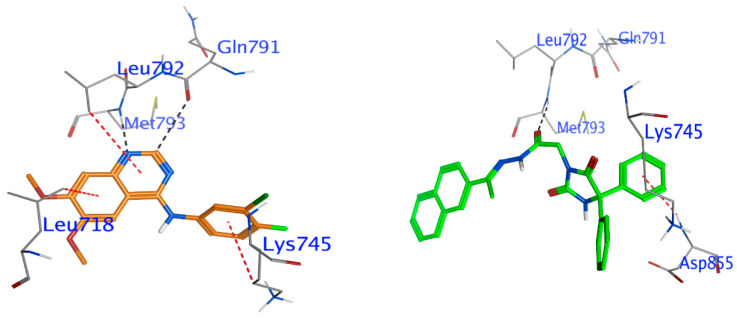
Three-dimensional (3D) representation of the EGFR kinase region showcasing the binding interactions of compound **24** (depicted in green) and the co-crystalline ligand (PDB code: 2ITY) rendered in brown. The distinct spatial orientations and interaction points highlight their respective inhibitory mechanisms within the ATP-binding cleft.

Compound **24** interacts with Met793 via a hydrogen bond (Figure 6, right panel), suggesting that it can impact the kinase’s function by interfering with this region. Met793 lies close to the ATP-binding site, which might have an impact on the activity of compound **24**. Moreover, the compound exhibits a pi-H interaction with Asp855, a residue in the DFG motif, indicating its potential role in affecting the DFG conformation, which is crucial for the kinase’s activation state (Figure 7 and Table 5).

The crystalline structure of EGFR (PDB code: 2ITY) in complex with Gefitinib (Figure 7, left panel) was retrieved from the RCSB Protein Data Bank. While IRE interacts with Gln791 and Met793 via hydrogen bonds, the strong interaction with Met793, as evidenced by the energy of −4.7 kcal/mol, signifies its vital role in IRE’s inhibitory action. The pi-H interactions of IRE with Leu718, Lys745, and Leu792 further highlight its anchoring in the active site and possibly interrupt ATP binding. Leu718 is part of the glycine-rich nucleotide phosphate-binding loop, and Lys745 is a conserved residue essential for kinase activity.

Both compound **24** and IRE interact with the ATP-binding site; however, their interaction profiles slightly differ. IRE seems to have a broader interaction spectrum, engaging with residues from the N-lobe (such as Lys745) and residues near the ATP-binding site (such as Met793). Compound **24**, on the other hand, specifically targets Met793 and Asp855, which can potentially influence the DFG conformation and, by extension, kinase activation. Describing the orientation is challenging without the aid of 3D visualization. However, based on the interactions, it appears that IRE might be more extended within the ATP-binding pocket, interacting with both the phosphate-binding loop and residues crucial for kinase activity. In contrast, compound **24** seems more targeted, focusing on the ATP-binding site’s immediate vicinity and the DFG motif.

##### Docking Other Derivatives into EGFR

Compound **7** engages the EGFR target by establishing multiple interactions within the ATP-binding site. It forms two hydrogen bonds with residues in the hinge region and adjacent areas (Figure S2): one from its N-terminal group to the OG1 of Thr854 (3.08 Å, −1.4 kcal/mol) and another from its C-terminal group to OD2 of Asp855 (3.14 Å, −0.3 kcal/mol). In addition, it accepts a hydrogen bond from the backbone nitrogen of Met793 (2.94 Å, −2.3 kcal/mol), a key hinge residue, and further stabilizes its binding through two π–H interactions with Leu844 (at 4.45 Å and 3.72 Å, with energies of −0.4 and −0.5 kcal/mol, respectively) (Table S5).

Comparing this with other compounds in the series **7**–**27**, a common theme emerges, where most compounds interact with Met793 via hydrogen bonds, a critical feature for anchoring inhibitors in the ATP pocket. For example, compound **8** not only forms a hydrogen bond with Met793 but also donates a hydrogen bond to Cys775 and establishes π–H as well as π–cation interactions with Val726, Lys745, and Cys797. These additional contacts suggest a slightly different binding orientation that may contribute to its docking score (−7.227 kcal/mol), which is quite comparable to that of compound **7** (−7.28 kcal/mol).

Several compounds, such as compounds **10**, **11**, and **12**, emphasize the importance of hydrogen bonds with Met793 (Figure S2), with distances in the range of 2.70–2.76 Å and relatively high interaction energies (up to −4.2 kcal/mol in some cases). Compound **11**, for instance, not only forms a robust hydrogen bond with Met793 but also interacts with Thr854 both as a donor and acceptor and exhibits multiple π–H interactions with Leu718. This multivalent interaction pattern contributes to its favorable docking energy (−7.375 kcal/mol) (Table S5).

Some compounds stand out with even more extensive interaction networks. For example, compound **17** shows a combination of hydrogen bonds with Met793 and an additional π–H interaction with Leu844, resulting in a stronger docking score of –8.057 kcal/mol. Similarly, compound **18** demonstrates a potent hydrogen bond with Met793 (2.95 Å, −3 kcal/mol) along with a supportive π–cation interaction with Lys745, achieving one of the best docking scores (−8.213 kcal/mol).

In summary, while all compounds target the critical hinge region of the EGFR ATP-binding pocket (primarily involving Met793), differences arise in the auxiliary interactions. Variations in hydrogen bonding with residues such as Thr854 and Cys775, along with differences in the extent of π–H and π–cation interactions with hydrophobic residues such as Leu718, Leu844, Val726, and Lys745, lead to notable differences in binding efficiency. Generally, compounds that form a more extensive network of these interactions (e.g., compounds **17**, **18**, and **24**) exhibit stronger predicted binding affinities, as reflected by lower docking scores. This comparative analysis underscores how subtle changes in ligand structure and interaction profiles can significantly influence the overall binding performance against the EGFR target.

#### 2.9.3. Molecular Dynamics (MD) Simulation

Molecular dynamics (MD) simulation is a computational methodology that provides predictions for the surrounding system and protein properties in the binding pocket [44]. The deviation comparison between two overlapping structures can be calculated using the Root Mean Square Deviation (RMSD) in order to validate the accuracy of the model and the existence of the system in the native structure [45]. Because compound **24** exhibited remarkable inhibitory activities on HER2 and EGFR, it was selected to perform MD simulation. The key MD simulation results for compound **24** bound to EGFR and HER2 are compared below with the respective co-crystallized inhibitors—Gefitinib (for EGFR) and Pro-03Q (for HER2)—along with the apo forms of each protein.

##### Root Mean Square Deviation (RMSD)

**Figure 8:**
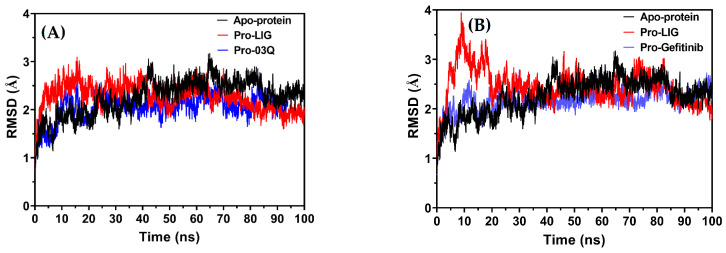
The Root Mean Square Deviation (RMSD) analysis of compound **24** and the co-crystallized ligand (**A**) Pro-03Q with HER2 and (**B**) Gefitinib with EGFR over 100 ns molecular dynamics simulations. The RMSD plot reflects the structural stability and conformational changes in the protein–ligand complexes, where compound **24** exhibits a stable binding mode comparable to the reference co-crystallized ligands in both the HER2 and EGFR systems.

The RMSD for compound **24** (2.268 ± 0.265 Å) in HER2 is moderately higher than the Pro-03Q complex (2.057 ± 0.261 Å), suggesting that compound **24** induces more structural rearrangement. However, it remains comparable to the apo HER2 (2.257 ± 0.37 Å), indicating overall stable dynamics (Figure 8A).

The RMSD for compound **24** (2.457 ± 0.346 Å) in EGFR is slightly higher than that of co-crystallized Gefitinib (2.170 ± 0.222 Å). This indicates a marginally greater overall positional fluctuation in the complex with compound **24** but still within a stable range compared with the apo form (2.257 ± 0.3695 Å) (Figure 8B).

##### Root Mean Square Fluctuation (RMSF)

Molecular dynamics simulations revealed notable differences in residue-level fluctuations (RMSFs) when comparing compound **24**-bound complexes of EGFR and HER2 with their respective co-crystallized ligands (Gefitinib for EGFR and Pro-03Q for HER2). Both EGFR and Her2 adopt a typical kinase bilobed architecture, with an N-terminal lobe (N-lobe) composed mainly of β-strands and an αC helix and a C-terminal lobe (C-lobe) that is predominantly α-helical. These lobes are connected by a flexible hinge region and define the ATP-binding cleft, where small-molecule inhibitors typically reside. Below, we highlight key RMSF findings in the context of important structural elements, such as the glycine-rich loop, αC helix, DFG motif, catalytic loop, and activation loop.

**Figure 9:**
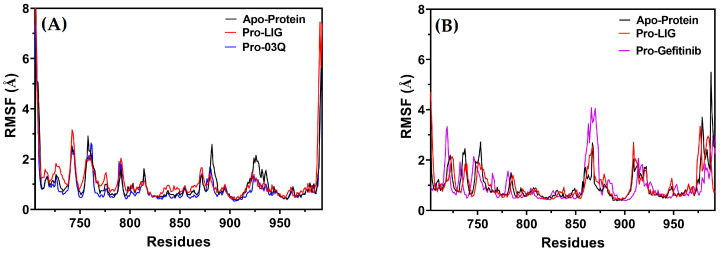
Root Mean Square Fluctuation (RMSF) analysis of backbone Cα atoms for compound **24** and co-crystallized ligands with (**A**) HER2 and (**B**) EGFR. The RMSF profiles reveal the fluctuation patterns across key structural regions, including the glycine-rich loop, αC helix, and activation loop. Compound **24** shows distinct fluctuation patterns compared with the co-crystallized ligands, indicating differential stabilization effects on the kinase domains.

In the Pro-03Q-bound HER2 complex, increased RMSF is observed at residues 708–710, 729–733, and 742–744, with the segment 729–733 overlapping the glycine-rich loop (Leu726–Val734). This loop plays a crucial role in positioning the phosphate groups of ATP or inhibitors and is typically characterized by significant flexibility in its kinase domains. In contrast, for the compound **24**-bound complex, a major increase in RMSFs is noted at residues 760–762, which lie at the start of the αC helix (Pro761–Ala775). Interestingly, this is immediately followed by a decrease in fluctuations in residues 763–768, suggesting that while the N-terminal end of the αC helix remains flexible, the remainder of the helix is more stabilized when bound to compound **24** (Figure 9A).

Further differences are evident in the regions surrounding the αC helix and activation loop. In the Pro-03Q complex, a decrease in RMSFs is observed at residues 758–759, whereas compound **24** appears to extend this dampening effect in 763–768, indicating a distinct pattern of conformational stabilization around the αC helix. Both ligands also influence the residues near the activation loop (Asp863–Val884); however, the specific RMSF shifts differ slightly—with Pro-03Q affecting residues 923–932 and compound **24** influencing residues 923–936—suggesting that each ligand induces unique rearrangements in the C-lobe loops that could impact the kinase’s transition between active and inactive states (Figure 9A).

Additionally, although the RMSF data do not explicitly highlight HER2’s C-terminal tail (Pro999–Leu1009), which is capable of forming short helical segments that interact with the hinge region, the observed fluctuations in the adjacent regions imply that both compound **24** and Pro-03Q induce local adjustments that may propagate toward the tail. These collective observations highlight the differential impact of each ligand on the flexibility of key structural regions in HER2, potentially influencing the overall kinase regulation and inhibitor binding dynamics.

In Gefitinib-bound EGFR, increased fluctuations are observed around residues 716–720, 730–732, and 744–747, which are regions that lie in or near the glycine-rich loop (Leu718–Val726) and the hinge region. This pattern suggests that Gefitinib binding modulates the conformational flexibility of these N-lobe loops, thereby influencing ATP access and inhibitor binding. In contrast, EGFR complexed with compound **24** shows increased RMSFs at residues 724–727, which are also part of the glycine-rich loop but shifted slightly downstream. These differences indicate that compound **24** may affect the stability of these loop residues in a distinct manner compared with Gefitinib, highlighting the hinge region’s role as a key determinant of selectivity and binding affinity (Figure 9B).

Both inhibitors also alter the flexibility of segments that encompass the αC helix (Asn756–Ser768). In the Gefitinib-bound complex, residues 765–767 exhibit increased RMSFs, whereas the region encompassing residues 746–753 in the compound **24**-bound complex displays reduced fluctuations. These variations are consistent with the regulatory function of the αC helix, whose orientation is critical for toggling between the active and inactive states of the kinase.

Furthermore, differences in the dynamics of the C-terminal lobe and tail region are apparent. Gefitinib-bound EGFR demonstrates higher RMSFs in regions 861–873, 883–886, and 920–922, which correlate with parts of the activation loop (Asp855–Val876) and the C-terminal tail. In contrast, compound **24** predominantly affects residues 974–977 and 988–995, which are located further downstream in the C-terminal region. These subtle shifts in the dynamics of the C-lobe may reflect the distinct ways in which each inhibitor stabilizes or distorts the tail segments, ultimately influencing kinase regulation.

As a result, the RMSF value for compound **24** with HER2 (1.1414 ± 1.0204 Å) is higher than that of Pro-03Q (0.894 ± 0.8534 Å). This indicates that certain regions of HER2 exhibit slightly increased fluctuation with compound **24** compared with the co-crystallized ligand (Figure 9A). On the other hand, EGFR with compound **24** shows similar average RMSF (1.035 ± 0.737 Å) to that of Gefitinib (1.028 ± 0.63 Å). Both ligand-bound complexes show comparable flexibility profiles, suggesting that compound **24** stabilizes EGFR similarly to Gefitinib (Figure 9B).

##### Compactness

**Figure 10:**
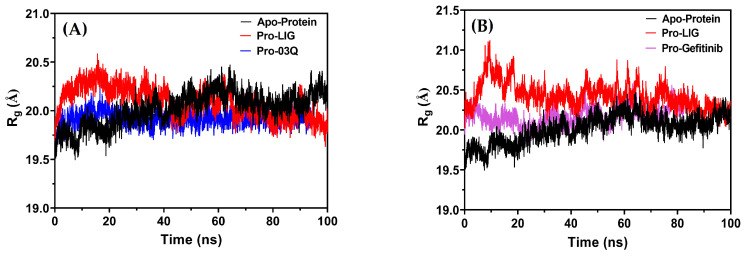
Radius of gyration (Rg) analysis for compound **24** and the co-crystallized ligand in (**A**) HER2 and (**B**) EGFR. The Rg values reflect the compactness and structural stability of the protein–ligand complexes over the 100 ns molecular dynamics simulation. A slight fluctuation in Rg indicates conformational adjustments upon ligand binding, where compound **24** exhibits a more stable and compact structure compared with the co-crystallized ligand in both HER2 and EGFR.

The compactness of the protein is directly proportional to the rate of protein folding that can be followed using the advanced computational method [46]. Two parameters were used to examine the compactness and surface exposure of the protein. The radius of gyration (Rg) is one parameter that demonstrates the distribution of protein atoms around the axis [47]. The other parameter is the Solvent-Accessible Surface Area (SASA), which is the surface area measurement around a protein that is accessible to a solvent in terms of van der Waals interactions with the molecule [48]. Therefore, measurements of Rg and SASA are crucial for analyzing protein stability and interactions with the ligand. The Rg with compound **24** (20.091 ± 0.156 Å) is marginally higher than Pro-03Q (19.92 ± 0.077 Å). Overall, both complexes with HER2 remain in a compact conformation, indicating no major structural unfolding (Figure 10A). On the other hand, the Rg for compound **24** (20.18 ± 0.117 Å) with EGFR is slightly lower than that of Gefitinib (20.43 ± 0.16 Å) but remains close to the apo form (20.016 ± 0.171 Å). This suggests that a compact protein conformation is maintained in the presence of compound **24** (Figure 10B).

**Figure 11:**
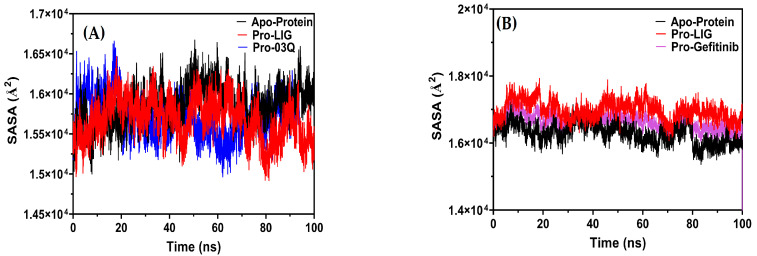
Solvent-Accessible Surface Area (SASA) analysis for compound **24** and co-crystallized ligands with (**A**) HER2 and (**B**) EGFR throughout 100 ns MD simulations. The plot shows the dynamic behavior of the protein–ligand complexes, where compound **24** exhibits reduced the SASA compared with the co-crystallized ligands, indicating tighter binding and potential structural stability in both targets.

In contrast, the SASA of compound **24** (15678.42 ± 254.32 Å^2^) is slightly lower than that of the co-crystallized ligand with Pro-03Q (15701.71 ± 246.486 Å^2^). This indicates a comparable solvent exposure profile, suggesting a stable binding mode for compound **24** with HER2 (Figure 11A). The lower SASA means the ligand is positioned deeper in the pocket; hence, this led to stable conformation. The SASA for compound **24** (16975.64 ± 283.25 Å^2^) is higher than that of Gefitinib (16630.20 ± 249.039 Å^2^), suggesting a slight increase in the solvent exposure of the EGFR–compound **24** complex. This may reflect minor conformational changes in surface residues (Figure 11B). The structural dynamics alterations in HER2 and EGFR upon binding to compound **24** can provide an understanding of its interactions with other molecules and its impact on exploring more investigations toward the targeted therapeutic approaches.

## 3. Materials and Methods

### 3.1. General

Melting points (M.p) (uncorrected) and IR spectra were recorded on a Barnstead 9100 Electrothermal melting apparatus and an FT-IR PerkinElmer spectrometer (PerkinElmer Inc., Waltham, MA, USA), respectively. ^1^H and ^13^C NMR spectra were obtained in deuterated dimethyl sulfoxide (DMSO-*d*_6_) using a Bruker 700 MHz NMR spectrometer and a 176 MHz NMR spectrometer (Bruker, Billerica, MA, USA), respectively, with tetramethylsilane (TMS) employed as an internal standard (chemical shifts in ppm). An Agilent 6320 Ion Trap mass spectrometer was adopted with an ESI ion source (Agilent Technologies, Palo Alto, CA, USA) to record mass spectra. Compounds **5** and **6** were synthesized according to previously reported methods [49].

#### 3.1.1. General Procedure for the Synthesis of Compounds **7**–**27**

A solution of 2-(2,4-dioxo-5,5-diphenylimidazolidin-3-yl)acetohydrazide (**6**) (1.0 mmol, 0.324 g) and a suitable ketone (1.1 mmol) in dry methanol (10 mL) was stirred for 6 h at room temperature. After completion of the reaction, the formed precipitate was recovered using filtration, dried, and recrystallized using the proper solvent.

##### 2-(2,5-Dioxo-4,4-diphenylimidazolidin-1-yl)-*N′*-(1-phenylethylidene)acetohydrazide (**7**)

Yield: 82%; M.p.: 333–335 °C. Mol. formula (Mol. Wt.): C_25_H_22_N_4_O_3_ (426.48); IR (KBr, cm^−1^) ν: 3211 (NH), 1771, 1706 (C=O); ^1^H NMR (700 MHz, DMSO-*d*_6_) δ 10.97, 10.79 (s, 1H), 9.70–9.59 (m, 1H), 7.83, 7.81 (d, *J* = 6.2, 4.8 Hz, 1H), 7.46–7.38 (m, 13H), 7.39 (s, 1H), 4.65, 4.34 (s, 2H), 2.32, 2.29 (s, 3H); ^13^C NMR (176 MHz, DMSO-*d*_6_) δ 174.08, 168.66, 155.59, 149.45, 140.15, 138.27, 129.72, 128.94, 128.92, 128.89, 128.85, 128.68, 128.66, 128.63, 127.50, 126.66, 70.04, 40.56, 14.10; ESI-MS, *m*/*z* (Rel. Int.): 457.23 [M+H, 100%]^+^, 458.23 [M+2, 28%]^+^.

##### 2-(2,5-Dioxo-4,4-diphenylimidazolidin-1-yl)-*N′*-(1-(o-tolyl)ethylidene)acetohydrazide (**8**)

Yield: 73%; M.p.: 232–235 °C. Mol. formula (Mol. Wt.): C_26_H_24_N_4_O_3_ (440.50); IR (KBr, cm^−1^) ν: 3206 (NH), 1773, 1698 (C=O); ^1^H NMR (700 MHz, DMSO-*d*_6_) δ 10.87, 10.72 (s, 1H), 9.66 (d, *J* = 16.5 Hz, 1H), 7.45–7.37 (m, 10H), 7.36 (d, *J* = 7.3 Hz, 1H), 7.27 (t, *J* = 6.9 Hz, 1H), 7.25–7.23 (m, 2H), 4.48, 4.33 (s, 2H), 2.37, 2.32 (s, 3H), 2.24 (s, 3H); ^13^C NMR (176 MHz, DMSO-*d*_6_) δ 174.07, 168.48, 155.56, 152.38, 140.16, 139.66, 135.63, 131.21, 128.92, 128.90, 128.73, 128.68, 128.65, 128.64, 127.50, 126.22, 70.02, 40.43, 20.96, 18.32; ESI-MS, *m*/*z* (Rel. Int.): 441.23 [M+H, 100%]^+^, 442.22 [M+2, 32%]^+^.

##### 2-(2,5-Dioxo-4,4-diphenylimidazolidin-1-yl)-*N′*-(1-(p-tolyl)ethylidene)acetohydrazide (**9**)

Yield: 68%; M.p.: 306–308 °C. Mol. formula (Mol. Wt.): C_26_H_24_N_4_O_3_ (440.50); IR (KBr, cm^−1^) ν: 3192 (NH), 1772, 1696 (C=O); ^1^H NMR (700 MHz, DMSO-*d*_6_) δ 10.91, 10.74 (s, 1H), 9.67 (d, *J* = 9.0 Hz, 1H), 7.74–7.70 (m, 2H), 7.53–7.32 (m, 10H), 7.24, 7.21 (d, *J* = 7.3 Hz, 2H), 4.63, 4.33 (s, 2H), 2.33 (s, 3H), 2.29, 2.25 (s, 3H); ^13^C NMR (176 MHz, DMSO-*d*_6_) δ 174.10, 168.54, 155.61, 149.51, 140.14, 139.36, 135.49, 129.44, 128.92, 128.66, 127.50, 126.59, 70.05, 40.55, 21.29, 14.02; EI-MS, *m*/*z*: 440.5 [M]^+^.

##### *N*′-(1-(4-Chlorophenyl)ethylidene)-2-(2,5-dioxo-4,4-diphenylimidazolidin-1-yl)acetohydrazide (**10**)

Yield: 90%; M.p.: 319–321 °C. Mol. formula (Mol. Wt.): C_25_H_21_ClN_4_O_3_ (460.92); IR (KBr, cm^−1^) ν: 3193 (NH), 1772, 1699 (C=O); ^1^H NMR (700 MHz, DMSO-*d*_6_) δ 11.03, 10.84 (s, 1H), 9.68 (d, *J* = 6.5 Hz, 1H), 7.86, 7.83 (d, *J* = 8.2 Hz, 2H), 7.50, 7.39 (d, *J* = 8.4, 6.8 Hz, 2H), 7.44 (q, *J* = 7.5 Hz, 10H), 4.65, 4.35 (s, 2H), 2.31, 2,27 (s, 3H); ^13^C NMR (176 MHz, DMSO-*d*_6_) δ 174.07, 168.71, 155.57, 148.31, 140.13, 137.10, 134.41, 128.93, 128.83, 128.67, 128.47, 127.49, 70.06, 40.57, 13.98; ESI-MS, *m/z* (Rel. Int.): 459.04 [M-H, 100%]^−^, 461.00 [M+H, 42%]^−^, 462.08 [M+2, 6%]^−^.

##### 2-(2,5-Dioxo-4,4-diphenylimidazolidin-1-yl)-*N′*-(1-(4-fluorophenyl)ethylidene)acetohydrazide (**11**)

Yield: 74%; M.p.: 323–325 °C. Mol. formula (Mol. Wt.): C_25_H_21_FN_4_O_3_ (444.47); IR (KBr, cm^−1^) ν: 3199 (NH), 1700 (C=O); ^1^H NMR (700 MHz, DMSO-*d*_6_) δ 10.97, 10.80 (s, 1H), 9.67 (d, *J* = 8.4 Hz, 1H), 7.89, 7.85 (t, *J* = 8.6 Hz, 2H), 7.42 (m, 10H), 7.27, 7.22 (t, *J* = 8.6 Hz, 2H), 4.64, 4.34 (s, 2H), 2.31, 2.27 (s, 3H); ^13^C NMR (176 MHz, DMSO-*d*_6_) δ 174.05, 168.65, 163.25 (d, *^1^J_C-F_* = 246.33), 155.58, 148.52, 140.11, 134.78 (d, *^4^J_C-F_* = 2.41), 129.05 (d, *^3^J_C-F_* = 8.21), 128.93, 128.66, 127.49, 115.67 (d, *^2^J_C-F_* = 21.53), 70.04, 40.52, 14.13; EI-MS, *m*/*z*: 444.9 [M]^+^.

##### 2-(2,5-Dioxo-4,4-diphenylimidazolidin-1-yl)-*N′*-(1-(4-(trifluoromethyl)phenyl)ethylidene)acetohydrazide (**12**)

Yield: 77%; M.p.: 303–305 °C. Mol. formula (Mol. Wt.): C_26_H_21_F_3_N_4_O_3_ (494.47); IR (KBr, cm^−1^) ν: 3198 (NH), 1699 (C=O); ^1^H NMR (700 MHz, DMSO-*d*_6_) δ 11.13, 10.93 (s, 1H), 9.68 (s, 1H), 8.04, 8.00 (d, *J* = 8.0 Hz, 2H), 7.79, 7.74 (d, *J* = 7.9 Hz, 2H), 7.49–7.21 (m, 10H), 4.66, 4.36 (s, 2H), 2.35, 2.31 (s, 3H); ^13^C NMR (176 MHz, DMSO-*d*_6_) δ 174.07, 168.87, 155.55, 148.02, 142.16, 140.11, 129.61 (q, *^2^J_C-F_* = 31.9), 128.93, 128.68, 127.47, 125.86 (q, *^3^J_C-F_* = 3.6), 125.68, 124.67 (q, *^1^J_C-F_* = 271.8), 70.07, 40.58, 14.10; EI-MS, *m*/*z*: 494.0 [M]^+^.

##### 2-(2,5-Dioxo-4,4-diphenylimidazolidin-1-yl)-*N′*-(1-(4-(trifluoromethoxy)phenyl)ethylidene)acetohydrazide (**13**)

Yield: 85%; M.p.: 261–263 °C. Mol. formula (Mol. Wt.): C_26_H_21_F_3_N_4_O_4_ (510.47); IR (KBr, cm^−1^) ν: 3190 (NH), 1695 (C=O); ^1^H NMR (700 MHz, DMSO-*d*_6_) δ 11.05, 10.88 (s, 1H), 9.68 (d, *J* = 5.8 Hz, 1H), 7.94 (dd, *J* = 24.5, 8.4 Hz, 2H), 7.43 (m, 10H), 7.38 (d, *J* = 8.1 Hz, 2H), 4.65, 4.35 (s, 1H), 2.32, 2.29 (s, 3H); ^13^C NMR (176 MHz, DMSO-*d*_6_) δ 174.07, 168.75, 155.57, 149.36 (d, *^3^J_C-F_* = 18.16), 148.13, 140.12, 137.54, 128.92, 128.71, 127.48, 121.25, 120.01 (q, *^1^J_C-F_* = 256.35), 70.05, 40.57, 14.11; EI-MS, *m*/*z*: 511.2 [M+H]^+^, 511.2 [M+2H]^+^.

##### 2-(2,5-Dioxo-4,4-diphenylimidazolidin-1-yl)-*N′*-(1-(2-methoxyphenyl)ethylidene)acetohydrazide (**14**)

Yield: 91%; M.p.: 185–187 °C. Mol. formula (Mol. Wt.): C_26_H_24_N_4_O_4_ (456.50); IR (KBr, cm^−1^) ν: 3427, 3191 (NH), 1779, 1720, 1669 (C=O); ^1^H NMR (700 MHz, DMSO-*d*_6_) δ 10.87, 10.70 (s, 1H), 9.66, 9.64 (s, 1H), 7.50–7.10 (m, 10H), 7.37 (d, *J* = 7.3 Hz, 2H), 7.08 (d, *J* = 8.2 Hz, 1H), 6.96 (t, *J* = 7.5 Hz, 1H), 4.51, 4.32 (s, 2H), 3.83 (s, 3H), 2.22, 2.19 (s, 3H); ^13^C NMR (176 MHz, DMSO-*d*_6_) δ 174.06, 168.47, 157.62, 155.57, 151.78, 140.17, 130.76, 129.74, 129.21, 128.90, 128.63, 127.50, 120.83, 112.14, 70.02, 56.03, 40.41, 18.20; EI-MS, *m*/*z*: 455.5 [M-H]^+^.

##### 2-(2,5-Dioxo-4,4-diphenylimidazolidin-1-yl)-*N′*-(1-(3-methoxyphenyl)ethylidene)acetohydrazide (**15**)

Yield: 78%; M.p.: 244–246 °C. Mol. formula (Mol. Wt.): C_26_H_24_N_4_O_4_ (456.50); IR (KBr, cm^−1^) ν: 3212 (NH), 1769, 1710, 1680 (C=O); ^1^H NMR (700 MHz, DMSO-*d*_6_) δ 10.94, 10.77 (s, 1H), 9.67, 9.64 (s, 1H), 7.44–7.34 (m, 10H), 7.37 (d, *J* = 6.9 Hz, 1H), 7.33 (s, 1H), 7.32–7.29 (m, 1H), 7.09–6.81 (m, 1H), 4.63, 4.32 (s, 2H), 3.77 (s, 3H), 2.29, 2.25 (s, 3H); ^13^C NMR (176 MHz, DMSO-*d*_6_) δ 174.10, 168.66, 159.75, 155.60, 149.35, 140.16, 139.73, 129.90, 128.91, 128.65, 127.51, 119.18, 115.45, 111.85, 70.04, 55.62, 40.55, 14.26; ESI-MS, *m*/*z* (Rel. Int.): 457.23 [M+H, 100%]^+^, 458.23 [M+2, 32%]^+^.

##### 2-(2,5-Dioxo-4,4-diphenylimidazolidin-1-yl)-*N′*-(1-(4-methoxyphenyl)ethylidene)acetohydrazide (**16**)

Yield: 75%; M.p.: 286–288 °C. Mol. formula (Mol. Wt.): C_26_H_24_N_4_O_4_ (456.50); IR (KBr, cm^−1^) ν: 3181 (NH), 1774, 1681 (C=O); ^1^H NMR (700 MHz, DMSO-*d*_6_) δ 10.86, 10.70 (s, 1H), 9.67, 9.66 (s, 1H), 7.78 (t, *J* = 10.9 Hz, 2H), 7.50–7.32 (m, 10H), 6.97 (dd, *J* = 24.4, 8.3 Hz, 2H), 4.62, 4.33 (s, 2H), 3.79 (s, 3H), 2.28, 2.25 (s, 3H); ^13^C NMR (176 MHz, DMSO-*d*_6_) δ 174.10, 168.43, 160.68, 155.61, 149.30, 140.16, 130.73, 128.94, 128.65, 128.16, 127.50, 114.18, 70.04, 55.70, 40.55, 13.98; EI-MS, *m*/*z*: 457.2 [M+1]^+^ 458.2 [M+2]^+^.

##### *N′*-(1-(3,4-Dimethoxyphenyl)ethylidene)-2-(2,5-dioxo-4,4-diphenylimidazolidin-1-yl)acetohydrazide (**17**)

Yield: 75%; M.p.: 230–232 °C. Mol. formula (Mol. Wt.): C_27_H_26_N_4_O_5_ (486.53); IR (KBr, cm^−1^) ν: 3188 (NH), 1774, 1712, 1680 (C=O); ^1^H NMR (700 MHz, DMSO-*d*_6_) δ 10.83, 10.68 (s, 1H), 9.67, 9.64 (s, 1H), 7.44–7.33 (m, 11H), 7.31 (d, *J* = 9.0 Hz, 1H), 6.98, 6.95 (d, *J* = 8.4 Hz, 1H), 4.63, 4.31 (s, 2H), 3.77 (s, 6H), 2.27, 2.24 (s, 3H); ^13^C NMR (176 MHz, DMSO-*d*_6_) δ 174.17, 168.43, 155.64, 150.59, 149.45, 149.03, 140.17, 130.85, 128.95, 128.90, 128.65, 127.53, 127.48, 120.01, 111.48, 109.39, 70.03, 55.99, 55.96, 40.58, 13.99; EI-MS, *m*/*z*: 485.8 [M]^+^.

##### 2-(2,5-Dioxo-4,4-diphenylimidazolidin-1-yl)-*N′*-(1-(3,4,5-trimethoxyphenyl)ethylidene)acetohydrazide (**18**)

Yield: 59%; M.p.: 265–267 °C. Mol. formula (Mol. Wt.): C_28_H_28_N_4_O_6_ (516.55); IR (KBr, cm^−1^) ν: 3190 (NH), 1773, 1709 (C=O); ^1^H NMR (700 MHz, DMSO-*d*_6_) δ 10.90, 10.74 (s, 1H), 9.69, 9.66 (s, 1H), 7.48–7.33 (m, 10H), 7.09 (s, 2H), 4.65, 4.33 (s, 2H), 3.83 (s, 6H), 3.71, 3.69 (s, 3H), 2.32, 2.28 (s, 3H); ^13^C NMR (176 MHz, DMSO-*d*_6_) δ 174.17, 168.56, 155.63, 153.16, 149.52, 140.16, 139.21, 133.85, 128.90, 128.65, 127.53, 104.29, 70.02, 60.54, 56.48, 56.44, 40.59, 14.32; ESI-MS, *m*/*z* (Rel. Int.): 515.26 [M-H, 100%]^−^, 516.25 [M, 35%]^−^.

##### 2-(2,5-Dioxo-4,4-diphenylimidazolidin-1-yl)-*N′*-(1-(4-nitrophenyl)ethylidene)acetohydrazide (**19**)

Yield: 88%; M.p.: 327–329 °C. Mol. formula (Mol. Wt.): C_25_H_21_N_5_O_5_ (471.47); IR (KBr, cm^−1^) ν: 3362, 3176 (NH), 1816, 1780, 1716 (C=O); ^1^H NMR (700 MHz, DMSO-*d*_6_) δ 11.22, 11.00 (s, 1H), 9.68 (s, 1H), 8.27, 8.21 (d, *J* = 8.4 Hz, 2H), 8.08, 8.05 (d, *J* = 8.2 Hz, 1H), 7.46–7.33 (m, 10H), 4.68, 4.36 (s, 2H), 2.36, 2.32 (s, 3H); ^13^C NMR (176 MHz, DMSO-*d*_6_) δ 174.05, 169.00, 155.53, 147.99, 147.41, 144.36, 140.08, 128.95, 128.69, 127.88, 127.46, 123.96, 70.07, 40.61, 14.09; ESI-MS, *m*/*z* (Rel. Int.): 470.22 [M-H, 100%]^−^, 471.23 [M, 30%]^−^.

##### 2-(2,5-Dioxo-4,4-diphenylimidazolidin-1-yl)-*N′*-(1-(pyridin-2-yl)ethylidene)acetohydrazide (**20**)

Yield: 85%; M.p.: 324–326 °C. Mol. formula (Mol. Wt.): C_24_H_21_N_5_O_3_ (427.46); IR (KBr, cm^−1^) ν: 3206 (NH), 1700 (C=O); ^1^H NMR (700 MHz, DMSO-*d*_6_) δ 11.13, 10.94 (s, 1H), 9.69 (d, *J* = 5.8 Hz, 1H), 8.60 (d, *J* = 4.8 Hz, 1H), 8.14, 8.04 (d, *J* = 8.2 Hz, 1H), 7.86, 7.80 (t, *J* = 7.9 Hz, 1H), 7.48–7.33 (m, 11H), 4.70, 4.37 (s, 2H), 2.39, 2.35 (s, 3H); ^13^C NMR (176 MHz, DMSO-*d*_6_) δ 174.07, 168.79, 155.56, 155.17, 150.17, 148.99, 140.11, 137.09, 128.93, 128.68, 127.48, 124.54, 120.71, 70.06, 40.59, 12.39; EI-MS, *m*/*z*: 428.4 [M+H]^+^.

##### 2-(2,5-Dioxo-4,4-diphenylimidazolidin-1-yl)-*N′*-(1-(pyridin-3-yl)ethylidene)acetohydrazide (**21**)

Yield: 90%; M.p.: 291–293 °C. Mol. formula (Mol. Wt.): C_24_H_21_N_5_O_3_ (427.46); IR (KBr, cm^−1^) ν: 3180 (NH), 1706 (C=O); ^1^H NMR (700 MHz, DMSO-*d*_6_) δ 11.07, 10.90 (s, 1H), 9.67, 9.65 (s, 1H), 8.99, 8.94 (s, 1H), 8.58 (d, *J* = 4.8 Hz, 1H), 8.18, 8.13 (d, *J* = 7.9 Hz, 1H), 7.46–7.32 (m, 11H), 4.65, 4.33 (s, 2H), 2.33, 2.29 (s, 3H); ^13^C NMR (176 MHz, DMSO-*d*_6_) δ 174.06, 168.83, 155.58, 150.39, 147.89, 147.46, 140.14, 134.05, 133.83, 128.92, 128.66, 127.48, 123.88, 70.04, 40.61, 13.95; EI-MS, *m*/*z*: 426.6 [M-H]^+^.

##### 2-(2,5-Dioxo-4,4-diphenylimidazolidin-1-yl)-*N′*-(1-(pyridin-4-yl)ethylidene)acetohydrazide (**22**)

Yield: 90%; M.p.: 324–326 °C. Mol. formula (Mol. Wt.): C_24_H_21_N_5_O_3_ (427.46); IR (KBr, cm^−1^) ν: 3100 (NH), 1779, 1718, 1684 (C=O); ^1^H NMR (700 MHz, DMSO-*d*_6_) δ 11.20, 10.99 (s, 1H), 9.68 (s, 1H), 8.63, 8.59 (d, *J* = 5.2 Hz, 2H), 7.78, 7.73 (d, *J* = 5.2 Hz, 2H), 7.45–7.37 (m, 10H), 4.68, 4.37 (s, 2H), 2.32, 2.28 (s, 3H); ^13^C NMR (176 MHz, DMSO-*d*_6_) δ 174.05, 169.01, 155.54, 150.42, 147.19, 145.20, 140.10, 128.94, 128.68, 127.47, 120.85, 70.06, 40.61, 13.55; EI-MS, *m*/*z*: 426.9 [M]^+^.

##### 2-(2,5-Dioxo-4,4-diphenylimidazolidin-1-yl)-*N′*-(1-(naphthalen-1-yl)ethylidene)acetohydrazide (**23**)

Yield: 70%; M.p.: 245–247 °C. Mol. formula (Mol. Wt.): C_29_H_24_N_4_O_3_ (476.54); IR (KBr, cm^−1^) ν: 3319, 3204 (NH), 1773, 1703, 1684 (C=O); ^1^H NMR (700 MHz, DMSO-*d*_6_) δ 11.05, 10.92 (s, 1H), 9.68, 9.64 (s, 1H), 8.14 (d, *J* = 7.9 Hz, 1H), 8.07–8.00 (m, 1H), 7.98 (t, *J* = 9.5 Hz, 1H), 7.63–7.48 (m, 4H), 7.47–7.27 (m, 10H), 4.49, 4.39 (s, 2H), 2.44 (s, 3H); ^13^C NMR (176 MHz, DMSO-*d*_6_) δ 174.07, 168.56, 155.55, 151.95, 140.14, 137.91, 133.88, 130.46, 129.34, 128.90, 128.65, 127.49, 127.20, 126.51, 125.68, 70.04, 40.45, 19.37; EI-MS, *m*/*z*: 476.4 [M]^+^.

##### 2-(2,5-Dioxo-4,4-diphenylimidazolidin-1-yl)-*N′*-(1-(naphthalen-2-yl)ethylidene)acetohydrazide (**24**)

Yield: 66%; M.p.: 335–337 °C. Mol. formula (Mol. Wt.): C_29_H_24_N_4_O_3_ (476.54); IR (KBr, cm^−1^) ν: 3185 (NH), 1771, 1700 (C=O); ^1^H NMR (700 MHz, DMSO-*d*_6_) δ 11.06, 10.89 (s, 1H), 9.70, 9.63 (s, 1H), 8.31 (s, 1H), 8.11, 8.08 (d, *J* = 8.8 Hz, 1H), 8.03–7.99 (m, 1H), 7.93 (t, *J* = 4.7 Hz, 1H), 7.90 (d, *J* = 8.5 Hz, 1H), 7.55 (dd, *J* = 6.4, 3.2 Hz, 2H), 7.47–7.37 (m, 10H), 4.73, 4.38 (s, 2H), 2.45, 2.41 (s, 3H); ^13^C NMR (176 MHz, DMSO-*d*_6_) δ 174.13, 168.73, 155.63, 149.27, 140.14, 135.59, 133.70, 133.22, 128.94, 128.68, 128.24, 127.95, 127.51, 127.31, 126.69, 123.94, 70.08, 40.68, 27.23, 14.52, 13.88; ESI-MS, *m*/*z* (Rel. Int.): 477.25 [M+H, 100%]^+^, 478.25 [M+2, 35%]^+^.

##### 2-(2,5-Dioxo-4,4-diphenylimidazolidin-1-yl)-*N′*-(1-(furan-2-yl)ethylidene)acetohydrazide (**25**)

Yield: 56%; M.p.: 290–292 °C. Mol. formula (Mol. Wt.): C_23_H_20_N_4_O_4_ (416.44); IR (KBr, cm^−1^) ν: 3199 (NH), 1773, 1711, 1679 (C=O); ^1^H NMR (700 MHz, DMSO-*d*_6_) δ 10.92, 10.73 (s, 1H), 9.65–9.62 (m, 1H), 7.79, 7.77 (s, 1H), 7.54–7.14 (m, 10H), 6.96, 6.93 (s, 1H), 6.59 (s, 1H), 4.55, 4.31 (s, 2H), 2.22, 2.19 (s, 3H); ^13^C NMR (176 MHz, DMSO-*d*_6_) δ 174.06, 168.38, 155.55, 152.07, 144.88, 141.79, 140.14, 128.92, 128.65, 127.49, 112.45, 111.33, 70.04, 40.48, 13.65; ESI-MS, *m*/*z* (Rel. Int.): 417.17 [M+H, 100%]^+^.

##### 2-(2,5-Dioxo-4,4-diphenylimidazolidin-1-yl)-*N′*-(1-(thiophen-2-yl)ethylidene)acetohydrazide (**26**)

Yield: 74%; M.p.: 315–317 °C. Mol. formula (Mol. Wt.): C_23_H_20_N_4_O_3_S (432.50); IR (KBr, cm^−1^) ν: 3195 (NH), 1772, 1706, 1675 (C=O); ^1^H NMR (700 MHz, DMSO-*d*_6_) δ 10.97, 10.74 (s, 1H), 9.67 (s, 1H), 7.59, 7.57 (d, *J* = 5.0 Hz, 1H), 7.48, 7.47 (d, *J* = 3.9 Hz, 1H), 7.44–7.34 (m, 10H), 7.08 (t, *J* = 4.4 Hz, 1H), 4.52, 4.30 (s, 2H), 2.32, 2.29 (s, 3H); ^13^C NMR (176 MHz, DMSO-*d*_6_) δ 174.07, 168.07, 155.53, 146.17, 143.56, 140.11, 129.40, 128.92, 128.66, 128.19, 128.00, 127.49, 70.07, 40.48, 14.53; IR (KBr, cm^−1^) ν: 3195 (NH), 1706, 1675 (C=O); EI-MS, *m*/*z*: 432.1 [M]^+^.

##### *N′*-(1-(5-Chlorothiophen-2-yl)ethylidene)-2-(2,5-dioxo-4,4-diphenylimidazolidin-1-yl)acetohydrazide (**27**)

Yield: 47%; M.p.: 303–305 °C. Mol. formula (Mol. Wt.): C_23_H_19_ClN_4_O_3_S (466.94); IR (KBr, cm^−1^) ν: 3187 (NH), 1769, 1702 (C=O); ^1^H NMR (700 MHz, DMSO-*d*_6_) δ 11.06, 10.84 (s, 1H), 9.67 (s, 1H), 7.44–7.35 (m, 10H), 7.37–7.30 (m, 1H), 7.16–7.03 (m, 1H), 4.51, 4.30 (s, 2H), 2.28, 2.25 (s, 3H); ^13^C NMR (176 MHz, DMSO-*d*_6_) δ 174.02, 168.16, 155.50, 145.45, 142.63, 140.09, 130.90, 128.93, 128.67, 128.02, 127.83, 127.47, 70.06, 40.45, 13.62; ESI-MS, *m/z* (Rel. Int.): 465.14 [M-H, 100%]^−^, 467.14 [M+H, 42%]^−^.

### 3.2. Biological Activity

#### 3.2.1. In Vitro Cytotoxicity Assay

The investigated molecules were assessed for their potential antitumor activity against a panel of human cell lines: colorectal carcinoma (HCT-116), hepatocellular carcinoma (HepG-2), mammary gland breast cancer (MCF-7), and normal human fibroblasts (WI-38). The evaluation was conducted using a 3-(4,5-dimethylthiazol-2-yl)-2,5-diphenyltetrazolium bromide (MTT) assay, as described by the authors of [50,51]. The cell lines were obtained from ATCC. For comparative purposes, standard drugs, such as doxorubicin and erlotinib, were employed.

#### 3.2.2. In Vitro Kinase Inhibition Assay

EGFR and HER2 kinase inhibitory activities were determined based on the manufacturer’s guidelines (EGFR Kinase Assay Kit Catalog no. 40321 and HER2 Kinase Assay Kit Catalog no. 40721, BPS Bioscience, San Diego, CA). In the in vitro luminescent EGFR tyrosine kinase assay and HER2 tyrosine kinase assay using Kinase-Glo Max as a detection reagent, the light emission demonstrated a positive connection with the ADP amount and kinase activity [52,53].

#### 3.2.3. Apoptosis Assay

Apoptosis of the MCF-7 cancer cell line was measured using the Human MCL1eia kit (ELISA Kit) for the MCL-1 enzyme assay and the Invitrogen Zymed^®^ Bcl-2 ELISA Kit for the BCL-2 enzyme assay. The ROBONIK P2000 ELISA READER was used for analysis [54].

#### 3.2.4. Cell Cycle Analysis

Cancer cell line MCF-7 was stained with ab139418_Propidium Iodide (DNA stain) and analyzed using a FACSCalibur flow cytometer [55].

### 3.3. Molecular Docking Methodology of the Selected Compound

#### 3.3.1. Protein Selection and Preparation

The crystal structures of EGFR and HER2 were sourced from the RCSB Protein Data Bank using the X-ray structure codes 2ITY and 3PP0, respectively. These structures illustrate the complexation of EGFR with the inhibitor IRE and HER2 with the inhibitor 03Q [56]. Following downloading, the protein structures underwent corrections to address any discrepancies and were then 3D protonated and subsequently energy minimized using the MOE 2015.1 suite.

#### 3.3.2. Compound and Reference Inhibitor Preparation

The chemical structure of the selected compound, referred to as “**24**”, was sketched using MOE software. Its energy was minimized and read within the MOE environment for docking. As a benchmark for docking validation, the co-crystallized inhibitors IRE (for EGFR) and 03Q (for HER2) were prepared. The protein was re-prepared, and the co-crystallized ligands were redocked using the standard docking protocol, ensuring their readiness for subsequent docking processes [57].

#### 3.3.3. Docking Process

The docking simulations utilized MOE 2015.1 software, wherein the prepared protein acted as the receptor, and the previously arranged database represented the ligand. As previously mentioned, the process was directed using the default MOE settings [58].

Following docking, the optimal poses of compound “**24**” and the ligands (IRE and 03Q) were determined. The selection criteria revolved around scoring, orientation within the receptor pocket, resemblance to the co-crystallized ligand, and interactions with the adjacent amino acids [59].

#### 3.3.4. MD Simulation Studies

Protein–ligand complexes were generated from the most favorable docking poses [60] and prepared using CHARMM-GUI [61], which produced all the necessary configuration files. Ligand parameters for compound **24** were assigned using the CHARMM General Force Field (CGenFF) web-based tool (https://charmm-gui.org/?doc=input/solution, accessed on 7 March 2025) [61,62,63]. The complexes were then solvated with the TIP3P water model by embedding them in a 12 Å octahedral simulation box to ensure adequate water coverage and neutralized with sodium (Na⁺) and chloride (Cl^−^) ions to achieve a final ionic strength of 0.15 M. Additionally, an alternative solvation approach using a 10.0 Å periodic dodecahedron water box was employed to mimic physiological conditions, providing further validation of the simulation setup [61,62,63].

The simulation protocol began with a three-stage process designed to stabilize the systems prior to production. First, a 10,000-step energy minimization was performed to relieve any steric clashes. This was followed by a 1,000,000-step pre-equilibration phase to prepare the system for further equilibration. Next, a 125,000-step equilibration run (125 ps) under the canonical ensemble (NVT) at 310 K was carried out, complementing an initial 200 ps equilibration under both NPT and NVT conditions. These steps ensured that the systems were well equilibrated before proceeding to the production phase.

Production molecular dynamics simulations were conducted using NAMD 3.0 (multicore) in conjunction with the AMBER force field. The simulations were run for 100 ns with a 2 fs time step under NPT conditions (1 atm, 310 K), with the temperature and pressure maintained using coupling constants of 1 ps and 5 ps, respectively. The 100 ns duration was selected based on the observation of RMSD convergence by this time point, although it is acknowledged that extending the simulation could reveal further structural changes [64]. Trajectories were visualized and analyzed with the VMD package [65], and key parameters—including the Root Mean Square Deviation (RMSD), Root Mean Square Fluctuation (RMSF), and radius of gyration (Rg)—were computed to evaluate the binding stability of compound **24** with EGFR and HER2 [66].

### 3.4. ADMET Prediction

The drug-likeness properties of the candidate compounds were rigorously assessed based on a suite of physicochemical parameters, including their solubility, molecular dimensions, polarity, lipophilicity, saturation, and molecular flexibility [67]. To visually represent and assess these properties, a bioavailability radar was generated utilizing the ADMETlab 2.0 platform available at https://admetmesh.scbdd.com/service/screening/index https://admetmesh.scbdd.com/service/screening/ca, accessed on 27 August 2024. Concurrently, the same tool was leveraged to anticipate the potential toxicological profiles of the ligands, as outlined in the study by Xiong et al. [68].

## 4. Conclusions

This study reported the synthesis, characterization, and biological assessment of Schiff’s base derivatives **7**–**27** based on the diphenylimidazolidine-2,4-dione scaffold. The antitumor activities of the synthesized compounds were tested in vitro counteracting the malignant cell lines HCT-116, HepG-2, and MCF-7. In contrast to doxorubicin (IC_50_ = 4.17–5.23 µM) and erlotinib (IC_50_ = 5.18–8.53 µM), derivatives **10**, **13**, **21**, and **24** demonstrated the most active anticancer activity (IC_50_ = 4.92–20.11 µM) against the selected cell lines. Compounds **10**, **13,** and **21** were further investigated and displayed significant EGFR suppressive activity, with IC_50_ = 0.67, 0.61, and 1.61 µM, respectively, as opposed to erlotinib (IC_50_ = 0.05 µM). Furthermore, HER2 tyrosine kinase was powerfully inhibited by the derivatives **10**, **13**, and **21**, with IC_50_ = 0.71, 0.28, 1.17, and 0.04 µM, respectively, compared with lapatinib (IC_50_ = 0.03 µM). Indeed, compound **24** had the highest EGFR and HER2 inhibitory activities (IC_50_ = 0.07 and 0.04 µM, respectively). The apoptosis assay displayed the role of compound **24** in reducing the levels of BCL-2 and MCL-1, which led to apoptosis of the cancer cells. In addition to the cell cycle arrest, which appeared at the G2/M phase, once exposed to derivative **24**, molecular docking and molecular dynamics simulation showed that compound **24** presented good fitting and suitable interactions with the putative binding residues in EGFR and HER2. Therefore, the examined derivatives are considered promising agents that require further optimization and development to obtain highly potent and selective anticancer agents.

## Figures and Tables

**Table 1:** Structures of compounds **7**–**27** and their cytotoxic activities (IC_50_) against human cancer cell lines.

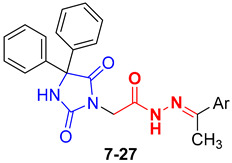				
Compound	Ar	IC_50_ (μM) ^a^		
		HCT-116	HePG-2	MCF-7
**7**	Ph	47.46 ± 2.8	55.81 ± 3.3	38.30 ± 2.2
**8**	2-CH3-Ph	37.61 ± 2.3	25.43 ± 1.8	34.06 ± 2.0
**9**	4-CH_3_-Ph	71.23 ± 3.8	29.73 ± 2.1	69.01 ± 3.9
**10**	4-Cl-Ph	17.90 ± 1.4	10.69 ± 0.9	11.18 ± 0.9
**11**	4-F-Ph	28.33 ± 2.1	21.06 ± 1.8	14.25 ± 1.2
**12**	4-CF_3_-Ph	52.48 ± 3.0	47.71 ± 2.7	45.83 ± 2.4
**13**	4-OCF_3_-Ph	20.11 ± 1.7	13.94 ± 1.2	9.58 ± 0.8
**14**	2-OCH_3_-Ph	74.03 ± 3.9	63.27 ± 3.8	59.14 ± 3.4
**15**	3-OCH_3_-Ph	53.85 ± 3.1	45.12 ± 2.5	55.33 ± 3.2
**16**	4-OCH_3_-Ph	36.52 ± 2.4	27.28 ± 2.0	35.82 ± 2.1
**17**	3,4-Di-OCH_3_-Ph	26.75 ± 2.0	19.63 ± 1.7	15.71 ± 1.3
**18**	3,4,5-Tri-OCH_3_-Ph	39.63 ± 2.4	37.24 ± 2.3	26.02 ± 1.6
**19**	4-NO_2_-Ph	31.27 ± 2.1	19.90 ± 1.8	20.42 ± 1.5
**20**	2-Pyridyl	46.72 ± 2.7	49.10 ± 2.8	32.39 ± 1.9
**21**	3-Pyridyl	19.25 ± 1.5	11.50 ± 1.0	8.61 ± 0.6
**22**	4-Pyridyl	25.96 ± 1.9	17.18 ± 1.6	13.11 ± 1.1
**23**	1-Naphthyl	34.39 ± 2.3	31.82 ± 2.1	28.13 ± 1.7
**24**	2-Naphthyl	12.83 ± 0.9	9.07 ± 0.8	4.92 ± 0.3
**25**	2-Furyl	49.94 ± 2.9	34.56 ± 2.1	47.28 ± 2.6
**26**	2-Thiophenyl	87.50 ± 4.6	32.26 ± 2.2	76.83 ± 4.2
**27**	5-Cl-2-thiophenyl	33.18 ± 2.2	16.84 ± 1.6	19.72 ± 1.5
Doxorubicin	-	5.23 ± 0.3	4.50 ± 0.2	4.17 ± 0.2
Erlotinib	-	8.53 ± 0.5	7.90 ± 0.6	5.18 ± 0.4

^a^ The IC_50_ value is the concentration required to produce 50% inhibition of cancer cell proliferation. Data are illustrated as the mean ± SD from the dose–response curves of three independent experiments. IC_50_ (μM): 1–10 (very strong), 11–20 (strong), 21–50 (moderate), 51–100 (weak), 100–200 (very weak), and above 200 (inactive).

**Table 2:** Cytotoxic activities of compounds **10**, **13**, **21**, and **24** and doxorubicin and erlotinib against normal fibroblast WI-38 and selectivity index (SI) values according to the selected cancer cell lines.

Comp.	WI-38	MCF-7	*SI	HCT-116	SI	HePG-2	SI
**10**	54.21	11.18	4.84	17.9	3.02	10.69	5.07
**13**	45.39	9.58	4.73	20.11	2.25	13.94	3.25
**21**	60.79	8.61	7.06	19.25	3.15	11.5	5.28
**24**	39.52	4.92	8.03	12.83	3.08	9.07	4.35
Doxorubicin	6.72	4.17	1.61	5.23	1.28	4.5	1.49
Erlotinib	46.52	5.18	8.98	8.53	5.45	7.9	5.88

*SI: selectivity index.

**Table 3:** Summary of in vitro evaluation of compounds **10, 13, 21**, and **24**.

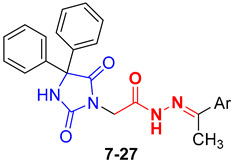			
Compound	Ar	Kinase Inhibition IC_50_ (µM)	
		EGFR	HER2
**10**	4-ChloroPh	0.67 ± 0.03	0.71 ± 0.02
**13**	4-TrifluoromethoxyPh	0.61 ± 0.02	0.28 ± 0.01
**21**	3-Pyridyl	1.61 ± 0.07	1.17 ± 0.04
**24**	2-Naphthyl	0.07 ± 0.004	0.04 ± 0.001
Erlotinib	-	0.05 ± 0.002	nt
Lapatinib	-	nt	0.03 ± 0.001

nt: not tested.

**Table 4:** Cell cycle analysis with 2 µM and 10 µM of compound **24** and the control drug, erlotinib.

Concentrations	%G0–G1	%S	%G2/M	Comment
24 at 2 µM	52.12	22.45	25.43	Cell cycle arrest at G2/M
24 at 10 µM	50.61	15.93	33.46	-
Erlotinib at 2 µM	46.12	22.83	31.05	Cell cycle arrest at G2/M
Erlotinib at 10 µM	35.89	15.72	48.39	-
Cont. MCF-7	63.19	28.99	7.82	-

**Table 5:** Molecular docking interactions of compound **24** with HER2 kinase domain (PDB code: 3PP0) and EGFR kinase domain (PDB code: 2ITY). The table delineates the specific interactions between the ligand atoms and both HER2 and EGFR residues separately, including the type of interaction, distance, and estimated binding energy.

Compound	Ligand	Receptor	Interaction	Distance (Å)	E (kcal/mol)	S (kcal/mol)
The HER2 kinase domain (PDB code: 3PP0)						
Comp. **24**	N 26	O Leu 726 (A)	H-donor	2.85	−1.9	−8.78
	C 29	O Arg849 (A)	H-donor	3.44	−0.7	
	N 35	N Asp863 (A)	H-acceptor	3.3	−1.1	
	O 58	CA Asn850 (A)	H-acceptor	3.56	−0.3	
	6-ring	N Cys805 (A)	pi-H	3.47	−0.5	
	6-ring	CD2 Leu852 (A)	pi-H	3.76	−0.3	
	6-ring	CB Phe864 (A)	pi-H	4.94	−0.3	
	6-ring	CD2 Phe864 (A)	pi-H	4.27	−0.5	
03Q	O1 1	OD2 Asp863 (A)	H-donor	3.03	−2	−10.68
	C12 23	O Gln799 (A)	H-donor	3.27	−0.4	
	CL32 50	O Leu796 (A)	H-donor	3.21	−0.3	
	N11 22	N Met801 (A)	H-acceptor	2.9	−5.5	
	N18 32	CA Asp863 (A)	H-acceptor	3.19	−1.3	
	6-ring	CD1 Leu785 (A)	pi-H	4.3	−0.4	
	6-ring	CD1 Leu800 (A)	pi-H	4.7	−0.4	
	6-ring	CD1 Leu852 (A)	pi-H	3.42	−0.4	
	6-ring	CD2 Phe864 (A)	pi-H	3.83	−0.3	
The EGFR kinase domain (PDB code: 2ITY)						
Comp. **24**	O 58	N Met793 (A)	H-acceptor	3.14	−2.3	−6.71
	6-ring	N Asp855 (A)	pi-H	4.32	−0.5	
IRE	C2 20	O Gln791 (A)	H-donor	3.42	−0.5	−6.53
	N3 19	N Met793 (A)	H-acceptor	2.67	−4.7	
	6-ring	CD1 Leu718 (A)	pi-H	4.17	−0.5	
	6-ring	CD Lys745 (A)	pi-H	4.31	−0.5	
	6-ring	CD1 Leu792 (A)	pi-H	4.97	−0.3	

## Data Availability

The original contributions presented in this study are included in the article/Supplementary Material. Further inquiries can be directed to the corresponding authors.

## Supplementary Materials

The following supporting information can be downloaded at https://www.mdpi.com/article/10.3390/ph18040496/s1: File S1. ^1^H NMR, ^13^C NMR, FT-IR and mass spectra of **10, 13, 16, 18, 22** and **24**, physicochemical characteristics of the investigated compounds, pharmacokinetics properties of the synthesized compounds, metabolic probabilities of being enzyme substrates or inhibitors, molecular docking interactions of **7**–**27** with HER2, and molecular docking interactions of **7**–**27** with EGFR.
