# Supplementary material for: Synthesis, Antitumor Activities, and Apoptosis-Inducing Activities of Schiff’s Bases Incorporating Imidazolidine-2,4-dione Scaffold: Molecular Docking Studies and Enzymatic Inhibition Activities
Source: Pharmaceuticals (Basel). 2025 Mar 28;18(4):496. doi: 10.3390/ph18040496 (PMC12030650; doi:10.3390/ph18040496)

Supplementary Materials

Design, Synthesis, Antitumor and Enzymatic Inhibition Activities of Schiff's Bases Incorporating Imidazolidine-2,4-dione Scaffold: Molecular Docking Studies

Fhdah S. Alanazi [1], Hamad M. Alkahtani [1], Alaa A.-M. Abdel-Aziz[1], Adel S. El-Azab[1], Hanadi H. Asiri[1], Ahmed H. Bakheit[1], Fatmah A. Al-Omary [1]

[1] *Department of Pharmaceutical Chemistry, Collage of Pharmacy, King Saud University, P.O. Box 2457, Riyadh 11451, Saudi Arabia*

Content

- ^{1}H-NMR, ^{13}C-NMR, Mass and FFT-IR spectra of compound 10
- ^{1}H-NMR, ^{13}C-NMR, Mass and FFT-IR spectra of compound 13
- ^{1}H-NMR, ^{13}C-NMR, Mass and FFT-IR spectra of compound 16
- ^{1}H-NMR, ^{13}C-NMR, Mass and FFT-IR spectra of compound 18
- ^{1}H-NMR, ^{13}C-NMR, Mass and FFT-IR spectra of compound 22
- ^{1}H-NMR, ^{13}C-NMR, Mass and FFT-IR spectra of compound 24
- Table S1. Overview of the Physicochemical Characteristics of the Investigated Compounds.
- Table S2: The Pharmacokinetics Properties of the Synthesized Compounds.
- Table S3: Overview of the Metabolic Probabilities of being enzyme substrate or inhibitor for the Synthesized Compounds.
- Table S4: Molecular docking interactions of compounds 7–27 with the Her2. The table details the specific ligand–receptor contacts, including hydrogen bond donors/acceptors and π-interactions, along with their corresponding distances (Å) and interaction energies (E and S in kcal/mol), highlighting the key binding features that contribute to the compounds' overall affinity for the Her2 target.“
- Figure S1: Molecular docking interactions of compounds **7–27** with HER2
- Figure S2: Molecular docking interactions of compounds **7–27** with EGFR

N'-(1-(4-Chlorophenyl)ethylidene)-2-(2,5-dioxo-4,4-diphenylimidazolidin-1-yl)acetohydrazide (10):

^{1}H-NMR spectra of compound 10

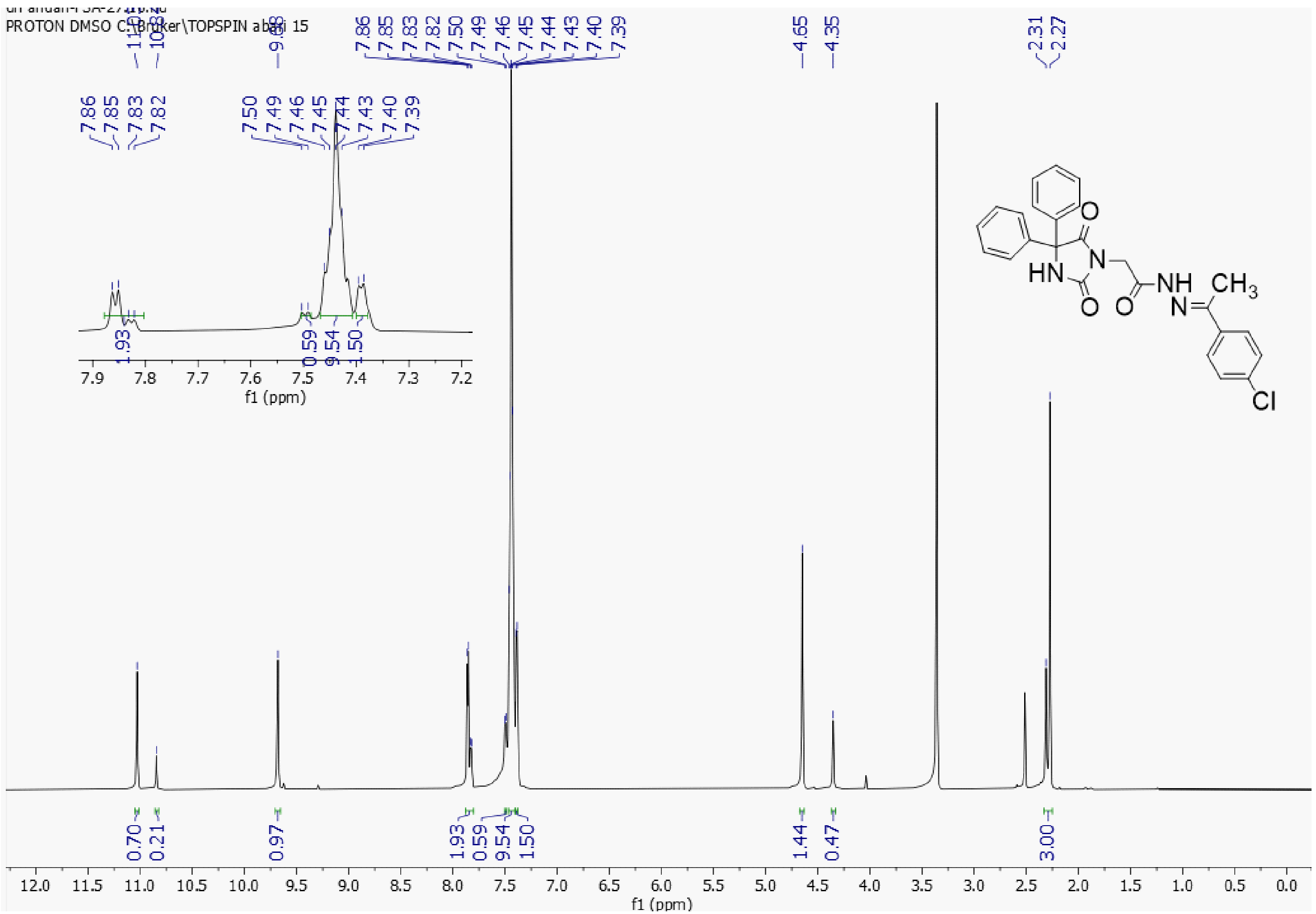

^{13}C-NMR spectra of compound 10

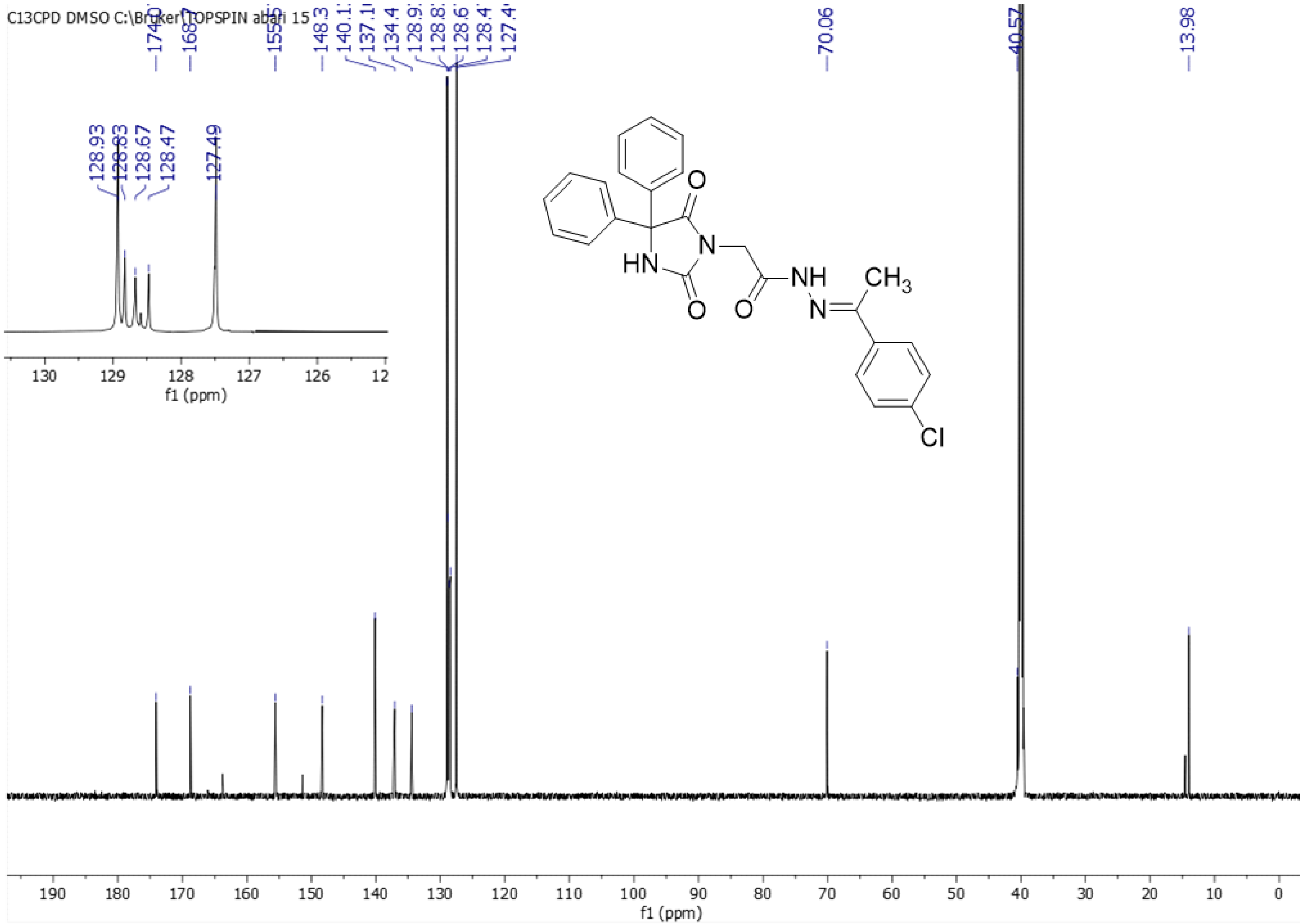

Mass spectra of compound 10

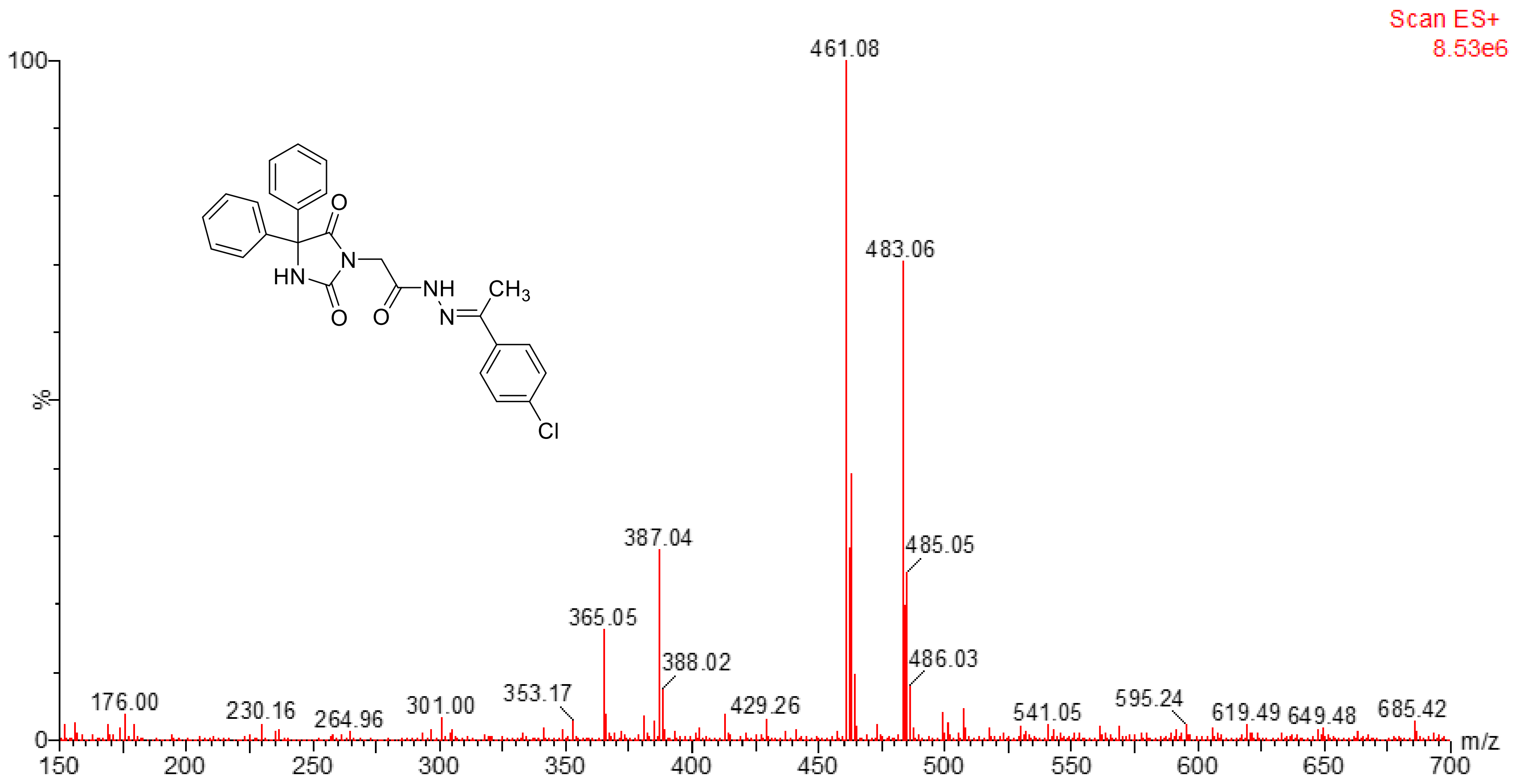

FTIR spectra of compound 10

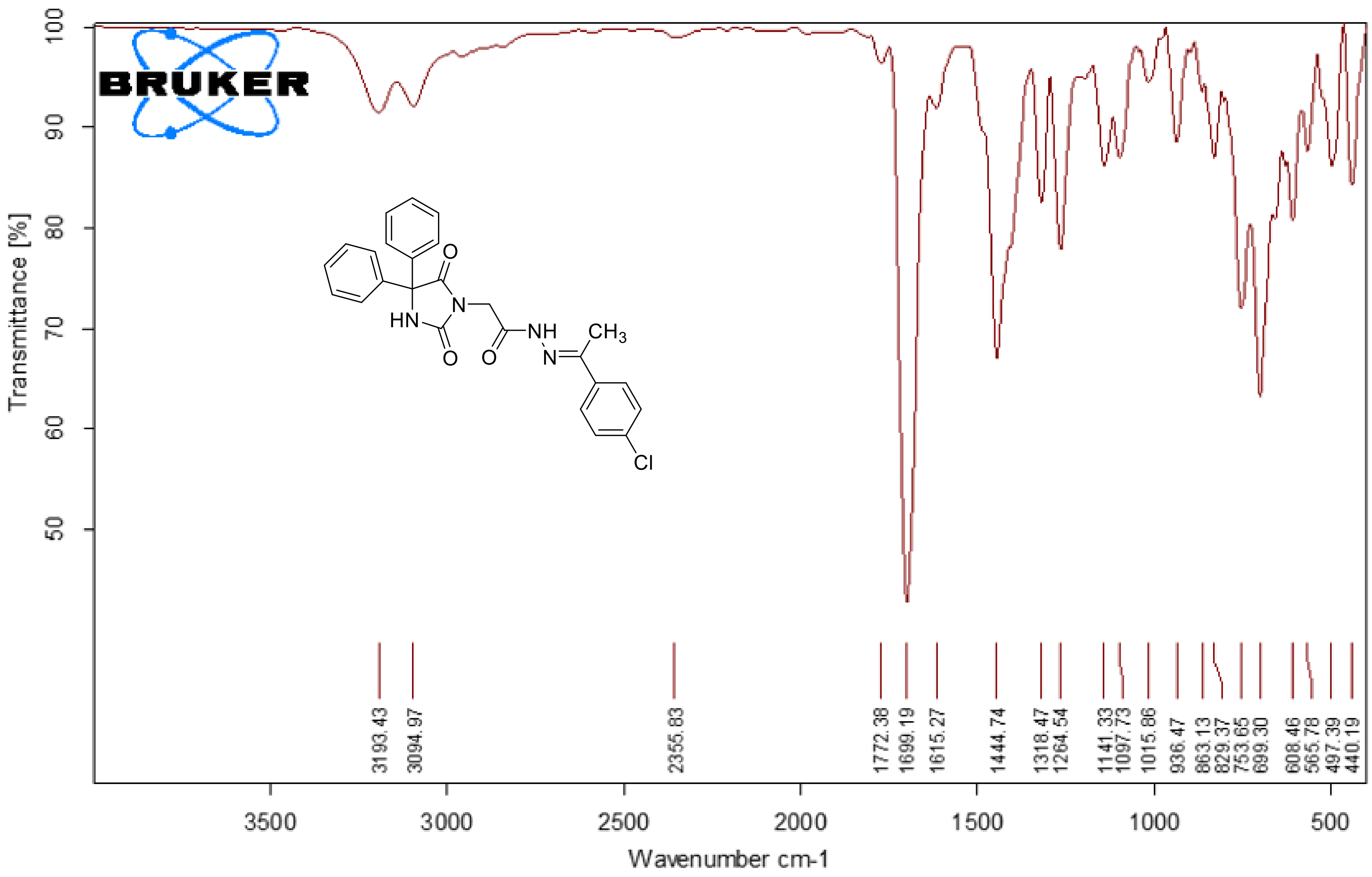

2-(2,5-Dioxo-4,4-diphenylimidazolidin-1-yl)-N′-(1-(4 (trifluoromethoxy)phenyl)ethylidene)acetohydrazide (13):

^{1}H-NMR spectra of compound 13

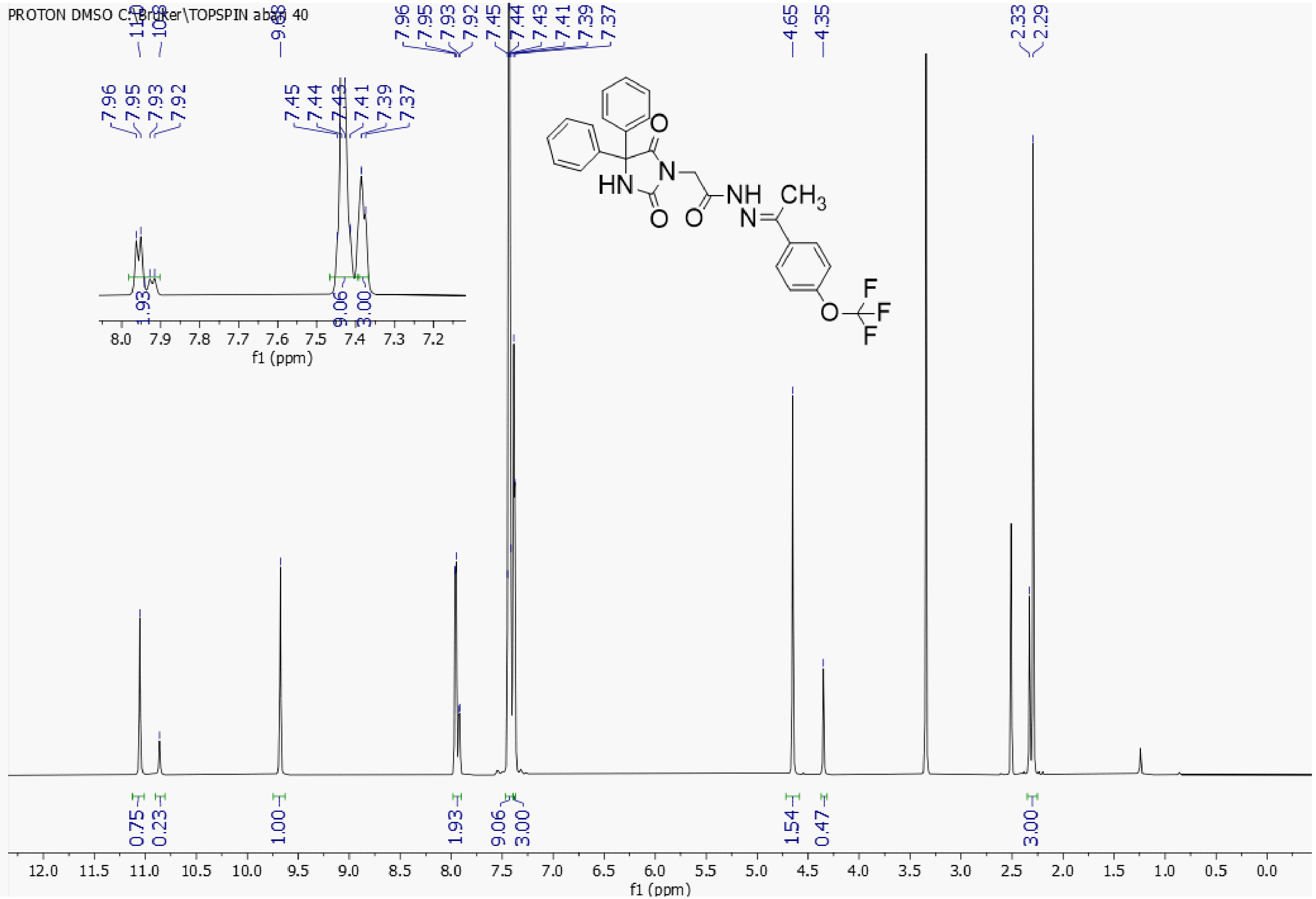

^{13}C-NMR spectra of compound 13

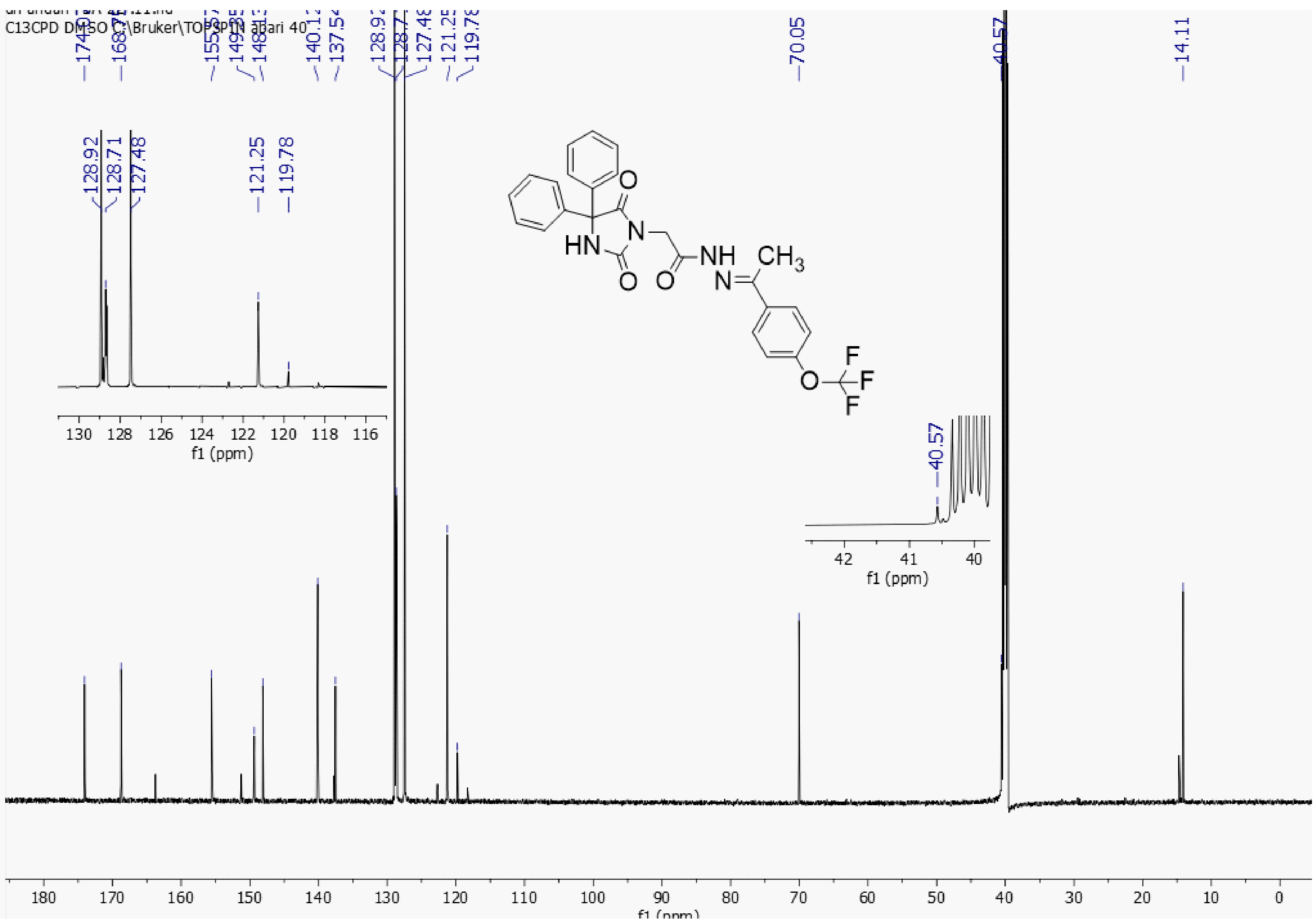

Massa spectra of compound 13

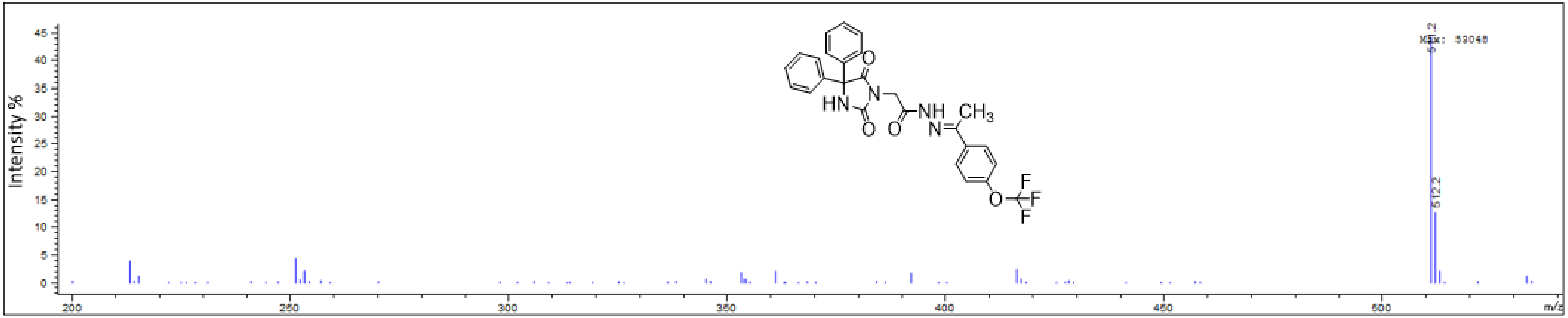

FTIR spectra of compound 13

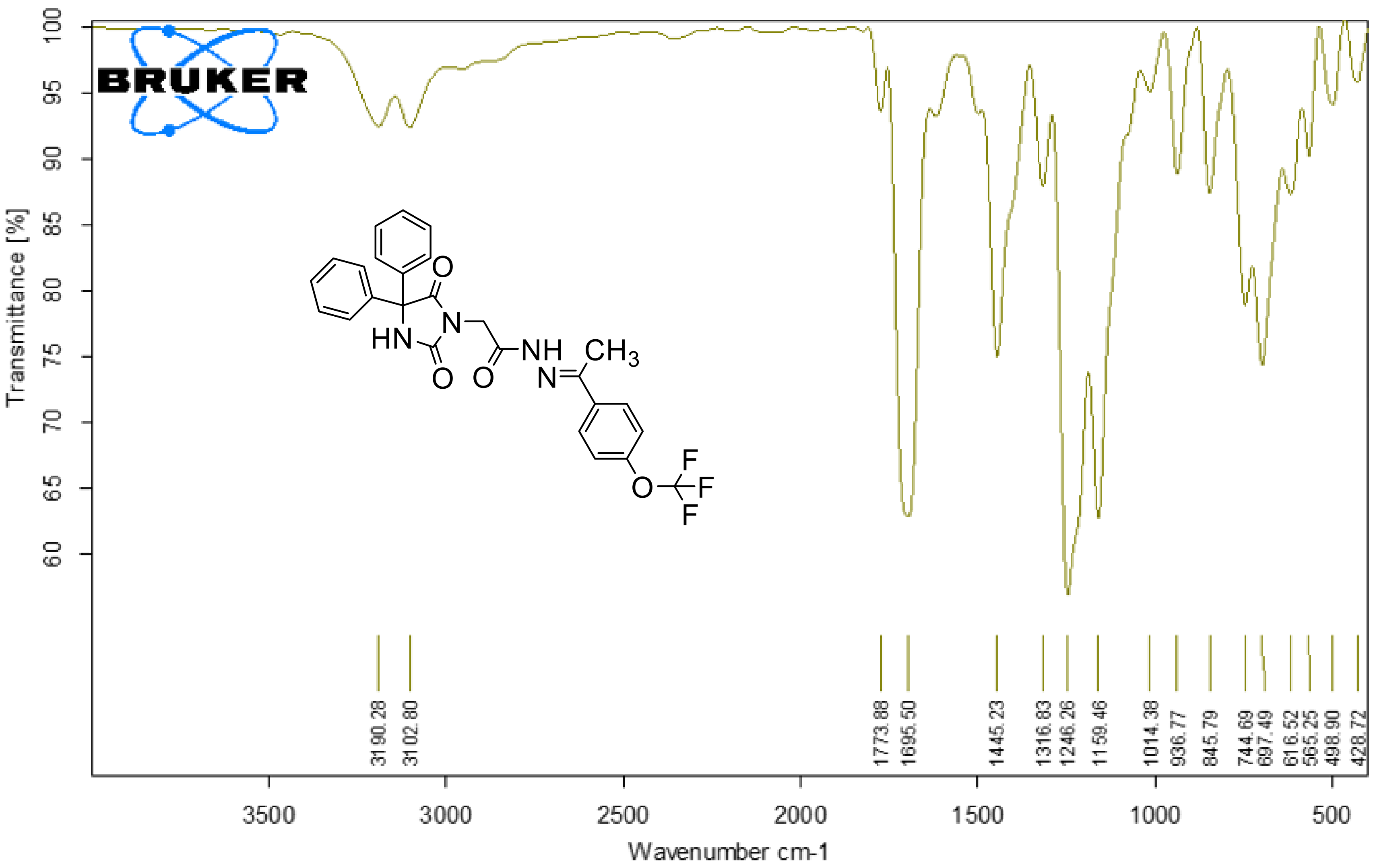

2-(2,5-Dioxo-4,4-diphenylimidazolidin-1-yl)-*N′*-(1-(4-methoxyphenyl)ethylidene)acetohydrazide (16):

^{1}H-NMR spectra of compound 16

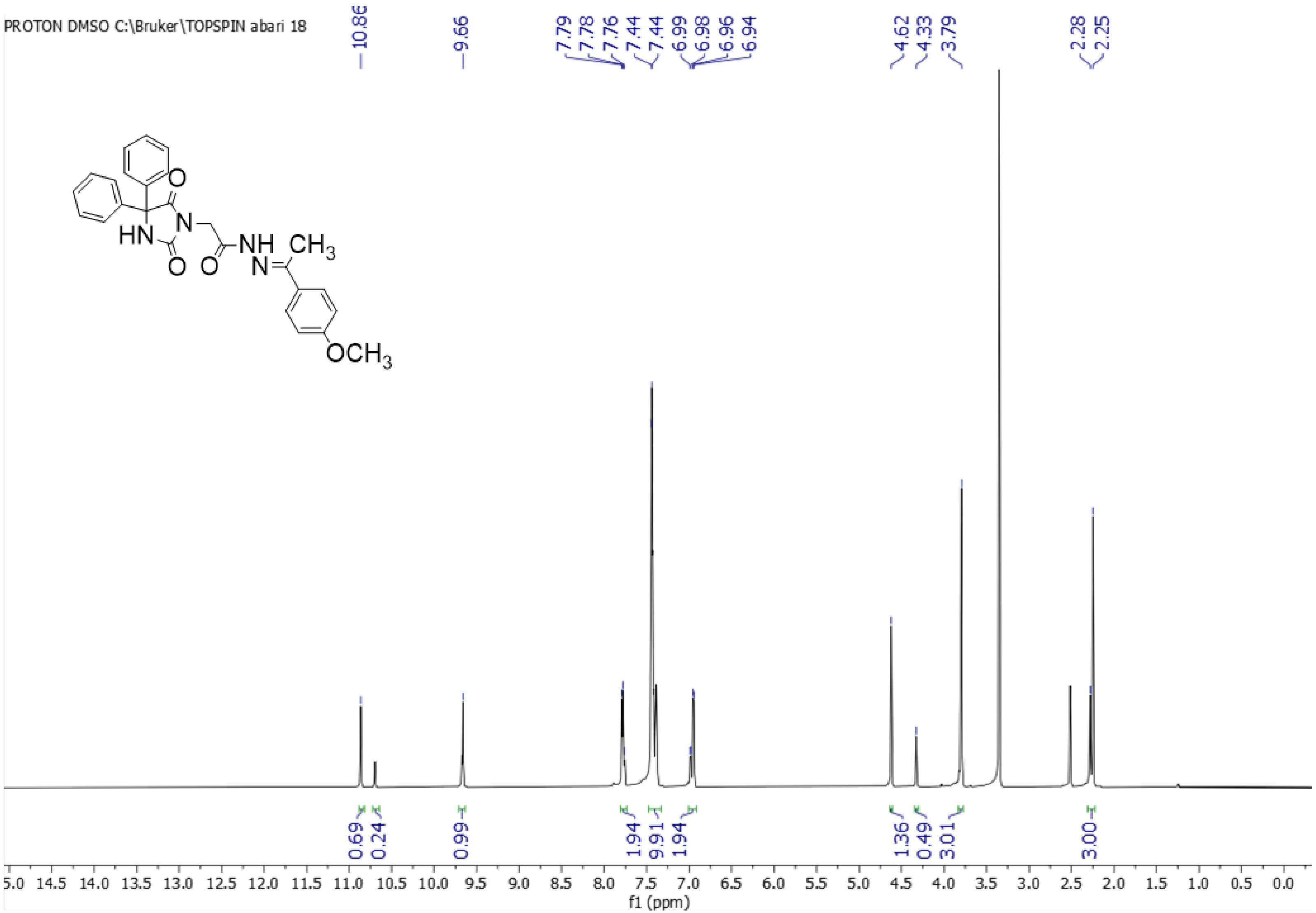

^{13}C-NMR spectra of compound 16

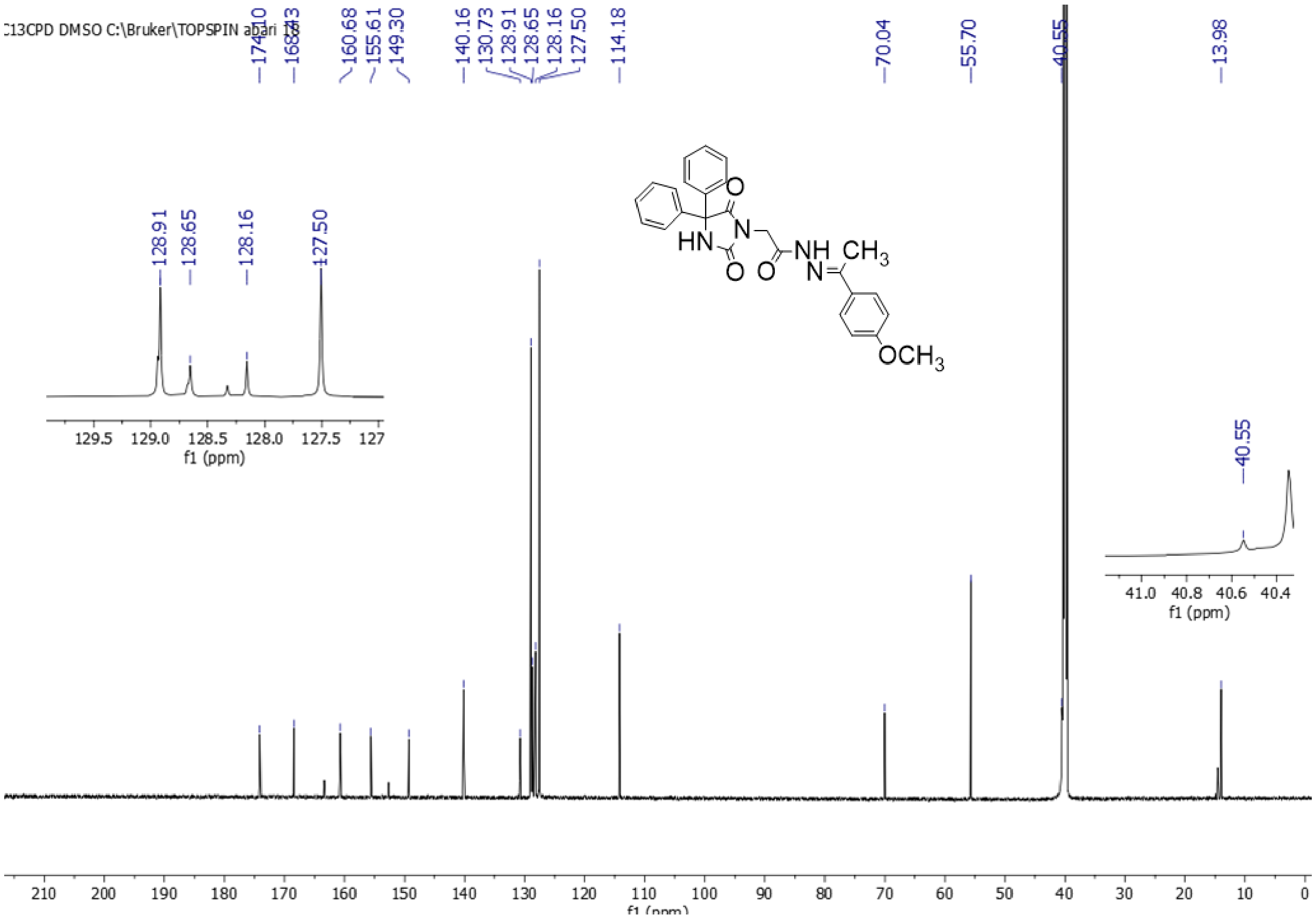

Mass spectra of compound 16

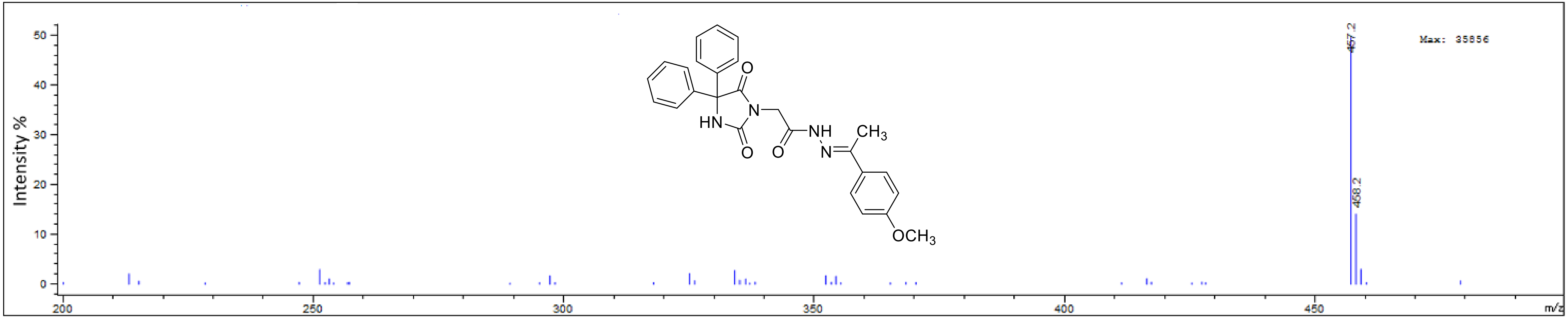

FTIR spectra of compound 16

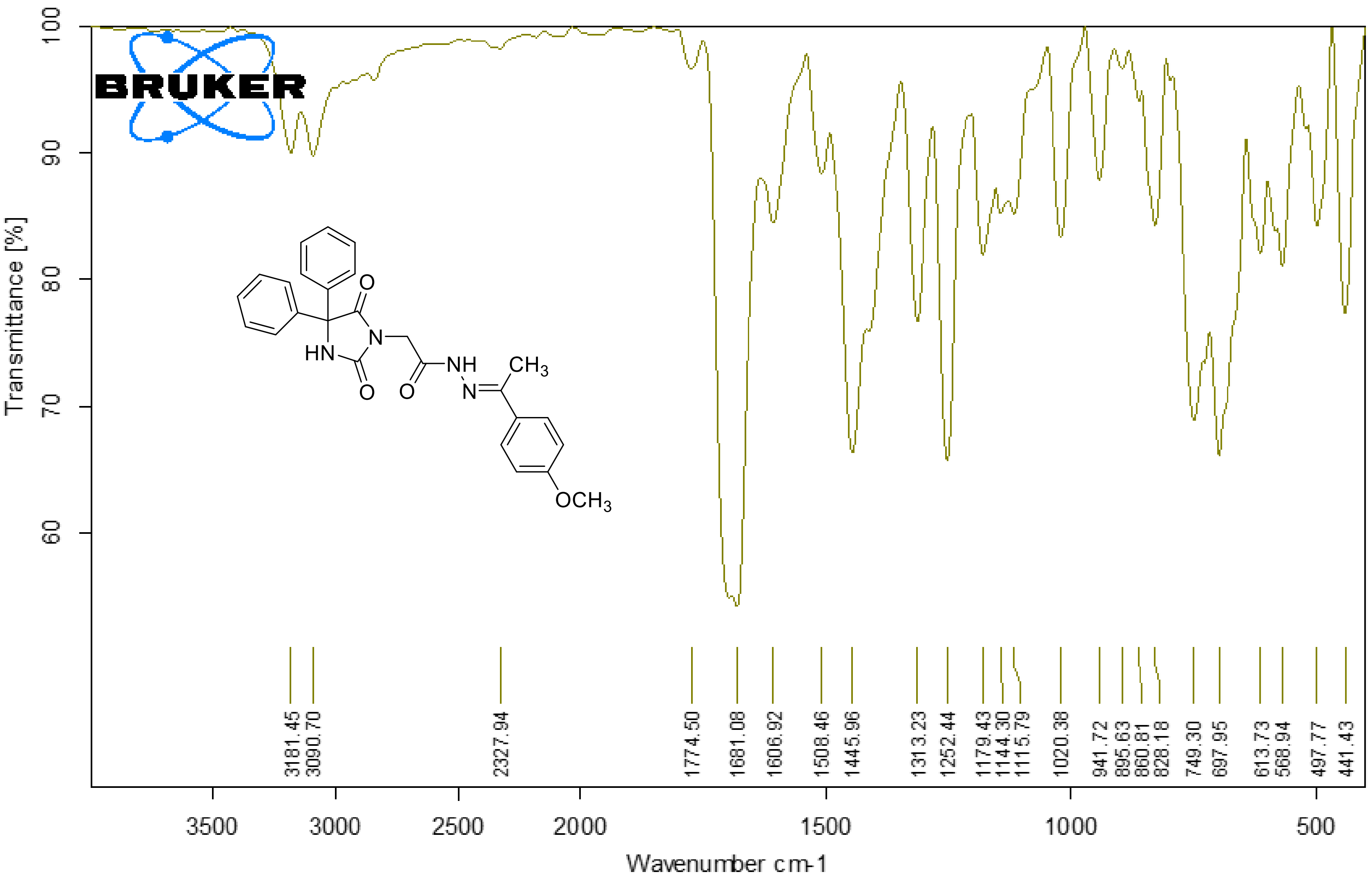

2-(2,5-Dioxo-4,4-diphenylimidazolidin-1-yl)-N′-(1-(3,4,5-trimethoxyphenyl)ethylidene)acetohydrazide (18):

^{1}H-NMR spectra of compound 18

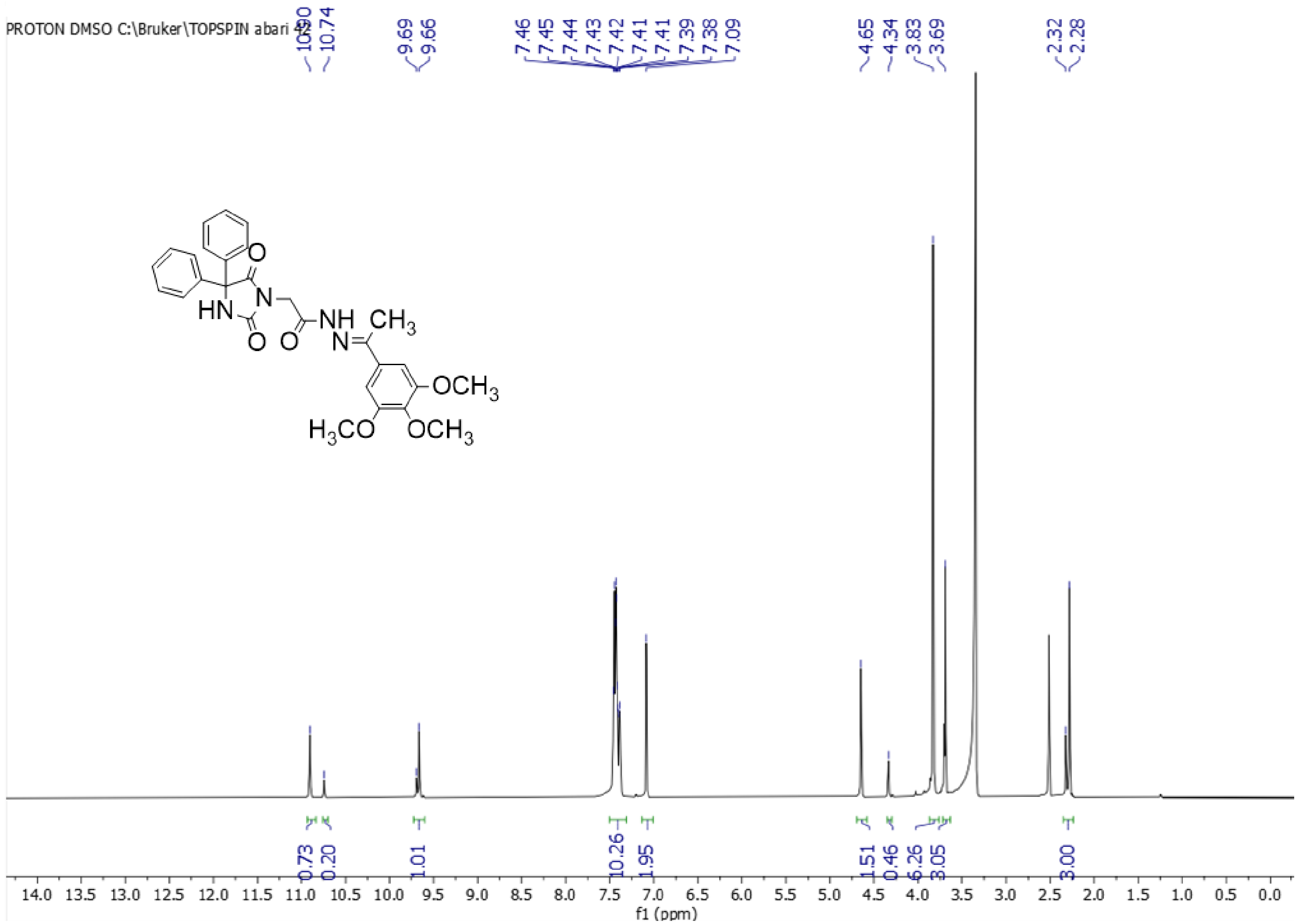

¹³C-NMR spectra of compound 18

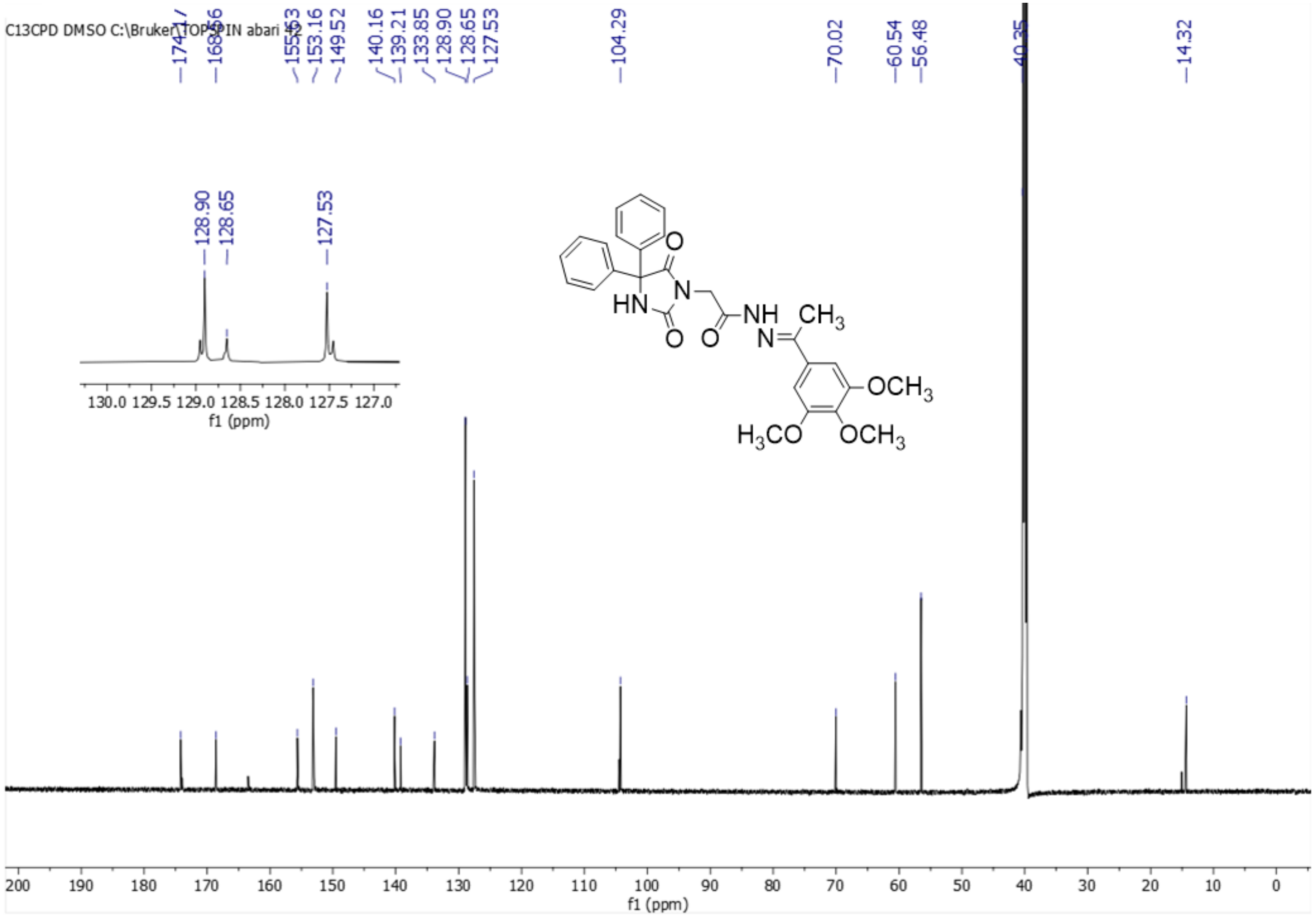

Mass spectra of compound 18

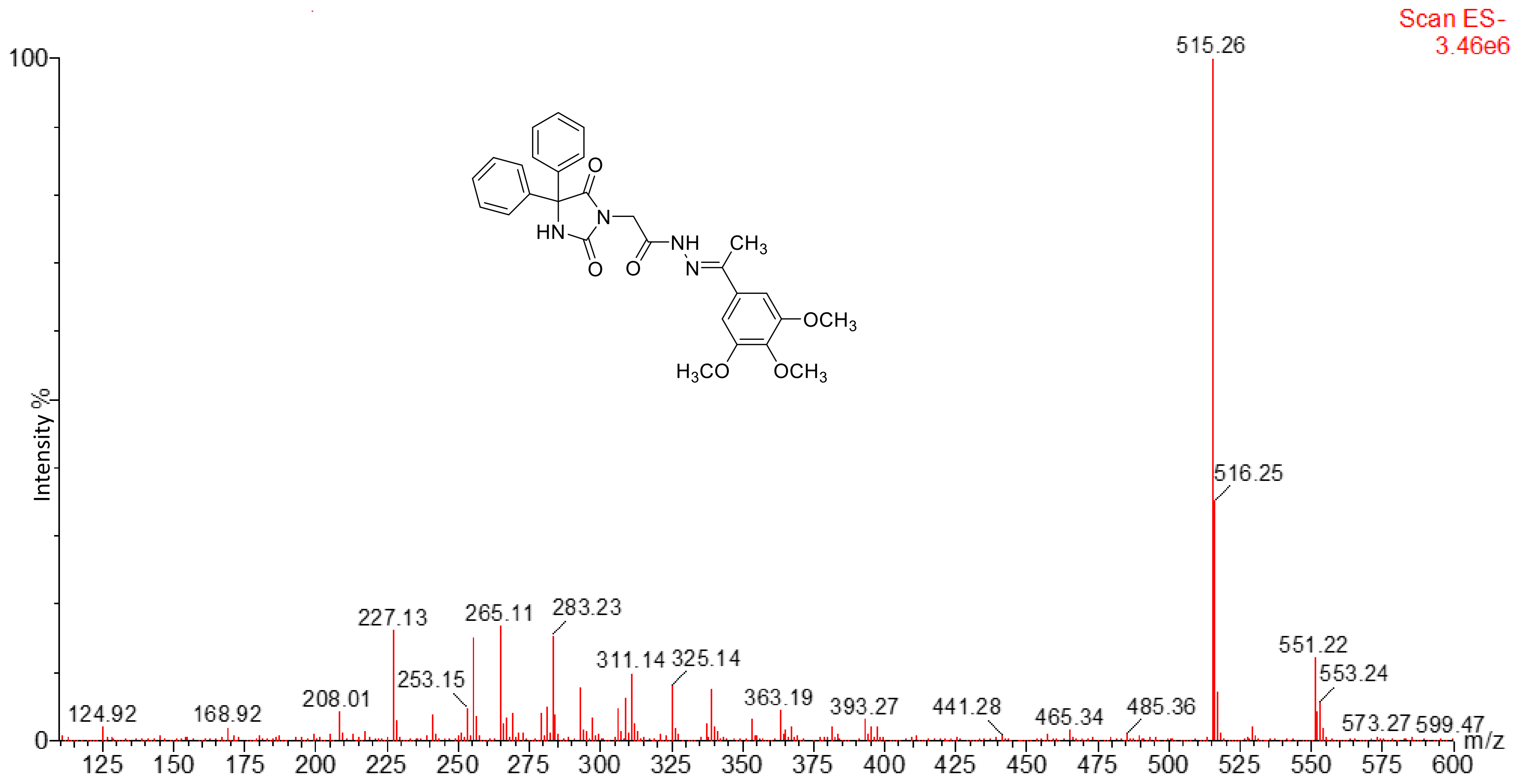

FTIR spectra of compound 18

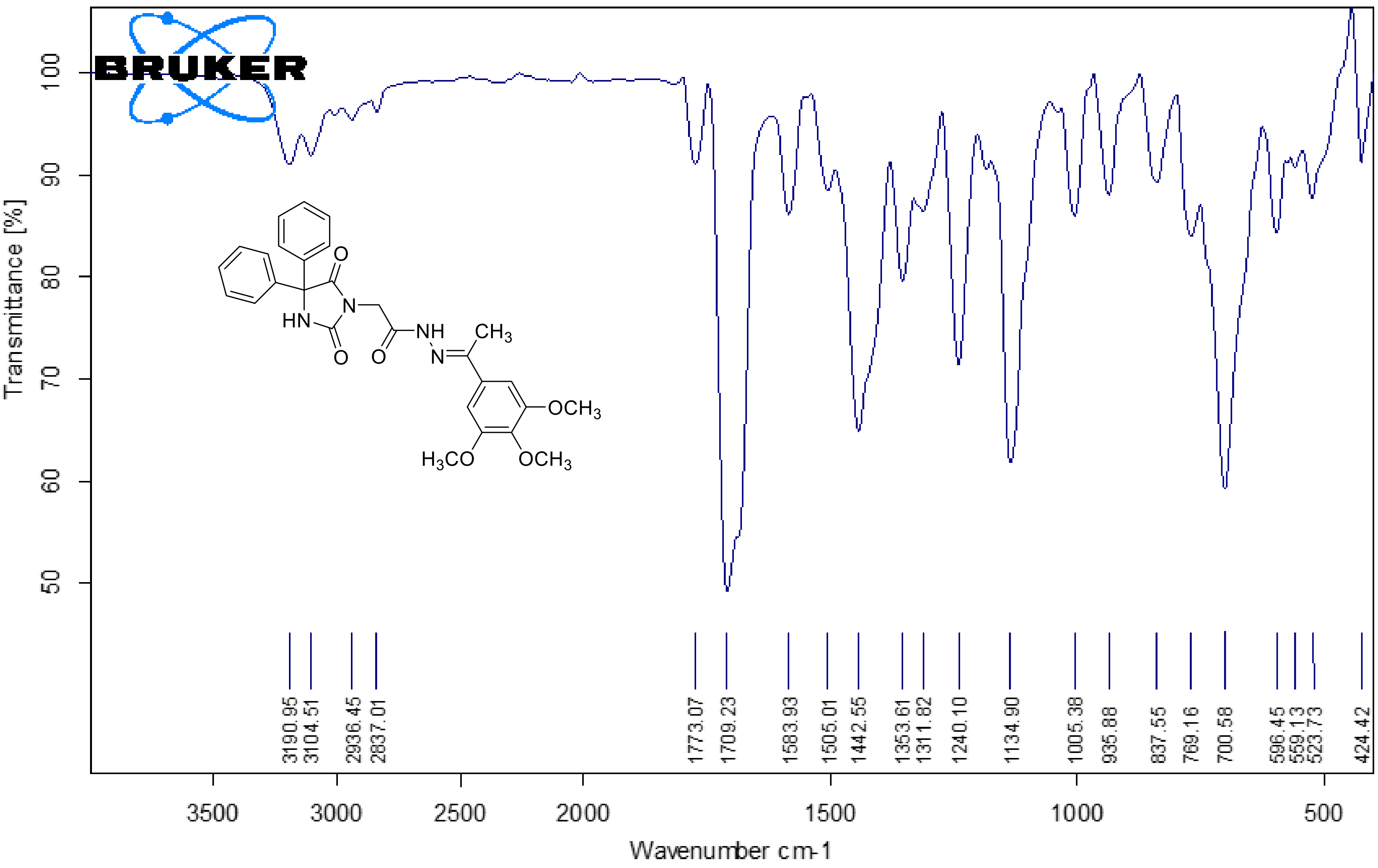

2-(2,5-Dioxo-4,4-diphenylimidazolidin-1-yl)-N′-(1-(pyridin-4-yl)ethylidene)acetohydrazide (22):

^{1}H-NMR spectra of compound 22

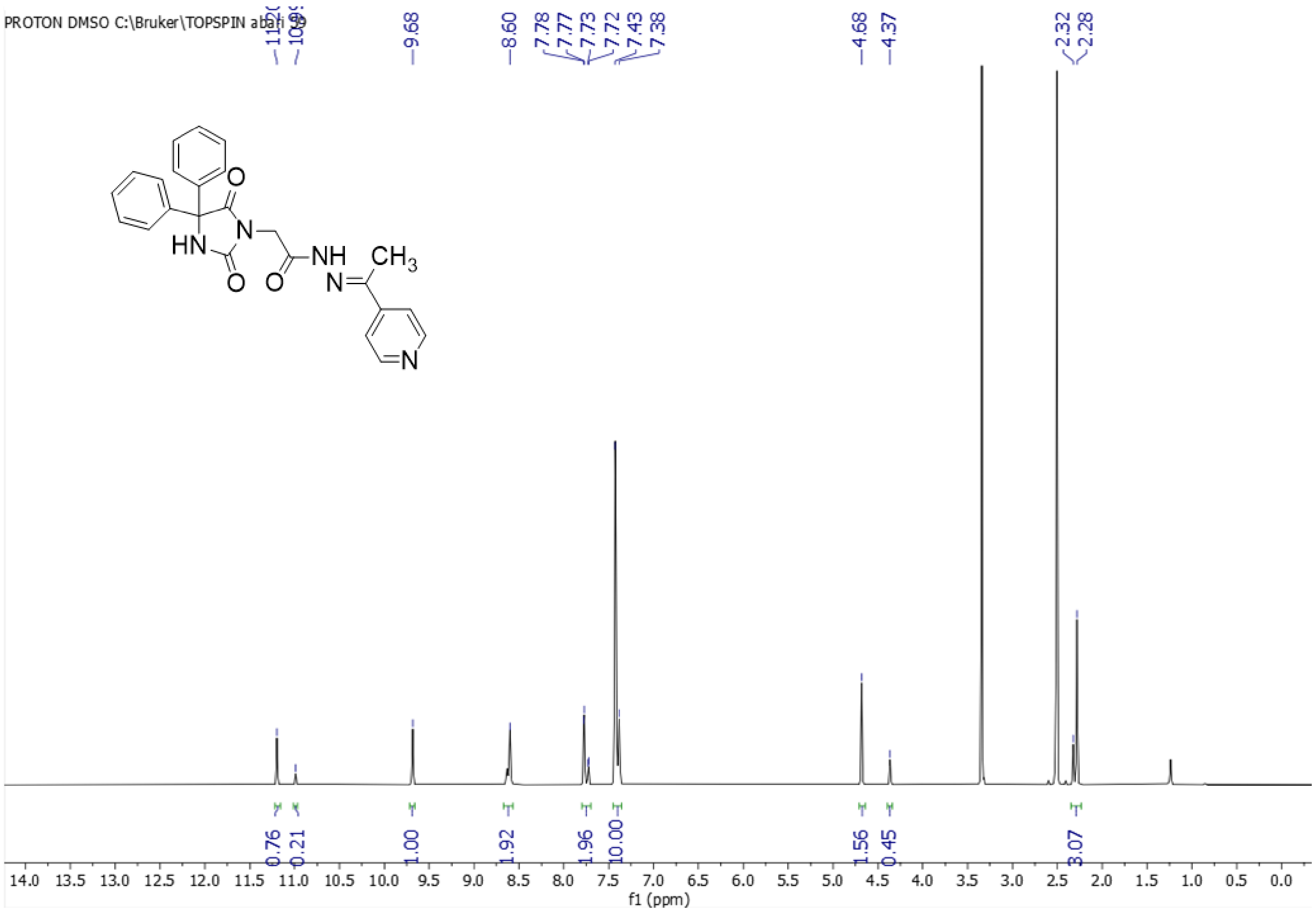

^{13}C-NMR spectra of compound 22

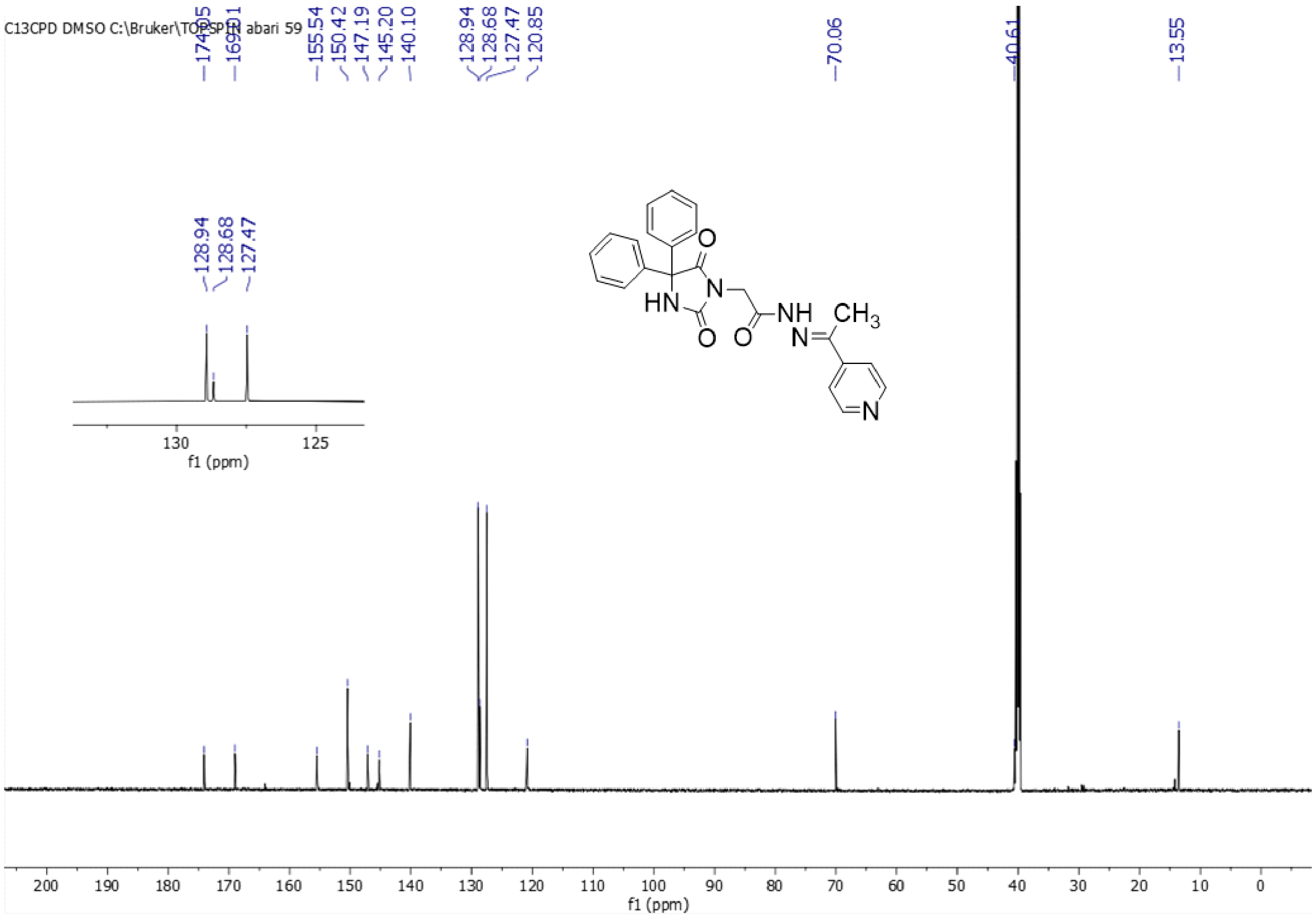

Mass spectra of compound 22

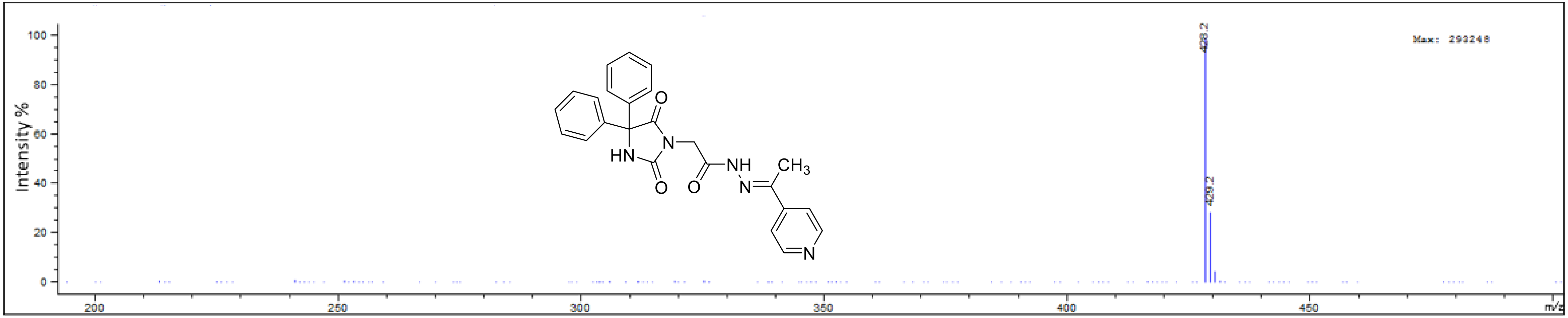

FTIR spectra of compound 22

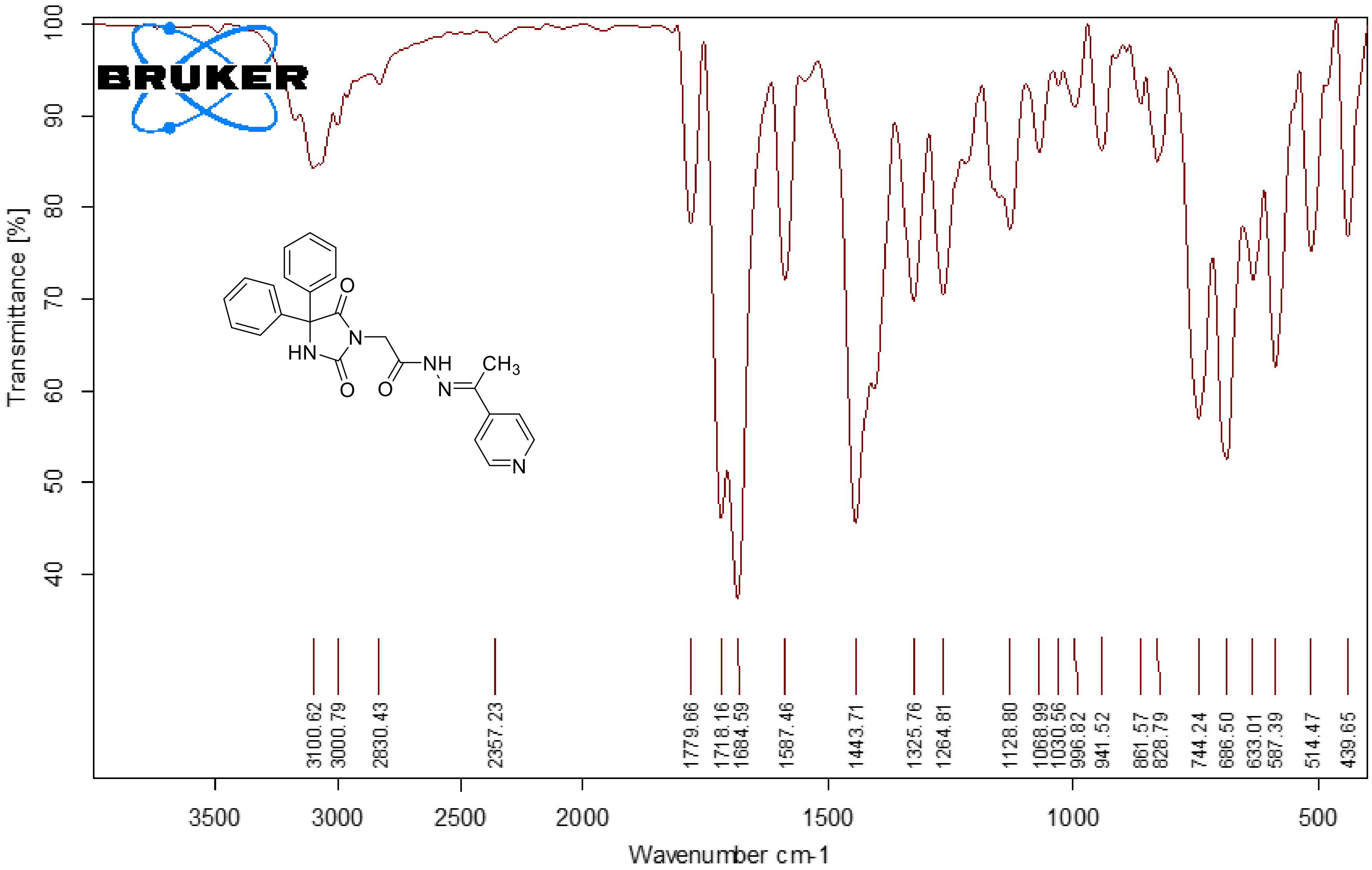

***2-(2,5-Dioxo-4,4-diphenylimidazolidin-1-yl)-N′-(1-(naphthalen-2-yl)ethylidene)acetohydrazide* (24):**

^{1}H-NMR spectra of compound 24

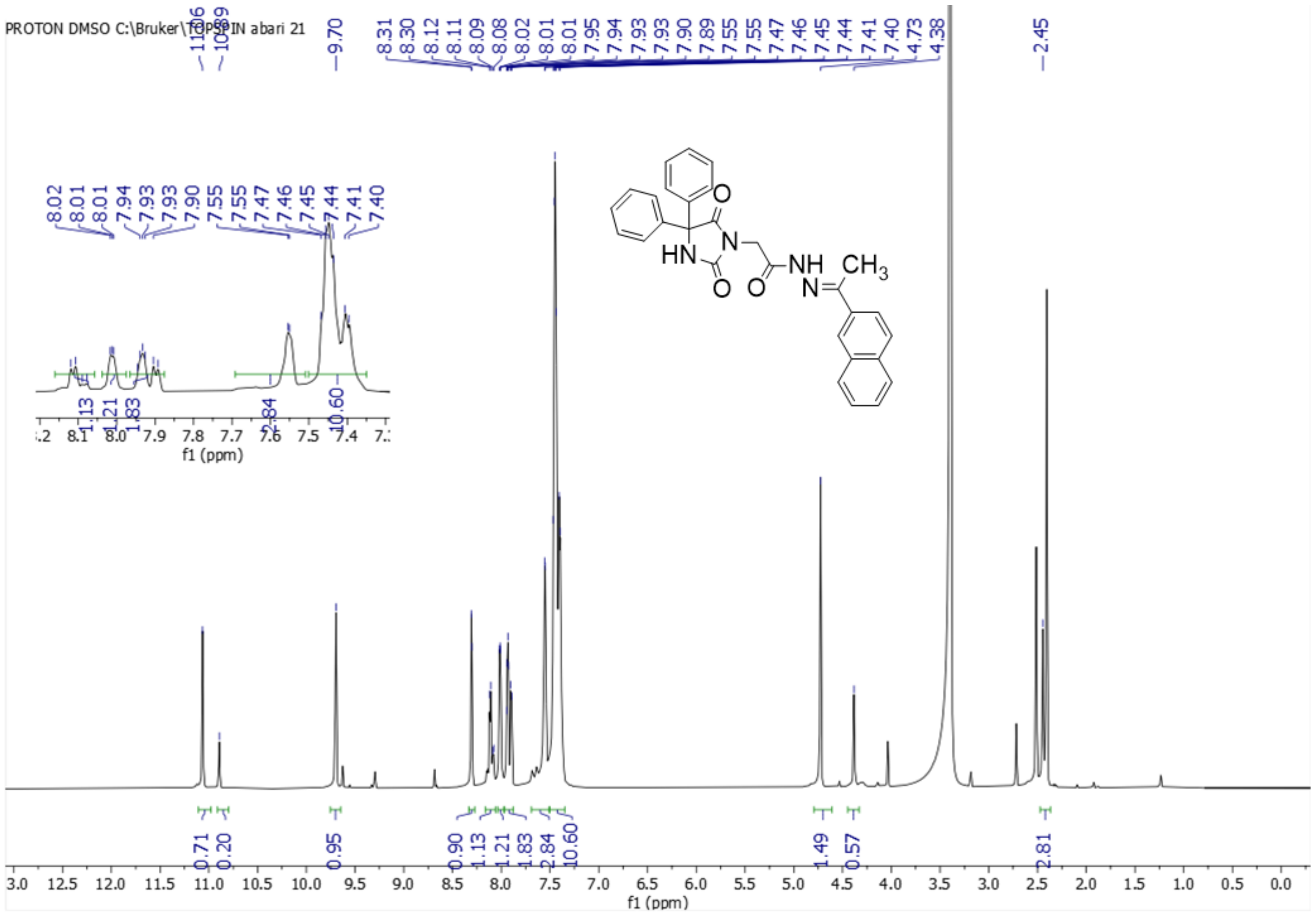

^{13}C-NMR spectra of compound 24

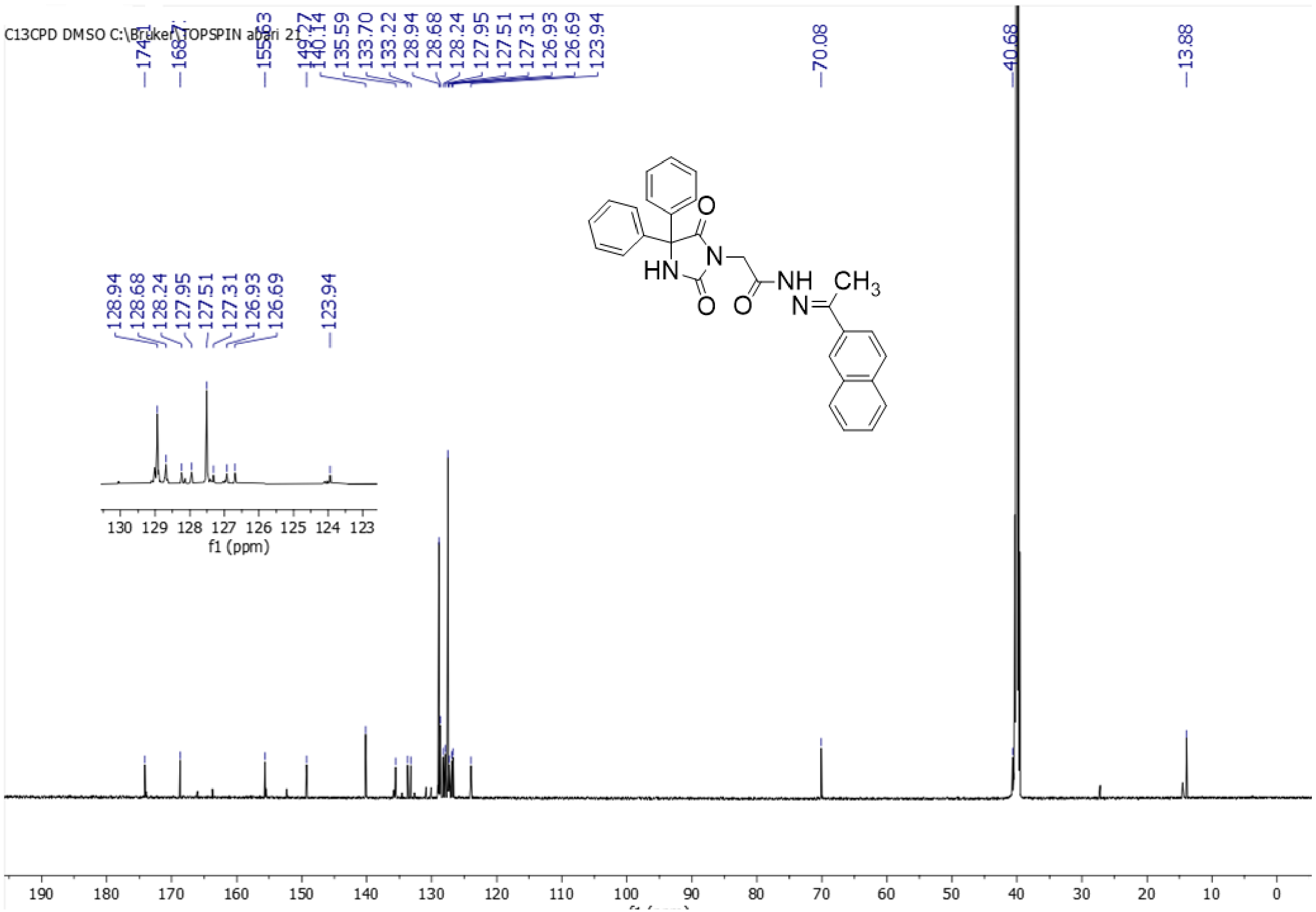

Mass spectra of compound 24

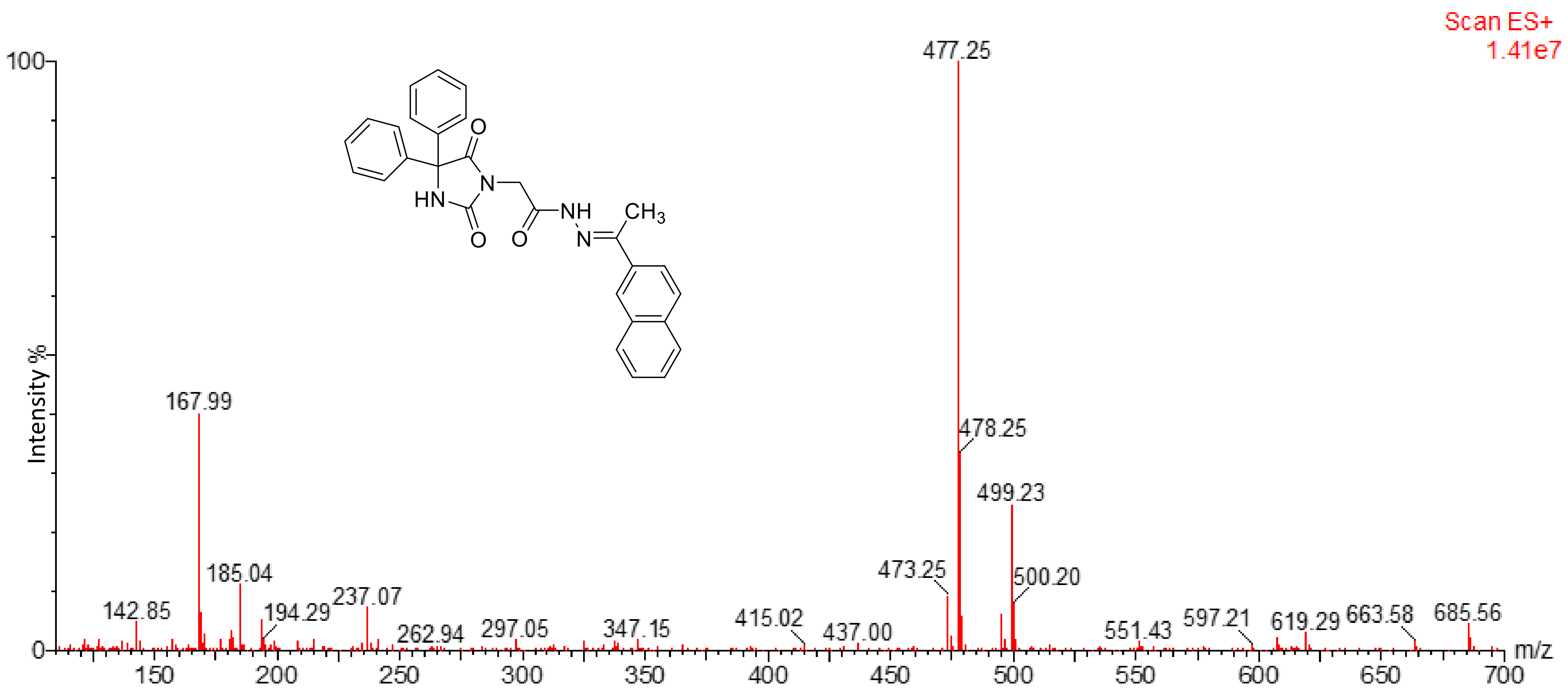

FTIR spectra of compound 24

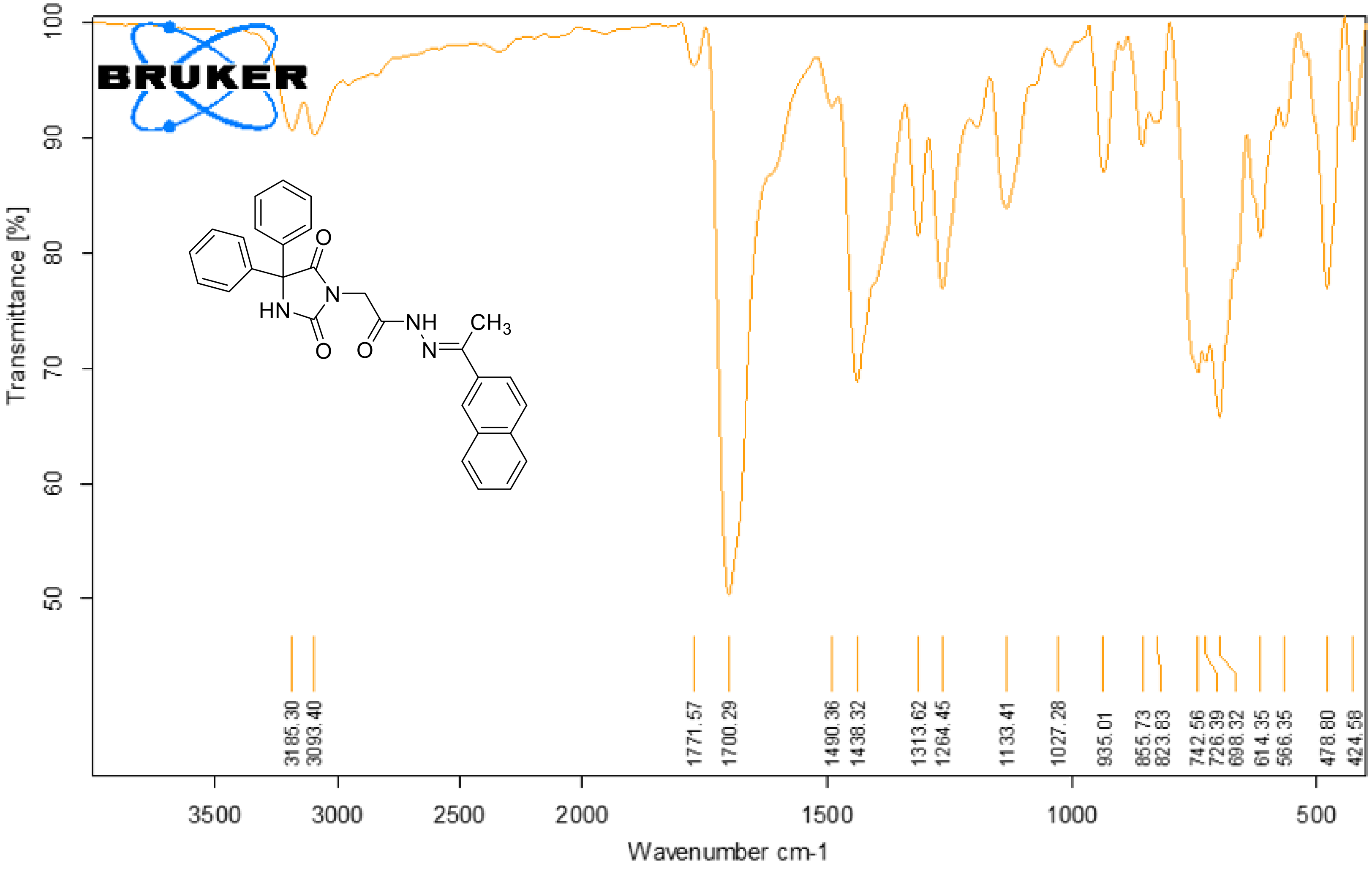

Pharmacokinetics

Table S1. Overview of the Physicochemical Characteristics of the Investigated Compounds.

No.	MW	nHA	nHD	TPSA	LogS	LogD	LogP	Density	Lipinski
**4**	426.17	7	2	90.87	-4.44	3.85	3.664	0.962	Accepted
**5**	440.18	7	2	90.87	-4.757	4.162	4.007	0.957	Accepted
**6**	440.18	7	2	90.87	-4.812	4.2	4.054	0.957	Accepted
**7**	460.13	7	2	90.87	-4.708	4.437	4.221	1.005	Accepted
**8**	444.16	7	2	90.87	-4.542	3.876	3.785	0.989	Accepted
**9**	494.16	7	2	90.87	-5.027	4.504	4.361	1.033	Accepted
**10**	510.15	8	2	100.1	-5.249	4.424	4.655	1.047	Accepted
**11**	456.18	8	2	100.1	-4.621	3.779	3.563	0.973	Accepted
**12**	456.18	8	2	100.1	-4.699	3.811	3.767	0.973	Accepted
**13**	456.18	8	2	100.1	-4.714	3.738	3.725	0.973	Accepted
**14**	486.19	9	2	109.33	-4.787	3.545	3.465	0.982	Accepted
**15**	516.2	10	2	118.56	-4.866	3.432	3.393	0.991	Accepted
**16**	471.15	10	2	134.01	-4.515	4.115	3.612	1.005	Accepted
**17**	427.16	8	2	103.76	-3.745	2.838	2.906	0.979	Accepted
**18**	427.16	8	2	103.76	-3.603	2.517	2.828	0.979	Accepted
**19**	427.16	8	2	103.76	-3.675	2.736	2.906	0.979	Accepted
**20**	476.18	7	2	90.87	-5.134	4.586	4.566	0.956	Accepted
**21**	476.18	7	2	90.87	-4.945	4.628	4.509	0.956	Accepted
**22**	416.15	8	2	104.01	-3.992	3.25	3.171	0.992	Accepted
**23**	432.13	7	2	90.87	-4.512	3.852	3.642	1.006	Accepted
**24**	466.09	7	2	90.87	-4.826	4.253	4.133	1.048	Accepted

Table S2: The Pharmacokinetics Properties of the Synthesized Compounds.

No.	Caco-2	HIA	MDCK	Pgp-inh	Pgp-sub	VDss	BBB	PPB	CL	T½
**4**	-5.508	0.054	2.79E-05	0.97	0.907	0.216	0.99	0.974	0.848	0.64
**5**	-5.419	0.009	3.25E-05	0.991	0.963	0.25	0.96	0.978	0.895	0.465
**6**	-5.322	0.007	2.56E-05	0.995	0.976	0.237	0.946	0.978	0.905	0.547
**7**	-5.315	0.011	2.65E-05	0.992	0.812	0.233	0.966	0.983	0.906	0.52
**8**	-5.234	0.008	2.72E-05	0.994	0.71	0.197	0.933	0.977	0.849	0.368
**9**	-5.327	0.007	2.38E-05	0.996	0.92	0.286	0.891	0.982	0.99	0.178
**10**	-5.263	0.005	0.000029	0.997	0.492	0.346	0.921	0.992	1.08	0.499
**11**	-5.464	0.01	3.13E-05	0.99	0.883	0.31	0.957	0.977	0.811	0.601
**12**	-5.506	0.012	2.34E-05	0.992	0.861	0.306	0.967	0.977	0.866	0.629
**13**	-5.461	0.009	0.000023	0.992	0.877	0.284	0.953	0.977	0.861	0.568
**14**	-5.412	0.007	2.32E-05	0.993	0.819	0.329	0.758	0.977	1.152	0.738
**15**	-5.358	0.017	2.01E-05	0.99	0.536	0.416	0.397	0.976	1.5	0.734
**16**	-5.434	0.035	0.000135	0.914	0.839	0.28	0.069	0.978	0.833	0.433
**17**	-5.331	0.017	3.12E-05	0.951	0.576	0.399	0.991	0.959	0.781	0.57
**18**	-5.545	0.027	2.96E-05	0.913	0.622	0.509	0.984	0.954	0.764	0.726
**19**	-5.676	0.056	2.87E-05	0.929	0.645	0.483	0.976	0.946	0.855	0.73
**20**	-5.413	0.082	2.96E-05	0.993	0.958	0.191	0.46	0.989	0.886	0.303
**21**	-5.405	0.067	2.57E-05	0.994	0.964	0.208	0.44	0.99	0.84	0.425
**22**	-5.933	0.027	2.16E-05	0.904	0.834	1.049	0.982	0.969	1.038	0.668
**23**	-4.941	0.948	2.88E-05	0.885	0.018	0.337	0.911	0.973	0.923	0.402
**24**	-5.402	0.021	2.44E-05	0.36	0.24	0.346	0.806	0.978	1.031	0.196

Table S3: Overview of the Metabolic Probabilities of being enzyme substrate or inhibitor for the Synthesized Compounds.

Comp. No.	CYP1A2 substrate	CYP2C19 inhibitor	CYP2C19 substrate	CYP2C9 inhibitor	CYP2C9 substrate	CYP3A4-inhibitor	CYP3A4-substrate
**4**	0.63	0.898	0.941	0.933	0.975	0.649	0.939
**5**	0.72	0.926	0.946	0.941	0.973	0.852	0.941
**6**	0.763	0.902	0.945	0.94	0.971	0.826	0.941
**7**	0.839	0.942	0.931	0.946	0.972	0.811	0.942
**8**	0.756	0.914	0.927	0.934	0.974	0.743	0.937
**9**	0.777	0.922	0.93	0.947	0.969	0.792	0.935
**10**	0.853	0.894	0.925	0.955	0.969	0.578	0.94
**11**	0.722	0.928	0.951	0.94	0.968	0.859	0.942
**12**	0.758	0.943	0.945	0.939	0.968	0.9	0.939
**13**	0.811	0.916	0.945	0.941	0.972	0.88	0.939
**14**	0.805	0.879	0.954	0.927	0.941	0.87	0.944
**15**	0.876	0.792	0.954	0.908	0.848	0.85	0.949
**16**	0.462	0.792	0.884	0.926	0.972	0.741	0.936
**17**	0.504	0.638	0.943	0.837	0.981	0.263	0.937
**18**	0.642	0.783	0.925	0.892	0.944	0.878	0.939
**19**	0.529	0.903	0.882	0.946	0.891	0.916	0.94
**20**	0.792	0.937	0.927	0.956	0.972	0.911	0.941
**21**	0.729	0.917	0.905	0.942	0.965	0.856	0.939
**22**	0.469	0.876	0.926	0.933	0.957	0.499	0.937
**23**	0.766	0.945	0.932	0.936	0.966	0.805	0.941
**24**	0.839	0.936	0.931	0.948	0.967	0.847	0.942

<0.5: Lower in the probability of being enzyme substrate or inhibitor.
>0.5: Higher in the probability of being enzyme substrate or inhibitor.

Table S4: Molecular docking interactions of compounds 7–27 with the HER2. The table details the specific ligand–receptor contacts, including hydrogen bond donors/acceptors and π-interactions, along with their corresponding distances (Å) and interaction energies (E and S in kcal/mol), highlighting the key binding features that contribute to the compounds' overall affinity for the HER2 target."

Compounds	Ligand	Receptor	Interaction	Distance	E (kcal/mol)	S (kcal/mol)
Comp. 7	O 1	N MET 801 (A)	H-acceptor	3.32	-1.3	-7.63
	6-ring	CD2 PHE 864 (A)	pi-H	4.3	-0.5	
Comp. 8	O 1	N MET 801 (A)	H-acceptor	3.27	-1.8	-7.59
	6-ring	CB CYS 805 (A)	pi-H	3.37	-0.4	
	6-ring	CD2 PHE 864 (A)	pi-H	4.23	-0.4	
Comp. 9	O 1	N MET 801 (A)	H-acceptor	3.18	-2.4	-8.47
	6-ring	CD1 LEU 785 (A)	pi-H	4.48	-0.3	
	6-ring	CB CYS 805 (A)	pi-H	3.47	-0.5	
	6-ring	CD2 PHE 864 (A)	pi-H	4.15	-0.5	
Comp. 10	O 1	N MET 801 (A)	H-acceptor	3.29	-1.8	-8.58
	6-ring	CB CYS 805 (A)	pi-H	3.41	-0.5	
	6-ring	CD2 PHE 864 (A)	pi-H	4.22	-0.5	
Comp. 11	O 1	N MET 801 (A)	H-acceptor	3.29	-1.6	-8.1
	6-ring	CD2 PHE 864 (A)	pi-H	4.18	-0.3	
Comp. 12	O 1	N MET 801 (A)	H-acceptor	3.23	-2.1	-8.54
	6-ring	CB LEU 726 (A)	pi-H	4.2	-0.4	
Comp. 13	O 1	N MET 801 (A)	H-acceptor	3.3	-1.6	-9.19
Comp. 14	O 1	N MET 801 (A)	H-acceptor	3.27	-1.8	-8.25
	6-ring	CB CYS 805 (A)	pi-H	4.02	-0.5	
	6-ring	CB PHE 864 (A)	pi-H	4.8	-0.3	
Comp. 15	C 4	O GLN 799 (A)	H-donor	3.35	-0.6	-7.82
	C 53	OD2 ASP 863 (A)	H-donor	3.2	-0.3	
	O 1	CA LEU 800 (A)	H-acceptor	3.42	-0.5	
	O 1	N MET 801 (A)	H-acceptor	3.21	-2.6	
	6-ring	CB LEU 726 (A)	pi-H	3.98	-0.9	
	6-ring	CD2 PHE 864 (A)	pi-H	3.92	-0.4	
Comp. 16	O 1	N MET 801 (A)	H-acceptor	3.46	-0.8	-7.75
	6-ring	CB LEU 726 (A)	pi-H	4.15	-0.3	
	6-ring	CD1 LEU 785 (A)	pi-H	4.56	-0.3	
	6-ring	CD2 PHE 864 (A)	pi-H	4.1	-0.6	

Cont. Table S4: Molecular docking interactions of compounds 7–27 with the HER2. The table details the specific ligand–receptor contacts, including hydrogen bond donors/acceptors and π-interactions, along with their corresponding distances (Å) and interaction energies (E and S in kcal/mol), highlighting the key binding features that contribute to the compounds' overall affinity for the HER2 target."

**Comp. 17**	**O 1**	**N MET 801 (A)**	**H-acceptor**	**3.34**	**-1.3**	**-8.7**
	6-ring	CB LEU 726 (A)	pi-H	4.2	-0.4	
	6-ring	CD1 LEU 785 (A)	pi-H	4.45	-0.3	
	6-ring	CD2 PHE 864 (A)	pi-H	4.39	-0.4	
**Comp. 18**	O 1	N MET 801 (A)	H-acceptor	3.28	-1.8	-9.54
	6-ring	CD2 PHE 864 (A)	pi-H	4.29	-0.6	
**Comp. 19**	N 8	OG1 THR 862 (A)	H-donor	3.04	-2.2	-8.08
	O 28	N MET 801 (A)	H-acceptor	3.68	-0.4	
	6-ring	CB LEU 726 (A)	pi-H	4.54	-0.3	
	6-ring	CD2 PHE 864 (A)	pi-H	4.07	-0.3	
**Comp. 20**	N 8	OG1 THR 862 (A)	H-donor	2.95	-2.2	-7.26
	O 1	N MET 801 (A)	H-acceptor	3.53	-0.9	
	6-ring	CB VAL 734 (A)	pi-H	3.46	-0.4	
	6-ring	CB PHE 864 (A)	pi-H	4.73	-0.5	
	6-ring	CD2 PHE 864 (A)	pi-H	4.01	-0.4	
**Comp. 21**	O 1	N MET 801 (A)	H-acceptor	3.33	-1.4	-7.94
	6-ring	CD2 PHE 864 (A)	pi-H	4.05	-0.3	
**Comp. 22**	O 1	N MET 801 (A)	H-acceptor	3.43	-1.6	-8.12
	N 10	N ASP 863 (A)	H-acceptor	3.57	-0.7	
	6-ring	CG2 VAL 734 (A)	pi-H	3.59	-0.3	
	6-ring	CB LEU 785 (A)	pi-H	4.56	-0.4	
	6-ring	CD1 LEU 785 (A)	pi-H	4.1	-0.6	
	6-ring	CD2 PHE 864 (A)	pi-H	3.95	-0.4	
**Comp. 23**	O 1	N MET 801 (A)	H-acceptor	3.22	-2.1	-8.49
	6-ring	CB CYS 805 (A)	pi-H	3.35	-0.3	
	6-ring	CD2 PHE 864 (A)	pi-H	4.77	-0.4	
	6-ring	CD2 PHE 864 (A)	pi-H	4.11	-0.4	

Cont. Table S4: Molecular docking interactions of compounds 7–27 with the HER2. The table details the specific ligand–receptor contacts, including hydrogen bond donors/acceptors and π-interactions, along with their corresponding distances (Å) and interaction energies (E and S in kcal/mol), highlighting the key binding features that contribute to the compounds' overall affinity for the HER2 target."

**Comp. 25**	**O 1**	**N MET 801 (A)**	**H-acceptor**	**3.28**	**-1.5**	**-7.61**
**Comp. 26**	N 8	OG1 THR 862 (A)	H-donor	3.38	-0.4	-7.5
	N 19	O ASP 863 (A)	H-donor	3.34	-2.2	
	C 42	O ASP 863 (A)	H-donor	3.38	-0.3	
	S 55	CA LEU 800 (A)	H-acceptor	4.2	-0.6	
	S 55	N MET 801 (A)	H-acceptor	3.62	-3.5	
	6-ring	CD1 LEU 796 (A)	pi-H	3.37	-0.3	
**Comp. 27**	C 46	O MET 801 (A)	H-donor	3.34	-0.3	-6.57
	S 55	CA LEU 800 (A)	H-acceptor	3.95	-0.7	
	S 55	N MET 801 (A)	H-acceptor	3.19	-3.6	
	6-ring	CB PHE 864 (A)	pi-H	4.4	-0.5	
	6-ring	CD2 PHE 864 (A)	pi-H	3.53	-0.4	

Figure S1: Molecular docking interactions of compounds **7–27** with HER2

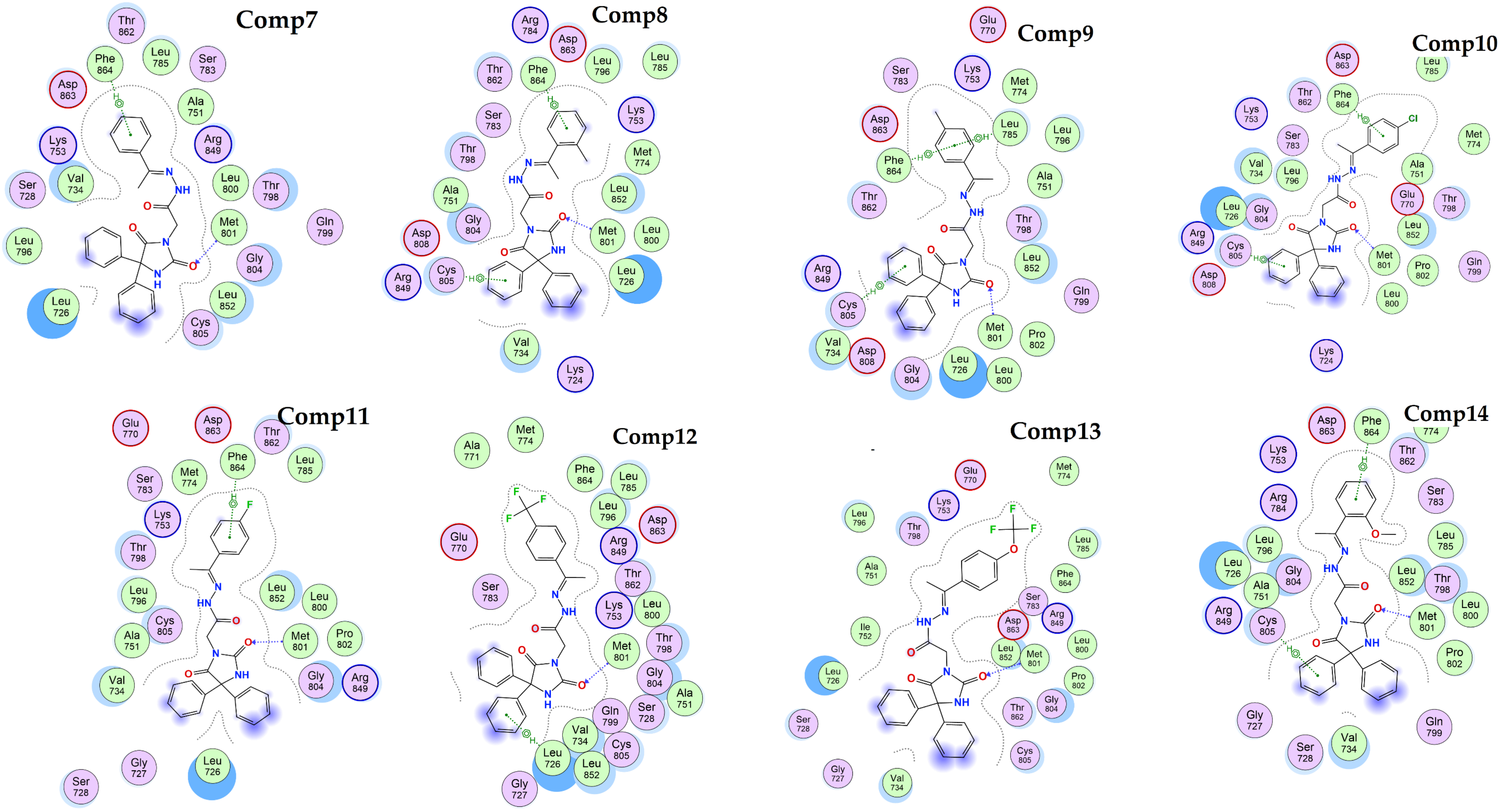

Figure S1: Molecular docking interactions of compounds **7–27** with HER2

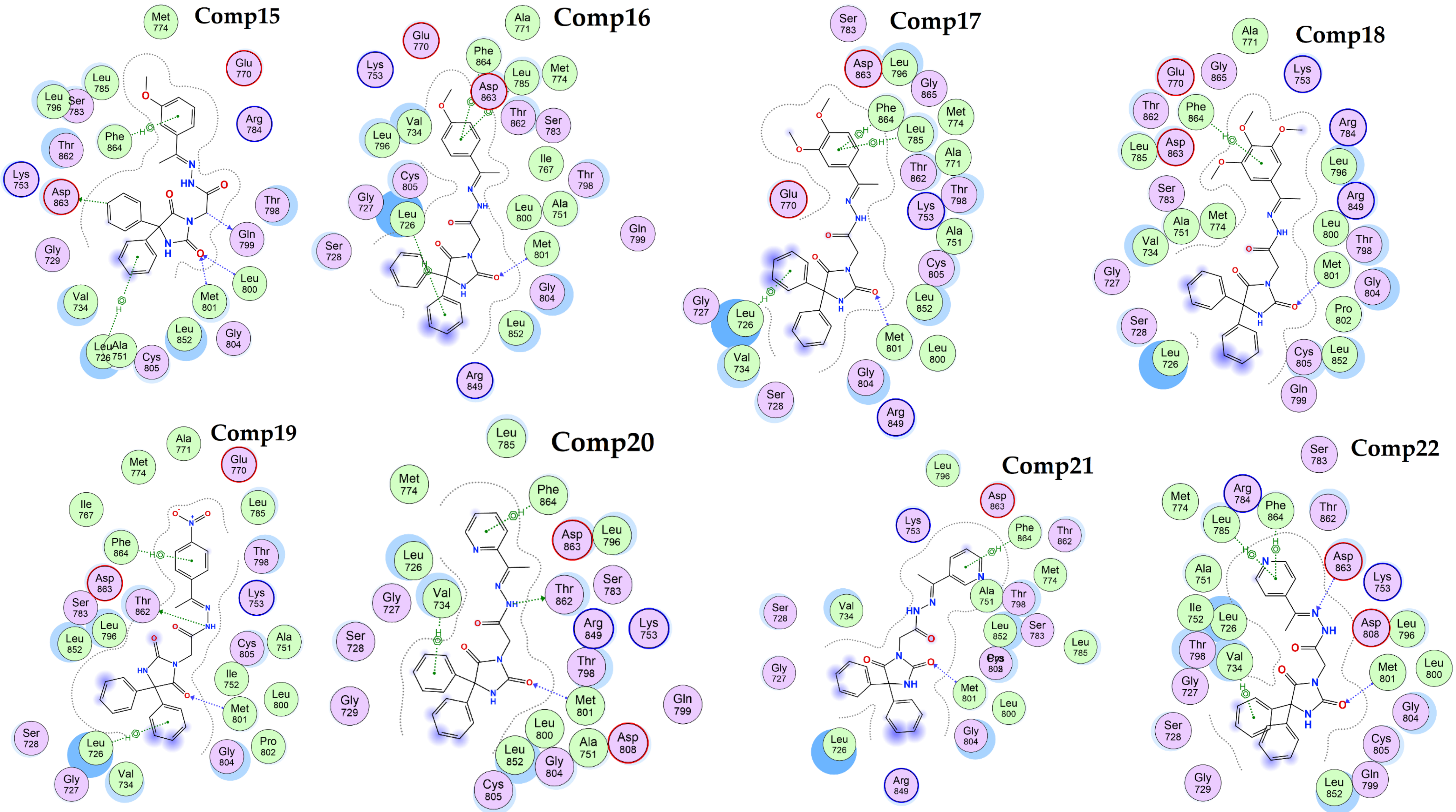

Figure S1: Molecular docking interactions of compounds **7–27** with HER2

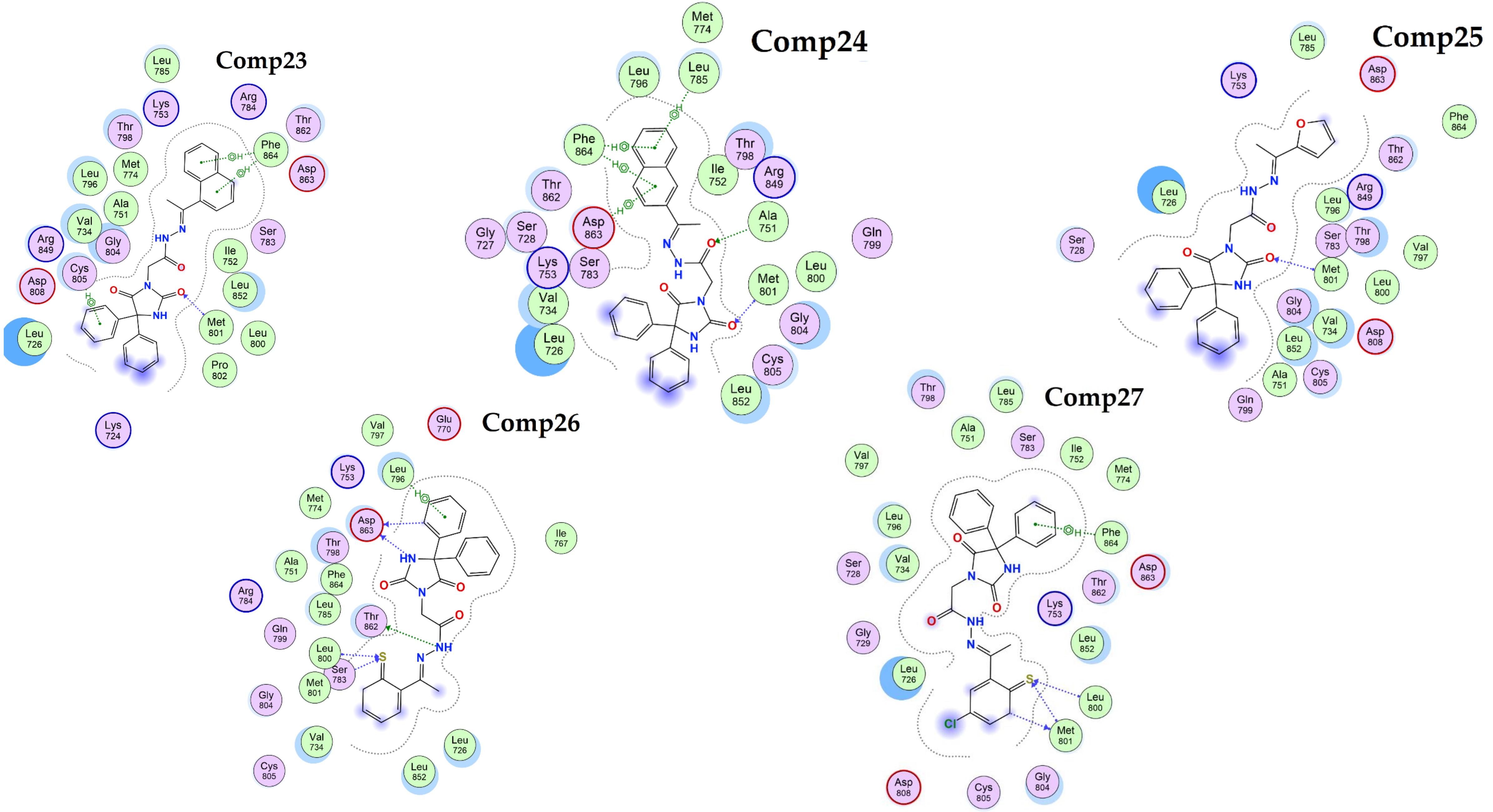

Table S5: Molecular docking interactions of compounds 7–27 with the EGFR kinase domain. The table details the specific ligand–receptor contacts, including hydrogen bond donors/acceptors and π-interactions, along with their corresponding distances (Å) and interaction energies (E and S in kcal/mol), highlighting the key binding features that contribute to the compounds' overall affinity for the EGFR target."

Compounds	Ligand	Receptor	Interaction	Distance	E (kcal/mol)	S (kcal/mol)
Comp7	N 30	OG1 THR 854 (A)	H-donor	3.08	-1.4	-7.28
	C 45	OD2 ASP 855 (A)	H-donor	3.14	-0.3	
	O 27	N MET 793 (A)	H-acceptor	2.94	-2.3	
	6-ring	CD1 LEU 844 (A)	pi-H	4.45	-0.4	
	6-ring	CD2 LEU 844 (A)	pi-H	3.72	-0.5	
Comp8	O 1	SG CYS 775 (A)	H-donor	3.88	-0.6	-7.227
	O 30	N MET 793 (A)	H-acceptor	2.84	-2.3	
	6-ring	CG2 VAL 726 (A)	pi-H	4.61	-0.3	
	6-ring	NZ LYS 745 (A)	pi-cation	4.35	-0.3	
	6-ring	N CYS 797 (A)	pi-H	3.78	-1	
Comp9	N 10	N MET 793 (A)	H-acceptor	3.5	-0.7	-7.56
	O 30	CA LEU 792 (A)	H-acceptor	3.19	-0.4	
	O 30	N MET 793 (A)	H-acceptor	2.73	-2	
Comp10	O 27	N MET 793 (A)	H-acceptor	2.76	-3	-7.818
	6-ring	CD2 LEU 718 (A)	pi-H	4.24	-0.5	
	6-ring	CD2 LEU 844 (A)	pi-H	4.2	-0.5	
Comp11	O 1	SG CYS 775 (A)	H-donor	3.67	-0.3	-7.375
	N 30	OG1 THR 854 (A)	H-donor	3.1	-0.3	
	O 1	OG1 THR 854 (A)	H-acceptor	2.9	-0.4	
	O 27	N MET 793 (A)	H-acceptor	2.74	-4.2	
	6-ring	CB LEU 718 (A)	pi-H	3.93	-0.6	
	6-ring	CD1 LEU 718 (A)	pi-H	4.15	-0.4	
	6-ring	CD2 LEU 718 (A)	pi-H	4.41	-0.6	
Comp12	O 30	N MET 793 (A)	H-acceptor	2.7	-3.4	-7.594
	6-ring	CD1 LEU 718 (A)	pi-H	3.79	-0.3	
	6-ring	CD2 LEU 718 (A)	pi-H	4.11	-0.3	
	6-ring	CD2 LEU 844 (A)	pi-H	4.4	-0.7	

Cont. Table S5: Molecular docking interactions of compounds 7–27 with the EGFR kinase domain. The table details the specific ligand–receptor contacts, including hydrogen bond donors/acceptors and π-interactions, along with their corresponding distances (Å) and interaction energies (E and S in kcal/mol), highlighting the key binding features that contribute to the compounds' overall affinity for the EGFR target."

Comp13	C 53	OE2 GLU 762 (A)	H-donor	3.2	-0.3	-7.199
	O 31	CA LEU 792 (A)	H-acceptor	3.26	-0.4	
	O 31	N MET 793 (A)	H-acceptor	2.76	-2.8	
	6-ring	CD1 LEU 718 (A)	pi-H	3.71	-0.3	
	6-ring	CD2 LEU 718 (A)	pi-H	3.93	-0.3	
	6-ring	CG2 VAL 726 (A)	pi-H	3.48	-0.3	
	6-ring	NZ LYS 745 (A)	pi-cation	3.34	-1.2	
Comp14	O 1	SG CYS 775 (A)	H-donor	3.81	-0.3	-7.627
	N 34	OG1 THR 854 (A)	H-donor	3.11	-0.4	
	O 1	OG1 THR 854 (A)	H-acceptor	2.91	-0.3	
	O 31	N MET 793 (A)	H-acceptor	2.85	-4.4	
	6-ring	CB LEU 718 (A)	pi-H	3.82	-0.9	
	6-ring	CD1 LEU 718 (A)	pi-H	4.19	-0.3	
	6-ring	CD2 LEU 718 (A)	pi-H	4.53	-0.4	
	6-ring	NZ LYS 745 (A)	pi-cation	3.26	-0.3	
Comp15	O 1	SG CYS 775 (A)	H-donor	3.54	-0.5	-7.731
	N 34	OG1 THR 854 (A)	H-donor	3.2	-0.4	
	O 1	OG1 THR 854 (A)	H-acceptor	3.16	-0.3	
	O 31	N MET 793 (A)	H-acceptor	2.83	-3.7	
	6-ring	CB LEU 718 (A)	pi-H	3.9	-1	
	6-ring	CD1 LEU 718 (A)	pi-H	4.36	-0.4	
	6-ring	CD2 LEU 718 (A)	pi-H	4.67	-0.3	
	6-ring	CD LYS 745 (A)	pi-H	3.63	-0.3	
	6-ring	CD1 LEU 844 (A)	pi-H	4.81	-0.3	
	6-ring	CD2 LEU 844 (A)	pi-H	4.61	-0.6	
Comp16	O 1	OG1 THR 854 (A)	H-acceptor	2.74	-0.4	-7.509
	O 31	CA LEU 792 (A)	H-acceptor	3.19	-0.3	
	O 31	N MET 793 (A)	H-acceptor	2.72	-2.1	
	6-ring	CD1 LEU 718 (A)	pi-H	3.62	-0.3	
	6-ring	CD2 LEU 844 (A)	pi-H	4.47	-0.4	

Cont. Table S5: Molecular docking interactions of compounds 7–27 with the EGFR kinase domain. The table details the specific ligand–receptor contacts, including hydrogen bond donors/acceptors and π-interactions, along with their corresponding distances (Å) and interaction energies (E and S in kcal/mol), highlighting the key binding features that contribute to the compounds' overall affinity for the EGFR target."

Comp17	O 1	OG1 THR 854 (A)	H-acceptor	2.74	-0.4	-8.057
	N 10	N MET 793 (A)	H-acceptor	3.52	-1.2	
	O 35	N MET 793 (A)	H-acceptor	2.71	-1.6	
	6-ring	CD2 LEU 844 (A)	pi-H	4.45	-0.4	
Comp18	O 39	N MET 793 (A)	H-acceptor	2.95	-3	-8.213
	6-ring	NZ LYS 745 (A)	pi-cation	3.89	-0.3	
Comp19	N 10	N MET 793 (A)	H-acceptor	3.48	-0.8	-8.042
	O 26	CA LEU 792 (A)	H-acceptor	3.22	-0.3	
	O 26	N MET 793 (A)	H-acceptor	2.73	-1.8	
	O 55	CE LYS 728 (A)	H-acceptor	3.41	-0.6	
	6-ring	CD2 LEU 844 (A)	pi-H	4.42	-0.7	
Comp20	O 1	OG1 THR 854 (A)	H-acceptor	2.69	-0.5	-7.328
	N 10	N MET 793 (A)	H-acceptor	3.66	-0.4	
	O 26	CA LEU 792 (A)	H-acceptor	3.13	-0.5	
	O 26	N MET 793 (A)	H-acceptor	2.76	-2	
	6-ring	CD2 LEU 844 (A)	pi-H	4.41	-0.5	
Comp21	O 16	N MET 793 (A)	H-acceptor	2.71	-3.5	-7.284
	6-ring	CD1 LEU 718 (A)	pi-H	3.91	-0.3	
	6-ring	CD2 LEU 844 (A)	pi-H	4.44	-0.7	
Comp22	O 26	N MET 793 (A)	H-acceptor	2.71	-3.8	-7.075
	6-ring	CD1 LEU 718 (A)	pi-H	3.91	-0.3	
	6-ring	CD2 LEU 718 (A)	pi-H	4.21	-0.9	
Comp23	O 1	SG CYS 775 (A)	H-donor	3.49	-0.4	-8.044
	O 1	OG1 THR 854 (A)	H-acceptor	2.77	-0.7	
	N 10	N MET 793 (A)	H-acceptor	3.23	-2.6	
	6-ring	CG2 VAL 726 (A)	pi-H	4.47	-0.7	

Cont. Table S5: Molecular docking interactions of compounds 7–27 with the EGFR kinase domain. The table details the specific ligand–receptor contacts, including hydrogen bond donors/acceptors and π-interactions, along with their corresponding distances (Å) and interaction energies (E and S in kcal/mol), highlighting the key binding features that contribute to the compounds' overall affinity for the EGFR target."

**Comp25**	**O 1**	**SG CYS 775 (A)**	**H-donor**	**3.69**	**-0.5**	**-7.313**
	N 19	OG1 THR 854 (A)	H-donor	3.22	-0.5	
	O 16	N MET 793 (A)	H-acceptor	2.83	-3.9	
	5-ring	CB LEU 718 (A)	pi-H	3.97	-1.4	
	5-ring	CD1 LEU 718 (A)	pi-H	4.25	-0.3	
	6-ring	CG2 VAL 726 (A)	pi-H	4.6	-0.3	
	6-ring	CD LYS 745 (A)	pi-H	3.65	-0.3	
**Comp26**	O 16	CA LEU 792 (A)	H-acceptor	3.35	-0.3	-7.217
	O 16	N MET 793 (A)	H-acceptor	2.75	-3.4	
	6-ring	CG2 THR 854 (A)	pi-H	3.74	-0.4	
**Comp27**	O 1	SG CYS 775 (A)	H-donor	3.82	-0.7	-7.234
	C 25	SD MET 766 (A)	H-donor	3.79	-0.3	
	S 55	SG CYS 797 (A)	H-donor	3.66	-0.3	
	O 16	N MET 793 (A)	H-acceptor	2.8	-2.5	
	S 55	N CYS 797 (A)	H-acceptor	3.54	-0.3	
	S 55	SG CYS 797 (A)	H-acceptor	3.66	-0.3	

Figure S2: Molecular docking interactions of compounds **7–27** with EGFR

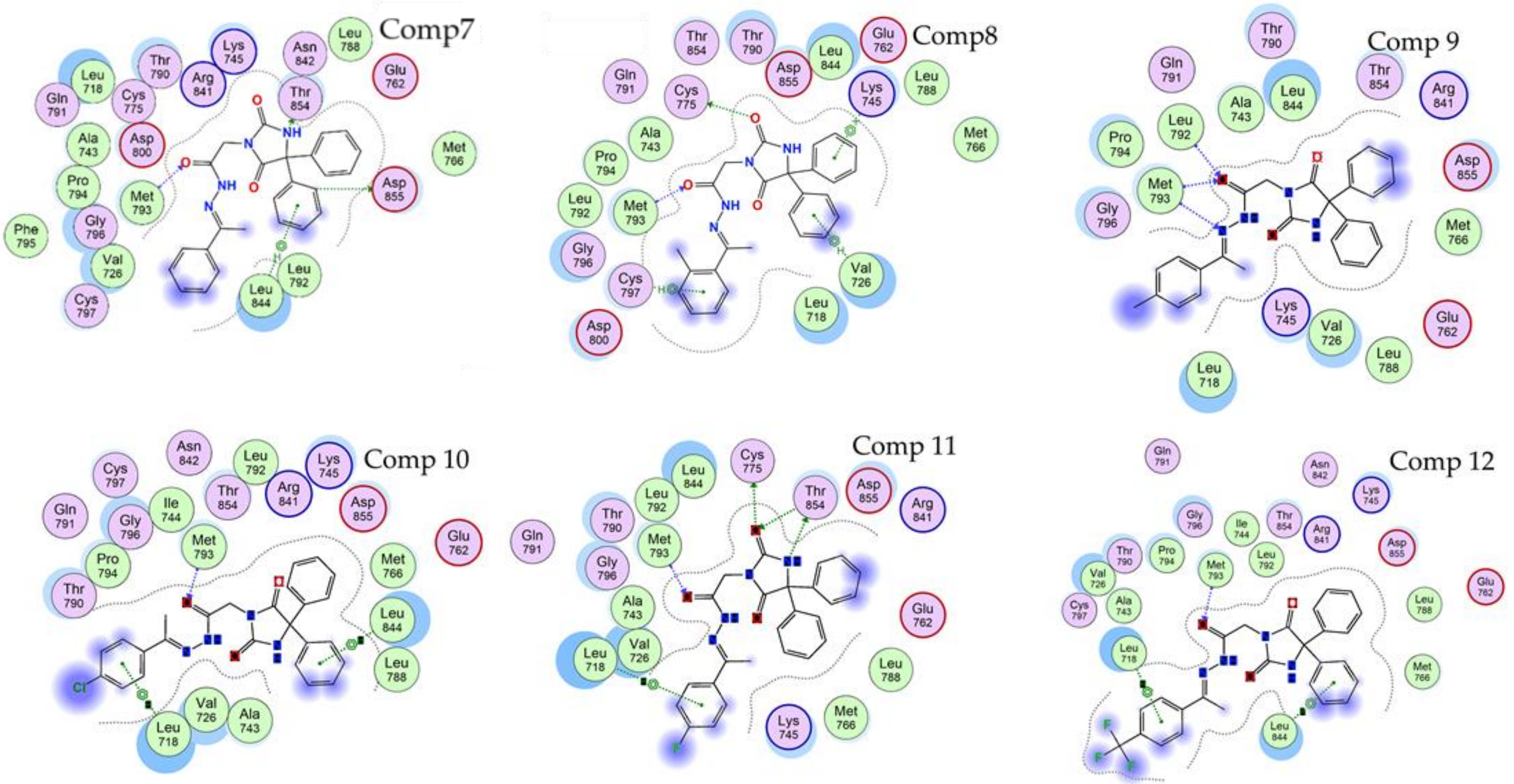

Figure S2: Molecular docking interactions of compounds **7–27** with EGFR

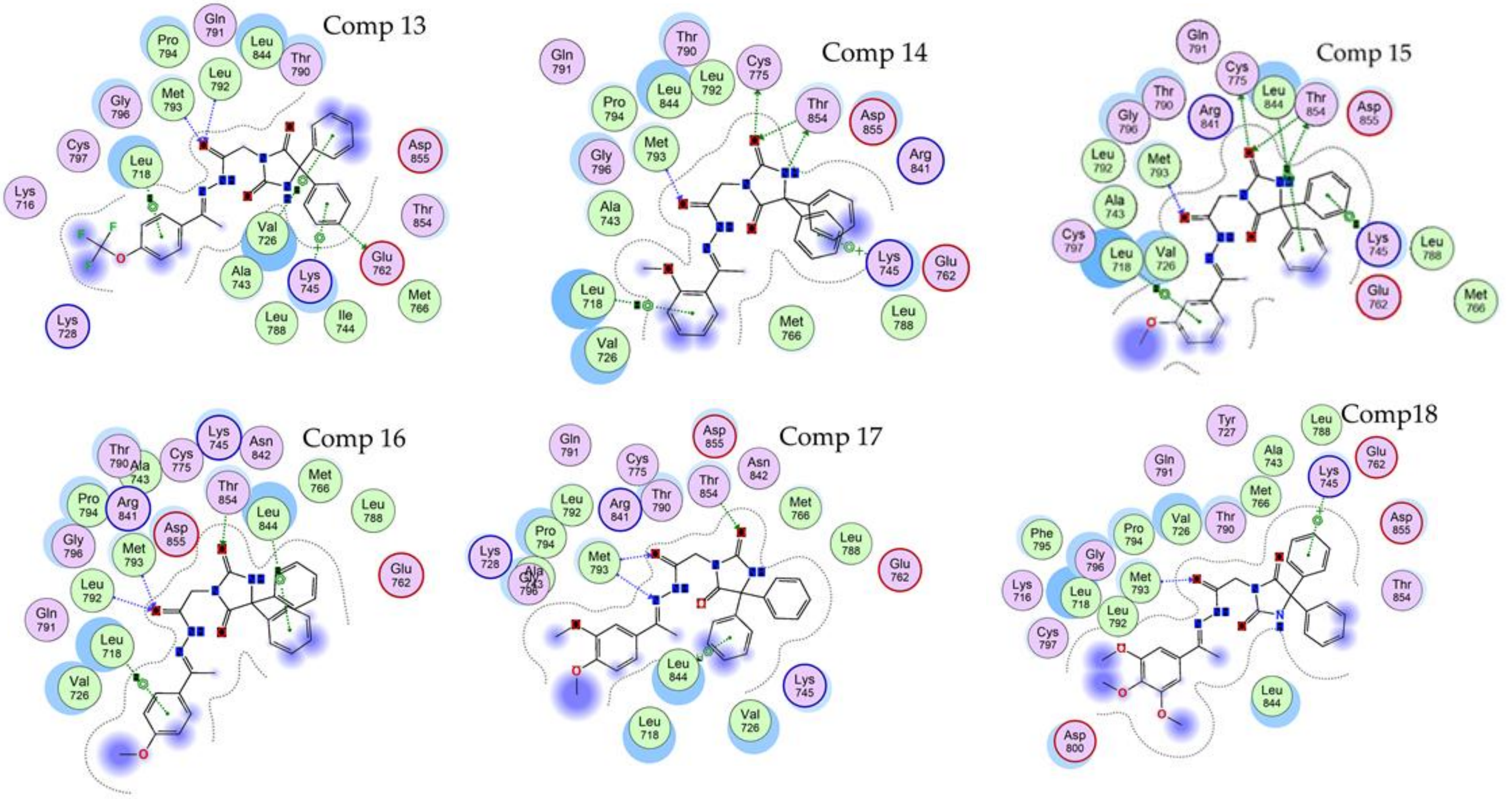

Figure S2: Molecular docking interactions of compounds **7–27** with EGFR

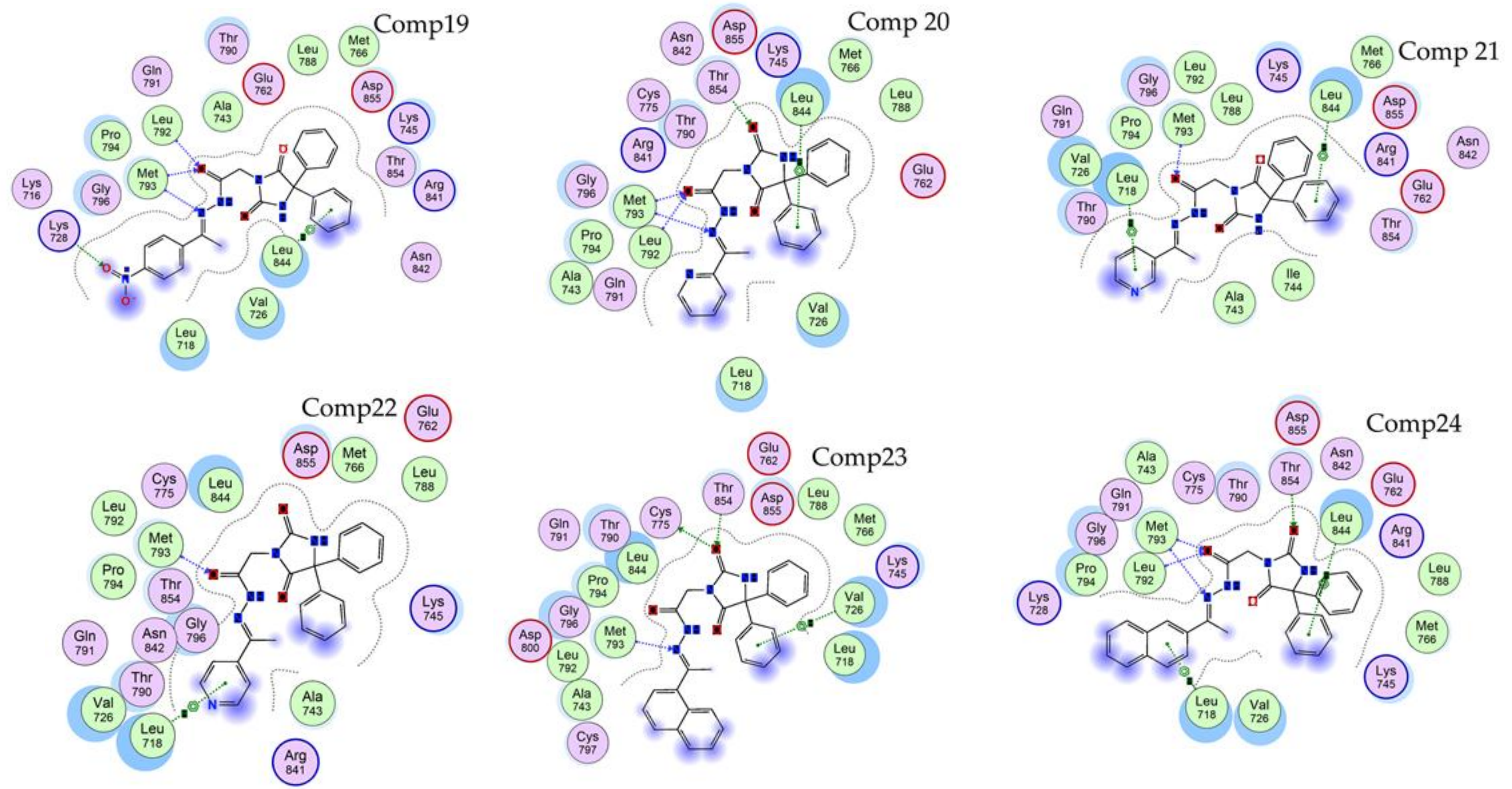

Figure S2: Molecular docking interactions of compounds **7–27** with EGFR

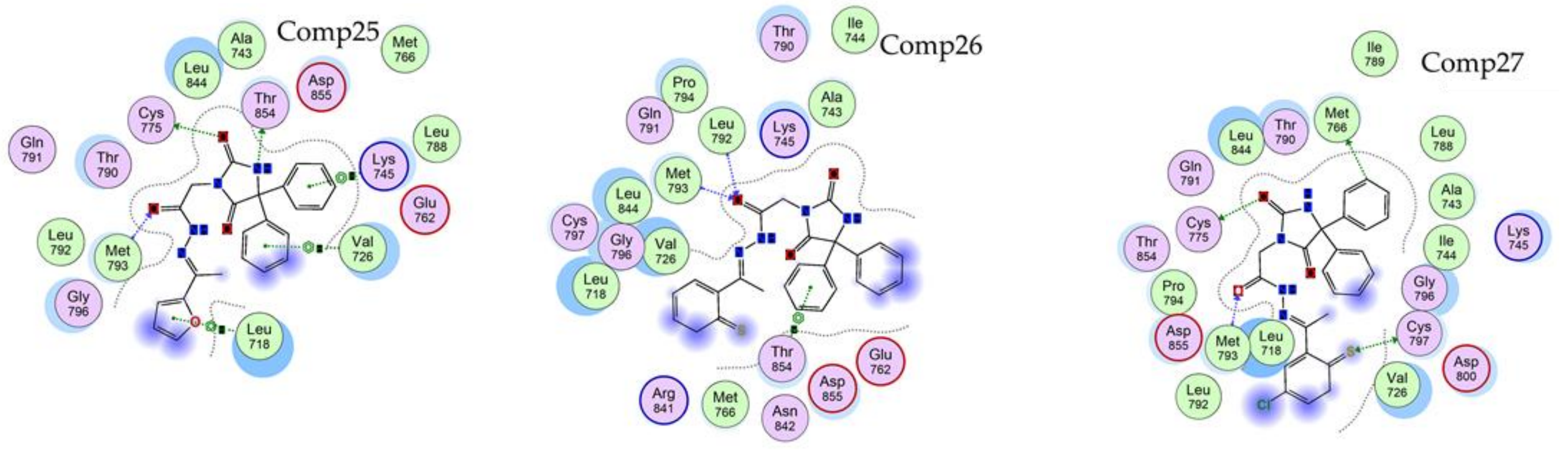

Supplement: Supplementary file 1 [file pharmaceuticals-18-00496-s001.zip › pharmaceuticals-3521239-supplementary.pdf]
